# The use of ecological analytical tools as an unconventional approach for untargeted metabolomics data analysis: the case of *Cecropia obtusifolia* and its adaptive responses to nitrate starvation

**DOI:** 10.1007/s10142-022-00904-1

**Published:** 2022-10-06

**Authors:** Jorge David Cadena-Zamudio, Juan Luis Monribot-Villanueva, Claudia-Anahí Pérez-Torres, Fulgencio Alatorre-Cobos, Beatriz Jiménez-Moraila, José A. Guerrero-Analco, Enrique Ibarra-Laclette

**Affiliations:** 1 Red de Estudios Moleculares Avanzados (REMAV), Instituto de Ecología, A.C. (INECOL), Xalapa, 91073 Veracruz México; 2 Investigador por México - CONACYT en la Red de Estudios Moleculares Avanzados (REMAV), Instituto de Ecología, A.C. (INECOL), Xalapa, 91073 Veracruz México; 3 Investigador por México - CONACYT en el Colegio de Postgraduados (COLPOS) campus Campeche, Sihochac, 24450 Campeche México; 4 Unidad de Genómica Avanzada (UGA-LANGEBIO), Centro de Investigación y de Estudios Avanzados del Instituto Politécnico Nacional (CINVESTAV-IPN), Irapuato, 36824 Guanajuato México

**Keywords:** Mass spectrometry, Metabolic diversity, Metabolic pathways, Metabolite annotation, Plant metabolomics

## Abstract

Plant metabolomics studies haves revealed new bioactive compounds. However, like other omics disciplines, the generated data are not fully exploited, mainly because the commonly performed analyses focus on elucidating the presence/absence of distinctive metabolites (and/or their precursors) and not on providing a holistic view of metabolomic changes and their participation in organismal adaptation to biotic and abiotic stress conditions. Therefore, spectral libraries generated from *Cecropia obtusifolia* cell suspension cultures in a previous study were considered as a case study and were reanalyzed herein. These libraries were obtained from a time-course experiment under nitrate starvation conditions using both electrospray ionization modes. The applied methodology included the use of ecological analytical tools in a systematic four-step process, including a population analysis of metabolite α diversity, richness, and evenness (i); a chemometrics analysis to identify discriminant groups (ii); differential metabolic marker identification (iii); and enrichment analyses and annotation of active metabolic pathways enriched by differential metabolites (iv). Our species α diversity results referring to the diversity of metabolites represented by mass-to-charge ratio (m/z) values detected at a specific retention time (rt) (an uncommon way to analyze untargeted metabolomic data) suggest that the metabolome is dynamic and is modulated by abiotic stress. A total of 147 and 371 m/z_rt pairs was identified as differential markers responsive to nitrate starvation in ESI^−^ and ESI^+^ modes, respectively. Subsequent enrichment analysis showed a high degree of completeness of biosynthetic pathways such as those of brassinosteroids, flavonoids, and phenylpropanoids.

**Supplementary Information:**

The online version contains supplementary material available at 10.1007/s10142-022-00904-1.

## Introduction

Plants have traditionally been used as an essential source of biologically active compounds for hundreds of years. Advances in analytical chemistry and phytochemistry techniques have allowed the isolation, identification, and exploitation of a wide range of natural products and secondary metabolites (SMes) (Lee et al. 2020; Owen et al. 2017) to benefit human health. More than 30% of currently used medications are obtained directly from plants, and more than 60% of the medical drugs introduced in the last 20 years have been based on plant extracts or byproducts (Fang et al. 2019; Rai et al. 2017). These SMes are highly represented in plants. It has been estimated that all plants can collectively synthesize approximately one million metabolites and that a single plant can produce ~ 5000 of these compounds (Rai et al. 2017; Yu et al. 2020). The development of high-throughput technologies allowed the beginning of the metabolomic era, which is used to characterize metabolites based on chemical profiling and statistical tools. In recent years, mass spectrometry (MS) has been considered a highly efficient method for discerning chemotaxonomy, elucidating metabolic pathways, performing phytochemical characterization, and carrying out SMe annotation (Lee et al. 2015; Zhao et al. 2017). However, after performing the corresponding analyses, a gap still exists between the efficiency of data output processing and the generation of conclusive results (Tsugawa 2018). This is mainly due to the challenging procedure for implementing the specialized bioinformatics tools needed, given that the development and constant improvement of platforms commonly used in omics disciplines have generated an increasing demand for novel statistical methods and bioinformatics tools to handle and analyze the huge amounts of data generated by these technologies. Some of the bioinformatic platforms/tools used to analyze this type of data are MetaboAnalyst, MetabolomeExpress, Cytoscape, and free software tools such as RSutdio and Python; these tools offer wide flexibility for the analysis of large amounts of data, such as those generated by metabolomic approaches (Li and Gaquerel 2021; Tsugawa 2018). In recent years, the evolution of the different generations of bioinformatics tools has become evident; for example, in the next-generation sequencing (NGS) platforms used in genetics and genomics, increases in throughput have been derived from the emergence of many informatics tools (Li and Gaquerel 2021). In the case of metabolomics, many types of analyses have been implemented, such as diversity, richness, and abundance analyses; such analyses are usually applied in community and population ecology studies and allow the identification of patterns and changes in the distribution of organisms that share the same ecological niche (Liang et al. 2020; Ni et al. 2020). In recent years, statistical tools developed for particular areas of ecology or metagenomics have been used to obtain inferences in populations with an unknown structure, in which the complexity of the sample is estimated from values of α diversity (Deng et al. 2015; Wilmanski et al. 2019). In addition, they have been successfully used to examine the metabolic structure of deep-water corals, revealing differences in metabolic footprints and metabolomic richness among coral species (Vohsen et al. 2019). Furthermore, metabolomic diversity studies have been carried out at the structural family level in the Rhamnaceae family (dicotyledonous plant family with approximately 50 genera and 900 cosmopolitan species). In this family, an analysis based on spectral library coincidence was performed, and a scalable semiautomated approach was implemented for the characterization of plant metabolites by integrating MS/MS data analysis to annotate molecular families of interest and compare their metabolic diversity between species (Kang et al. 2019). Similarly, metabolomic diversity analyses have been carried out in crop plants such as *Ipomoea batatas* (L.) Lam. (Convolvulaceae), in which 27 different cultivars were studied; the metabolic profiles of leaves and roots were obtained, and diversity was characterized based on 130 metabolites (Drapal et al. 2019) only at the chemical class level. More recently, a study was conducted on 51 species of plants belonging to the families Asteraceae, Fabaceae, and Rosaceae, which are dicotyledonous botanical families characterized by high contents of organic acids, amino acids, fatty acids, isoflavones such as genistein and catechin-type compounds and derivatives of ellagic acid. These plant species were subjected to metabolic profiling by gas chromatography time-of-flight mass spectrometry (GC–TOF–MS) and ultrahigh-performance liquid chromatography coupled to a Q Exactive™ HF Hybrid Quadrupole-Orbitrap™ mass spectrometer (UPLC-Orbitrap-MS/MS). Using leaf and stem segments, different metabolites of each species were identified depending on the analyzed organ segment, and it was concluded that the elaboration of metabolomic profiles is a useful tool aiding the rapid characterization of metabolites in plants and, thus, revealing their chemodiversity (diversity of phytochemicals delimited according to botanical taxa and/or tissues or specific phenological states) (Lee et al. 2020; Müller et al. 2020; Saito 2020). Similarly, there are many valuable studies in which high-performance metabolomic tools are used to investigate the metabolic diversity to evaluate plant diversity (Döll et al. 2021; Marr et al. 2021). However, most of these studies adopt the analysis of chemodiversity as a useful approach solely for comparisons between different conditions (experimental variables), without truly evaluating diversity and/or structural patterns based on a strict census of the phytochemical compounds that make up the metabolome per se (Shi et al. 2020; Zhao et al. 2020). Therefore, a nontargeted metabolomic dataset obtained previously as part of an integrated transcriptomics and the metabolomic project was used in this study. The aim of this work was to identify expressed genes and metabolites involved in chlorogenic acid (CGA) biosynthesis in cell suspension cultures of *Cecropia obtusifolia* Bertol (Urticaceae) grown under different conditions of nitrate deficiency-induced abiotic stress, to screen out most of their precursors and to provide evidence of their increasing biosynthesis in response to nitrate starvation (Cadena-Zamudio et al. 2020). This plant species (*C. obtusifolia*) is traditionally used in some regions of Mexico to treat diabetes mellitus type 2 because of its hypoglycemic and hypolipidemic properties, which are attributed to CGA (Nicasio et al. 2005; Cadena-Zamudio et al. 2019). The metabolomic dataset was generated using ultrahigh performance liquid chromatography coupled to a quadrupole time-of-flight mass spectrometer with an electrospray ionization source in both ionization modes (UPLC-ESI-QTOF-MS), and most of the obtained information was not fully described (Cadena-Zamudio et al. 2020). Previous studies have highlighted the importance of using metabolomic datasets obtained in both ionization modes since some metabolites are ionized (are detected) in only either positive or negative (ESI^+^ or ESI^−^) mode, and only a few are ionized in both (Cajka and Fiehns 2014; Creydt and Fischer 2017). Here, we propose a series of steps incorporating ecological tools, which are added to workflows already established in this field, to analyze metabolomic datasets. Under this methodology, some approaches, concepts, terms, and ecological-type analyses, such as the concept of “species” proposed for each of the mass-to-charge ratio and retention time (m/z_rt) pair identified in the analyses of species accumulation curves (SACs) in this study, were efficiently introduced and used in an analogous manner to many common ecology studies. Although the concept of species is not new to science, it is new to the field of metabolomics in terms of the implementation of analytical-statistical tools used in ecological and metagenomic studies. As a proposed methodology for the analysis of the dataset used here (and any other similar dataset), we suggest a systematic four-step process: (*i*) analysis of the composition of compared metabolomes based on α diversity, richness, and evenness of metabolites, (*ii*) chemometric analysis for the identification of discriminant groups in the metabolome, (*iii*) identification of differential metabolic markers using fold-changes (FCs), and finally, (*iv*) enrichment analyses and annotation of active metabolic pathways associated with differential metabolites. Thus, a global metabolic approach is implemented by using both positive and negative ionization acquisition modes, which provides a general, comprehensive, and above all impartial overview of the metabolome of *C. obtusifolia* cell suspension cultures.

## Materials and methods

### Biological material

Callus and cell suspension cultures of *C. obtusifolia* were generated based on previously reported optimized protocols (Nicasio-Torres et al. 2012). The dataset used in this study included metabolomes generated from *C. obtusifolia* suspension cells cultured over a kinetic time course in Murashige and Skoog medium supplemented with different concentrations of nitrate [27.4 mM (control group) = C27, 16 mM = C16, and 4 mM = C4], KNO_3_, and NH_4_NO_3_. The C16 and C4 concentrations of nitrate correspond to intermediate and severe nutritional stress, respectively. In addition, the kinetics were evaluated at five sampling points [T0 (3 h), T7 (7 days), T14 (14 days), T21 (21 days), and T28 (28 days)], and a total of three independent biological replicates were analyzed. For practical purposes, the nitrate concentrations will hereafter be referred to as C27, C16, and C4, and the times at which the kinetics were established will be mentioned in the following order: T0, T7, T14, T21, and T28, equivalent to 3 h, 7 days, 14 days, 21 days, and 28 days, respectively. It should be noted that even though these datasets were generated previously, the data published in our previous work were only used for the identification and quantification of CGA and its corresponding precursors in the phenylpropanoid pathway (Cadena-Zamudio et al. 2020). A deeper global analysis of the *C. obtusifolia* metabolome has not been performed before and has not been published until now.

### Obtaining methanolic extracts of *C. obtusifolia* cells cultured in suspension

Cellular pellets were harvested by filtration and centrifugation from the cultures established in flasks with 40 mL of media (Cadena-Zamudio et al. 2020). The methanolic extraction of *C. obtusifolia* cells was carried out using the ratio of the volume of HPLC-grade methanol in milliliters per gram of dry biomass obtained (1:20 m/v) over 24 h at room temperature. The samples were subsequently dried by rotary evaporation under reduced pressure (Büchi RII, Büchi), and each extract was redissolved in 10 mL of HPLC-grade methanol. Subsequently, ultrahigh-resolution liquid chromatography and high-resolution mass spectrometry (UPLC-ESI-QTOF-MS) analysis was conducted. Five hundred microliters of each solution were taken and placed in 1.5 mL tubes, and 5 μL of formic acid (final concentration of 0.1% v/v) was then added to each sample as an ionizing agent. Finally, 300 μL of each sample was placed in a 1 mL glass vial and analyzed in a Waters™ class I ultra-high performance liquid chromatograph coupled to a Waters™ Synapt G2-Si quadrupole-time-of-flight (QTOF) high-resolution mass spectrometer for the comparative analysis of the metabolic profiles. For more details, see Cadena-Zamudio et al. (2020) and Monribot-Villanueva et al. (2019). In this study, we use methanol as the extraction solvent because it has been commonly used to successfully extract polar molecules from diverse biological samples and, in some cases, it has allowed the detection of lower abundance metabolites, increasing the number of putative metabolites that can be identified (Borges et al. 2020; Dunn et al. 2011; Newton et al. 2021; Saw et al. 2021; Sitnikov et al. 2016; Yang et al. 2019).

### Untargeted metabolomic analysis using ultra-high performance liquid chromatography and accurate mass spectrometry

Chromatographic separation was carried out on an Acquity BEH column (1.7 µm, 2.1 × 50 mm), with column and sample temperatures of 40 °C and 15 °C, respectively. The mobile phase consisted of (A) water and (B) acetonitrile, both with 0.1% formic acid (SIGMA). The gradient conditions of the mobile phases were as follows: linear gradient of 1–80% B from 0–13 min, isocratic 80% B from 13–14 min, a linear gradient of 80–1% B from 14–15 min (total run time 20 min). The flow rate was 0.3 mL min^−1^, and 1 μL of each extract was injected. In positive mode, the basic chemical functional groups that are most frequently ionized include basic nitrogen (pyridinic), furans, and inorganic cations, while in negative mode, neutral nitrogen (pyrrole), carboxylic acids, and inorganic anions are preferably ionized (De Vijlder et al. 2018; Steckel and Schlosser 2019; Faixo et al. 2021). Therefore, the mass spectrometric analyses were performed in this study with the electrospray ionization source (ESI) in both negative and positive modes to achieve broader detection and compound identification in each spectrum and thus perform a more comprehensive metabolomic analysis of this plant species. The capillary voltages of the sampling cone and source compensation were 3000, 40, and 80 V, respectively. The source temperature was 100 °C, and the desolvation temperature was 20 °C. The desolvation gas flow rate was 600 L h^−1^, and the nebulizer pressure was 6.5 bars. Leucine-enkephalin was used as an internal calibrator (556.2771, [M + H]^+^, 554.2615, [M-H] ^−^). The conditions applied in the SMe analysis were as follows: mass range 50–1200 Da, function 1 with a collision energy of 6 V, function 2 with a ramp of collision energy from 10 to 30 V. The scan time was set to 0.5 s. The data were acquired and processed with MassLynx (version 4.1, Waters™) and MarkerLynx (version 4.1, Waters™) softwares according to Cadena-Zamudio et al. 2020 and Monribot-Villanueva et al. 2019.

### Analysis of the composition of compared metabolomes based on α diversity, richness, and evenness of metabolites

#### “Species” (m/z_rt) accumulation curves

After analyzing the metabolomes of the cell suspension cultures of *C. obtusifolia*, the spectrometric characteristics of the dataset were collected in a matrix; these characteristics included the relative abundance of each peak identified in both ESI modes (negative and positive) for each replicate, and the times analyzed throughout this study. Each peak represents an ion with a specific m/z value detected at a specific rt with a specific abundance (area) of the ion (counts). Therefore, each peak can be identified and defined by its m/z_rt pair. We assumed that these nonannotated and presumptive metabolites could “simulate” the species concept similarly to diversity analyses in which distinct populations (metabolomes) are compared. According to this assumption, the metabolomes were analyzed following the steps described below.

First, the previously obtained raw data was used to perform the diversity analyzes proposed in this study. These data were analyzed without any statistical treatment of the normalization type, since this would produce a statistical bias in the real values of diversity by transforming the data in such a way that they all shared the same mean value with the same deviation. However, the MarkerLynxs software was implemented to scale the matrices using the Pareto scale, which modifies the values into ranges, respecting the relationship between the range of the metabolites, the abundance, or the fold change, of a metabolite. Taking that into consideration, only the raw data (previously obtained from MarkerLynxs software) were used for these analyses. However, the assumptions of normality were tested using the Lilliefors (Kolmogorov–Smirnov) normality test for both datasets. Thereafter, we proceeded to analyze the species metabolic richness (m/z_rt) through an analysis of SACs, which is a traditional method in which the average number of species [and standard deviation (SD) in the smallest sample size] is calculated and graphed. At this point, all combinations of each sample are randomized, and the cumulative mean number of species is calculated. A curve is thus drawn between the cumulative number of species and the (sequentially ordered) sampling effort, which can be expressed in terms of the number of samples (replicates), where the slope of the curve decreases as the sampling effort increases, and the number of (new) species to be found tends toward a limit value (Ugland et al. 2003). This analysis was performed to identify common species (mz/_rt) that were usually present despite the presence of biotic or abiotic stress and uncommon species (mz/_rt) that were present only under biotic or abiotic stimuli. It was also performed to evaluate and compare the richness of the metabolomes identified in both ESI modes between the corresponding populations at each time, and the concentration of nitrate evaluated over the kinetic time-course and to estimate the maximum number of species in a community (metabolome) (Béguinot 2016; Mao et al. 2005; Thukral 2017; Ugland et al. 2003). For this purpose, the statistical software R Studio version 4.0.2 was used (Team RC 2020), as were the “vegan, BiodiversityR, permute, and lattice” packages for the analysis of ecological diversity, all of which are available in the R-project repository (cran.r-project 2020); the “random” method (which finds the expected species richness for sample size) was used, taking as “sampling points” each of the 45 biological replicates of the time-course kinetics and their richness and diversity depending on their presence or absence corresponding to ion detection. Likewise, the Simpson (DSi) and Shannon–Wiener (H′) species rarefaction curve and parametric diversity indices were calculated, and both methods were used to calculate the 95% confidence intervals of each estimator with a bootstrap value > 50 (Herrero-Jáuregui and Oesterheld 2018; Johnston and Roberts 2009; Kim et al. 2017; Odat et al. 2015). In addition, the rank abundance curves (RACs) were calculated using an abundance matrix and maximum likelihood parametric models such as the Brokenstick (null), Preemption, Log-normal, Zipf, and Zipf-Mandelbrot models (Whittaker 1965; Wilson 1991) using only the model with the lowest standard deviation and the minor Akaike information criterion (AIC), which indicates the quality of a statistical model. The RAC calculation was performed to elucidate changes at the community level in space and time, since the RACs help to identify uniformity gradients, and to demonstrate that a “biotic or abiotic” factor was uniformly shared by the species (m/z_rt) and was responsible for governing their richness and abundance (Avolio et al. 2015, 2019).

#### α diversity distribution analysis of metabolic species (m/z_rt) associated with abiotic stress

To achieve a more critical understanding of the behavior of chemical species (m/z_rt) in the metabolome of *C. obtusifolia* and how the m/z_rt population is affected as a function of time and nutritional stress (nitrate deficiency), statistical analyses of α diversity were performed, including the estimation of richness (number of species at a specific site), evenness, or uniformity [Pielou (J′), reflecting the uniform distribution of abundances among all species present at a site] and the α diversity index (H′) (Herrero-Jáuregui and Oesterheld 2018; Jost 2007; Kim et al. 2017; Moreno 2000; Thukral 2017). Additionally, parametric tests were carried out to identify statistically significant differences in relation to the α diversity observed in the metabolome (Odat et al. 2015), comparing the diversity of each population at each time and the concentrations of nitrate established over the kinetic time course, with the intention of identifying which factor determines the richness, diversity, and uniformity of species based on the metabolome. For this purpose, one-way analysis of variance (ANOVA), *F* value calculation (to determine if the variability between the means of the groups is greater than the variability of the observations within the groups), and Tukey’s comparative method (to generate confidence intervals of the differences identified between the means of the factor levels, with an error rate ≤ to 0.05, equivalent to a 95% confidence level) were implemented in the analysis. Pearson correlation coefficient (r) analysis was also performed (to evaluate the linear relationship between two continuous variables using a presence-absence matrix) for each of the times established in the time-course kinetics, in which comparisons with the control concentration (27.4 mM *vs.* 16 and 4 mM) along with the sampling times were performed. The coefficient of determination (R^2^) was also calculated to identify the percentage of the variation in the response explained by the linear model, and statistical probability values (*p* values) were calculated to differentiate and identify those results (variables) that were statistically significant or different from each other; here, one-way ANOVA and the corresponding *F* value were applied to determine if the variability between the means of the groups was greater than the variability of the observations within the groups. It should be noted that all of these diversity analyses were performed using raw data (i.e., without implementing any kind of statistical transformation). This approach was adopted because transformations mostly involve the implementation of Euclidean distances in their ordinations (e.g., the Hellinger transformation), and as such, they are appropriate for performing an analysis that requires variables linearly related to each other on the same scale (e.g., principal component analysis (PCA) or redundancy analysis (RDA)). However, these types of transformations are not appropriate for count data (presence–absence), such as those used in the analyses of SACs or the subsequent analyses of diversity (indices) carried out in this study (Legendre and Gallagher 2001). Therefore, distances that are complements of similarity coefficients for binary data (0–1), such as simple coincidence coefficients, SACs, RACs, or H′, DSi, Jaccard, or Sorensen indices, cannot be obtained through such transformations because they are not Euclidean distances (Gower and Legendre 1986; Oksanen et al. 2007). To carry out these analyses, the statistical software R version 4.0.2 was used (Team RC 2020), as were the “vegan, ggplot2, Viridis, BiodiversityR and corrplot” packages, which are available in the R-project repository (cran.r-project 2020).

### Chemometric analysis for the identification of discriminant groups in the metabolome

#### Raw data processing

The raw data obtained by mass spectrometry were processed with MassLynx and MarkerLynx (both version 4.1, Waters™) software. Thereafter, MetaboAnalyst 4.0 software (Chong et al. 2019; Xia et al. 2009) was used to perform data normalization to enhance identification characteristics such as grouping, classification, and dimensional reduction and minimize both induced and total noninduced variation, to emphasize biologically relevant information while eliminating noise or noninformative variables, baseline noise, and variables that showed low reproducibility in the dataset (van den Berg et al. 2006). In ESI^+^ mode, the nontransformed mean values and autoscaling method (where the responses of each variable are centered by subtracting its mean value, followed by division by the corresponding standard deviation) were used. In ESI^−^ mode, the cube root transformation and the range scaling method (centered on the mean and divided by the range of each variable) were used. Each of the methods was implemented independently for each ionization mode until a Gaussian data distribution was obtained (or “bell-shaped”) based on box plots, where a maximum of 50 features were shown due to space limitations in the graph, and kernel density plots, which showed the general behavior of the data based on the comparison of all samples before and after the normalization process (Supplementary Fig. S1) (Bartel et al. 2013).

### Analysis for the identification of statistically relevant binary classes

Based on previously normalized data, unsupervised multivariate analyses were performed. For example, PCA allowed binary class discriminations (Worley and Powers 2016; Bartel et al. 2013; Li et al. 2018) using only five components determined via the elbow plot method, which were subsequently plotted in pairwise score plots to provide an overview of the different patterns of separation between the PCs explaining the greatest percentage of variation. This approach was applied to visualize the multidimensional relationships of the measured variables (metabolites) in predefined states (concentrations-times) (Worley and Powers 2013) and to identify trends within the dataset and the experimental groups (discriminant groups) responsible for the highest percentage of variance within the experiment to reduce dimensionality (variables); thus, the separation of groups and their classifications (I-III and I-IV for positive and negative ionization modes, respectively) were achieved. Pairwise comparisons of C27 at early times *vs.* C16 and C4 at late times were performed for both ionization modes, generating paired comparisons between the control concentration and the stress concentrations at early times *vs.* late times. MetaboAnalyst 4.0 software was used as the analysis tool (Chong et al. 2019; Feng et al. 2019; Wang et al. 2020). Furthermore, hierarchical clustering heatmaps were generated as a visualization tool to represent the relative abundance of m/z_rt pairs detected in each sample based on color intensity (Wilkinson and Friendly 2009). Additionally, a Pearson correlation coefficient analysis was implemented, in which comparisons of m/z_rt *vs.* m/z_rt and group (sample) *vs.* m/z_rt were performed to identify the time or nitrate concentration at which the m/z_rt with the greatest abundance occurred and, thus, elucidate the corresponding distribution patterns based on the conditions established over the kinetic time course. The statistical software R Studio version 4.0.2, available in the R project repository (Benton et al. 2015; cran.r-project 2020; Team RC 2020), was used for this purpose.

### Identification of biomarkers via paired statistical analysis based on rates of change (fold-changes)

The groups identified as discriminant by PCA were used to perform an FC analysis via paired comparisons, with the aim of identifying overaccumulating m/z_rt pairs that were significantly different between distinct conditions (discriminant groups), with an established threshold of FC (≥ 2) (Funk et al. 2020). The results were visualized in volcano plots to identify the differential metabolites of interest using MetaboAnalyst 4.0 software (Chong et al. 2019). Each comparison was performed *versus* the corresponding control condition (C27) (for those in which the compared group was different from the control). In ESI^−^ mode, when discriminant groups corresponding to the control condition, the paired comparisons of these groups *versus* both stress conditions (16 and 4 mM) were also performed. Subsequently, bar graphs of the m/z_rt pairs identified as differential in the FC analyses were generated to quantify the total number of differential m/z_rt pairs by comparison. Then, frequency graphs (histograms) were produced to identify which m/z_rt pairs were found more than once across the comparison performed. Redundant m/z_rt pairs were removed to obtain the real number of differential m/z_rt pairs by ionization mode. R Studio software version 4.0.2 and the ggplot2 package (cran.r-project 2020; Team RC 2020) were used for that purpose. Hereafter, differential m/z_rt pairs are referred to as dMEs.

### Metabolic pathways enriched by dMEs and their putative annotations

Once the dMEs were obtained from the *C. obtusifolia* metabolome, their annotation and the identification of metabolic pathways enriched by the dMEs were carried out. Python 3.8 and Mummichog 2.3 software were used (Li et al. 2013; Python Software Foundation 2020; Mummichog 2020) to obtain the nomenclature of codes from the Kyoto Encyclopedia of Genes and Genomes (KEGG; Kanehisa et al. 2016) for each dME in both ionization modes (see, for example, Barranco-Altirriba et al. 2021; Walker et al. 2021). *Arabidopsis thaliana* (L.) Heynh. (Brassicaceae) was selected as the reference organism for the identification of the KEGG codes of metabolic pathways, and Mummichog was used as the prediction algorithm; the algorithm included all paired metabolites in a reference metabolic network (*A. thaliana*), taking the m/z of each dME as the input. The confidence scores (edge weights in the network) were supported by the FC values of the dMEs. This clustering process refined the annotation of empirical compounds (ECs), significantly reducing false-positive annotations by mapping all possible matches in the network, emphasizing local enrichment. The above description gave rise to the first step in the tentative identification of m/z_rt pairs, which will be called tentative metabolites (tMEs) hereafter (Li et al. 2013). Following the assignment of this tentative identity (putative annotation), the Pathos web facility (http://motif.gla.ac.uk/Pathos/; Leader et al. 2011; Creek and Barrett 2014) was used to identify the presence of tMEs within each of its active metabolic pathways and thereby perform a theoretical reconstruction of them from the previously identified tMEs. To visualize these pathway enrichment results, a Circos plot in which each tMEs was mapped against its corresponding metabolic pathway was generated. R Studio version 4.0.2 statistical software and the “patchwork, hrbrthemes, circlize, and chorddiag” packages were used for that purpose (cran.r-project 2020; Team RC 2020). Finally, to provide a greater level of confidence in the annotation process conducted by using Mummichog and Pathos, a match search was performed between the chemical formulas of the tMEs and their consistency with experimentally obtained rt and m/z pairs or those reported in the PubChem (Kim et al. 2020) and FoodDB databases (https://foodb.ca/; FoodDB 2020). The search was limited to plants with a mass accuracy range of + / − 5 ppm, which confirms the identity of the tMEs at level 3 according to several previous reports (Chaleckis et al. 2019; Fernie et al. 2011).

## Results

### Metabolic profiles, spectrometric data, and their obtainment

The comprehensive mass spectrum library (metabolic profiles) of *C. obtusifolia* included metabolites obtained in both ESI^−^ and ESI^+^ and was generated on a high-resolution mass spectrometry platform with the objectives of avoiding biases favoring erroneous conclusions and achieving greater coverage of the metabolome, which allowed us to understand and analyze the phenotype and its metabolic plasticity in greater detail. From all samples analyzed, a total of 5331 peaks (spectrometric features) were obtained. Each peak represented an ion with a specific m/z_rt and a specific abundance of the ion (counts). All of the unidentified and presumptive metabolites were analyzed according to the steps described below.

### Analysis of m/z_rt data pairs (metabolic species)

The metabolic species were collected from the mass spectra obtained in both ESI modes (raw data) (Supplementary Tables S1 and S2). To avoid inducing bias in the estimation of m/z_rt richness and diversity among the samples analyzed to obtain the *C. obtusifolia* metabolome, the generated matrix did not receive any statistical treatment. However, when the assumptions of normality were tested using the Lilliefors (Kolmogorov–Smirnov) normality test, both datasets were observed to behave normally, showing *p* values = 0.4418 and 0.4948 in ESI^+^ and ESI^−^ modes, respectively. These results indicated that the other parametric statistical tests could be carried out.

**Fig. 1:**
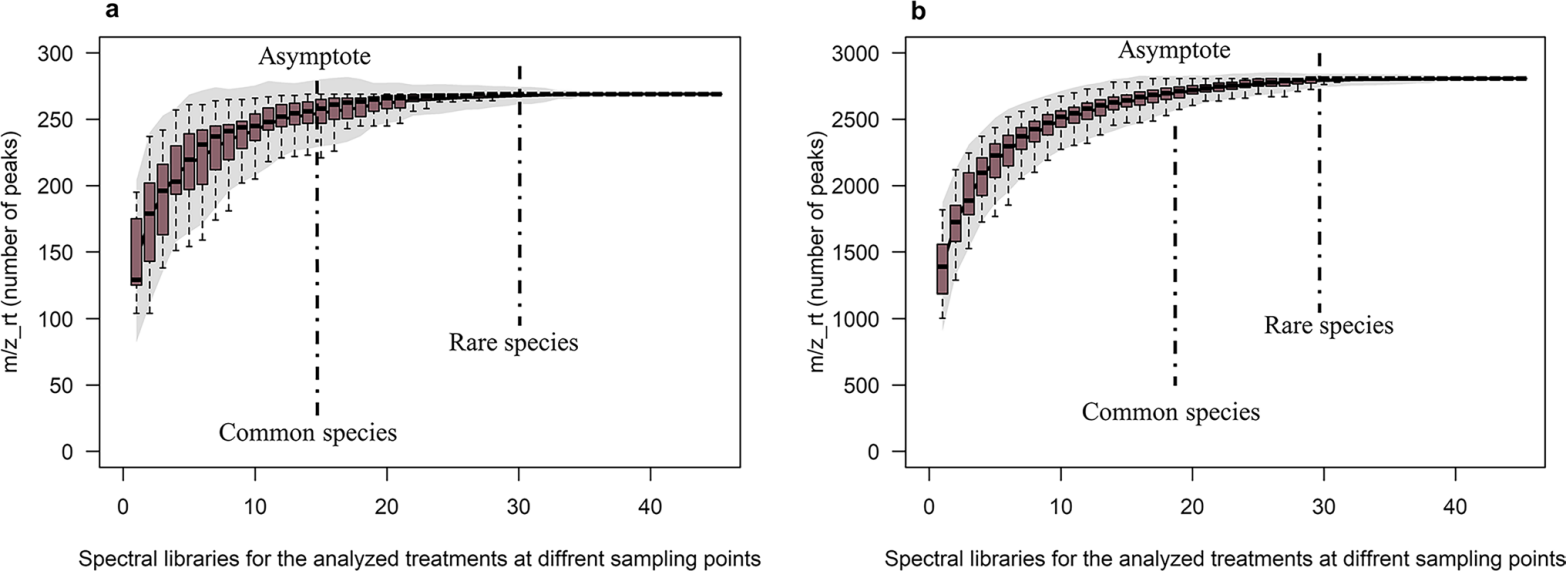
Species accumulation curve (SAC) analysis of the metabolome of *C. obtusifolia* in both ESI modes. **a** The SAC analysis in ESI^+^ mode is shown, revealing a cumulative richness of 269 species or m/z_rt pairs (± standard deviation SD, with the 95% confidence interval [shades of gray]), where the maximum peak of incidence of an m/z_rt pair belongs to sample C27_T14 (195 m/z_rt pairs), which is equal to the asymptote. Note that samples with common m/z_rt pairs correspond mainly to the C27 and C16 treatments (medium containing 27.4 mM or 16 mM nitrate), while rare m/z_rt pairs are identified at later times (T21 and T28) corresponding to the 4 mM nitrate treatment (C4). **b** The SAC for ESI^−^ mode yields an estimated m/z_rt richness of 2807 (± SD values, with its 95% confidence interval [shades of gray]), where the highest m/z_rt incidence (asymptote) is found for sample C27_T28 (1818 m/z_rt pairs), and the samples with the lowest m/z_rt richness are C4 at T0, T14, T21, T28, and C16 at T0 (1267, 1228, 1099 and 1003 m/z_rt, respectively)

Using the Chao1, Jack 1 and Jack 2 indices as parametric estimators, the m/z_rt composition of the *C. obtusifolia* metabolome was first analyzed using SACs (see “Materials and methods” for more details). A total of 269 and 2807 metabolic species (m/z_rt) were obtained in ESI^+^ and ESI^−^ modes, which represent the calculation and graph of the average number of species (and the SD of the smallest sample size), respectively. The maximum accumulation of metabolic species was observed under the control condition (nitrate concentration of 27.4 mM) at sampling point T14 in ESI^+^ mode. Once the SACs reached the asymptote, the curves comprised a total of 195 species (72.4% of the total m/z_rt pairs identified from the mass spectra generated in ESI^+^ mode). The metabolomes with the fewest species (uncommon m/z_rt) were identified at a concentration of 4 mM at T14, T21, and T28 and showed a total of 115, 105, and 104 m/z_rt pairs, respectively (Fig. 1A). The above findings demonstrated that the ESI^+^ SACs initially presented a high proportion of relatively abundant species, with an initial steep ascending slope and an early plateau, indicating that uncommon species were the least detectable in this ionization mode.

Regarding the ESI^−^ mode, the T28 sample obtained under the optimal nitrate concentration (C27) was identified as the most chemically complex sample with the greatest number of species [a total of 1818 m/z_rt values (asymptote)]. In addition, the samples with the lowest richness or diversity of m/z_rt pairs corresponded to the severe nitrate starvation treatment (4 mM) at T0, T14, T28, and T21 (1267; 1228; 1099; and 1003 m/z_rt pairs, respectively), followed by the 16 mM treatment at T0 (1,146 m/z_rt pairs) (Fig. 1B) (Supplementary Table S3). In this ionization mode, the behavior of the SACs differed from the behavior found in ESI^+^ mode, showing a moderately high rate of common species and a relatively high representation of rare species. In other words, the ESI^−^ mode seemed to favor the detection of m/z_rt pairs that were common or identifiable in many of the samples and, to a lesser extent, those identified as uncommon m/z_rt pairs; thus, a greater spectrum of identifiable species was found within the metabolome in negative ionization mode.

Based on the results described above, it is possible to conclude that both ESI modes represent the *C. obtusifolia* metabolome but not equally so. The two ionization modes show certain differences, such as a greater or smaller number of rare species, indicating a clear dominance of various m/z_rt pairs in ESI^+^ but not in ESI^−^. Therefore, discriminating between the modes or including only one of these ESI modes in untargeted metabolomic studies not only restricts the amount of m/z_rt pairs that can be identified but could also generate a bias in the conclusions obtained based on these results. Here, the metabolome represented by the m/z_rt data pairs obtained from both ESI^+^ and ESI^−^ modes showed high DSi and H′ values (see “Materials and methods” for more details), ranging from 3.1 to 4.4 and 3.2 to 6.0 for DSi and H′ (α diversity), respectively, in each analyzed ionization mode (Supplementary Table S4). These results indicate that there is a clear decrease in m/z_rt richness and diversity as a function of nutrient availability over time. Thus, it can be concluded that m/z_rt diversity and richness depend on nitrate deprivation and that over time, this abiotic stress favors the biosynthesis of “rare” m/z_rt pairs at later times rather than favoring the biosynthesis of “common” m/z_rt pairs, unless the latter are influenced or respond to the type of stress analyzed.

To verify whether abiotic stress governs the diversity of m/z_rt pairs in the metabolome of *C. obtusifolia*, rank abundance curve (RAC) analyses were performed for both ionization modes. The results obtained showed that in both ESI modes, the richness and accumulation of species conformed to the null model as time passed during the kinetic time course, raising the question of how many of the patterns observed in the metabolome may be due to pure chance. In this case, the answer was none, as this model expressed the biological distribution of m/z_rt pairs and indicated that an important factor (abiotic stress) was shared uniformly by m/z_rt pairs and that it governed their richness and abundance (Supplementary Fig. S2).

### Nitrate starvation as a modulator of the α diversity, richness, and evenness of m/z_rt pairs in the metabolome of *C. obtusifolia*

**Fig. 2:**
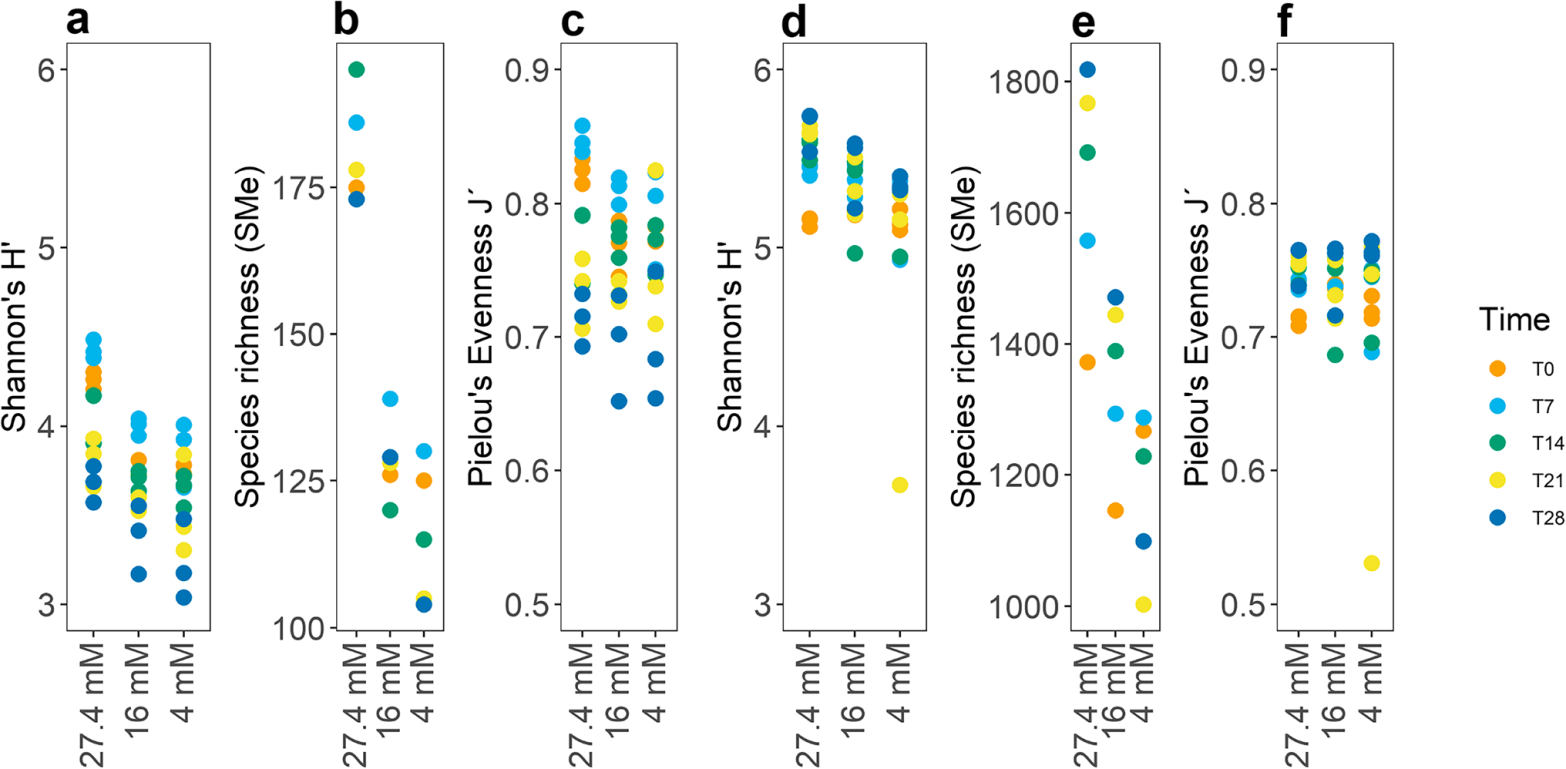
Analyses of m/z_rt α diversity, richness, and evenness in the *C. obtusifolia* metabolome. **a** α diversity at different nitrate concentrations, where C27 is the treatment with the highest diversity observed in ESI^+^ mode. **b** Species richness in ESI^+^ mode, where the optimal nitrate concentration is again the one with the highest values. **c** Pielou evenness in ESI^+^ mode, where 0 and 7 days are the most uniform time points in C27, and 7 days are the time point with the highest J ′index in C16 and C4. **d** Shannon’s diversity in ESI^−^ mode, where the optimal nitrate concentration is again associated with the greatest m/z_rt diversity. **e** In relation to the richness of species of ESI^−^ mode, T21, T28, and T14 are the time points at the optimal concentration with the highest richness relative to all time points at the stress concentrations. **f** Regarding the evenness of species observed in ESI^−^ mode, there was no clear m/z_rt domain since a very similar structure was found all concentrations and times in ESI^−^ mode

To obtain a better understanding of how the numbers of m/z_rt values were modulated within the analyzed samples conforming to the metabolome of the *C. obtusifolia* cell suspension cultures, α diversity analyses were performed using the parameters H′, species (m/z_rt) richness, and Pielou (J′) evenness or uniformity for the datasets obtained in both ionization modes. The obtained results showed that in both ESI modes (Fig. 2A, D), the α diversity (Shannon’s H′) of the metabolome decreased in relation to the increase in stress induced by nitrate starvation. This was especially notable for those m/z_rt pairs identified in ESI^+^ mode (Fig. 2A), where the nitrate starvation treatments (C16 and C4) resulted in H′ values slightly higher than 4 but below the values observed in ESI^−^ mode (Fig. 2D), where the H′ values ranged between 4.9 and 5.6; thus, the results indicated that a more diverse “community” (sample) existed in the samples that were less affected by nitrate starvation. Concerning the difference in m/z_rt richness between the two ESI modes (Fig. 2B, E), m/z_rt richness was severely affected in the ESI^+^ mode (Fig. 2B). When nitrate was reduced in the medium in C16 and C4, the richness decreased by 28.2% and 37.3%, respectively, showing the greatest effect at T14, T21, and T28, respectively. A decrease in m/z_rt richness was also observed in ESI^−^ mode (Fig. 2E); however, it was not as drastic as tat in ESI^+^ mode, with a decrease of only 18.8% in the C16 treatment and 29% in the C4 treatment, both of which are considered to represent stress conditions due to nitrate starvation.

In the case of the J′ index in ESI^+^ mode (Fig. 2C), it was observed that at the T0 and T7 sampling times, the control concentration (C27) presented the highest values, close to 1, which indicated that most m/z_rt pairs were equal or equally abundant. At T14, T21, and T28, the opposite pattern was observed, in which the evenness of m/z_rt pairs was lower, suggesting a dominance of m/z_rt over the other sampling points. In relation to the nitrate starvation treatments (C16 and C4, respectively), very similar behavior was observed, where the least uniform time was T28, and the most uniform was T7 for both concentrations. The results in ESI^−^ mode (Fig. 2F) showed opposite behavior to those in ESI^+^ mode, displaying a *quasi*-constant uniformity in the distribution of m/z_rt pairs over time, subject to the established nitrate concentrations during the sampling points, despite the concentration of 4 mM showing a fairly low J′ index. The community generally showed uniform, conserved behavior with values of 0.69 and higher for 98% of the samples; thus, the *H*′ value was high, demonstrating that the identified structures in the metabolic communities were organized differently between the two ESI modes, regardless of whether the biological stimuli were the same. These findings demonstrate the importance of implementing global metabolic analyses in both ionization modes because considering the results together makes it possible to identify the metabolic fluctuations of *C. obtusifolia* under abiotic stress over time.

**Fig. 3:**
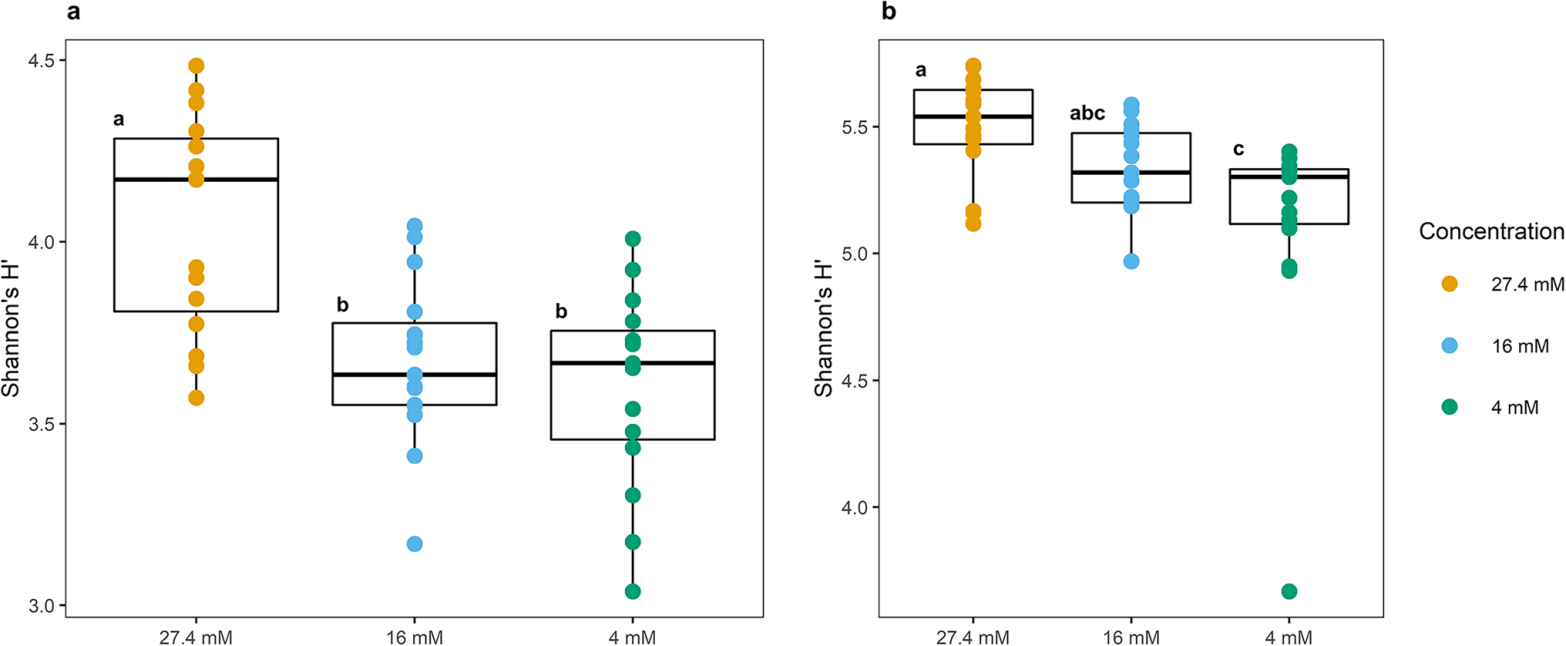
ANOVA to detect statistically significant differences between metabolic populations at different nitrate concentrations in ESI^+/−^ modes. **a** ANOVA of the ESI^+^ mode results, in which the population corresponding to the control condition (C27) differs from those in nitrate-limited conditions (C16 and C4). **b** ANOVA of the ESI^−^ mode results, in which the populations recorded under the stress conditions (C16 and C4) are different from the population under optimal nutrient conditions (C27)

In addition, Tukey’s grouping and variance analyses (one-way ANOVA) were performed to identify significant differences in the H′ values obtained in the diversity analysis over time and between the different concentrations of nitrates established during the sampling time course. In the ESI^+^ mode analysis performed at the optimal nitrate concentration (C27), the number of species identified was significantly different from those recorded under reduced nitrate concentrations (C16 and C4) (*p* value ≤ 0.05, one-way ANOVA; *F* = 12.16) (Fig. 3A). In ESI^−^ mode (Fig. 3B), the statistical analyses indicated that there were also statistically significant differences in metabolic diversity between the three nitrate concentrations, where the C4 concentration showed the greatest differences and presented the lowest diversity in this ionization mode (*p* value ≤ 0.05, one-way ANOVA; *F* = 6.064).

**Fig. 4:**
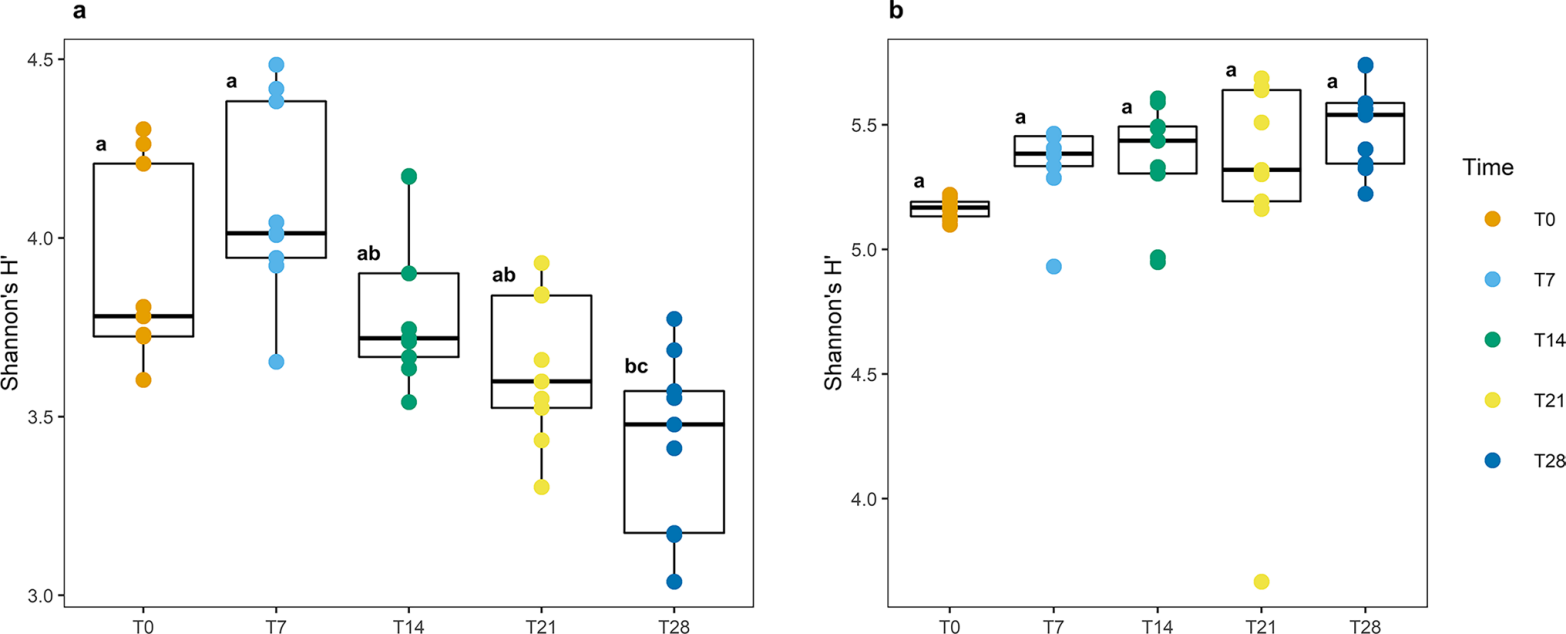
ANOVA to detect statistically significant differences between metabolic populations over time in both ESI modes. **a** ANOVA of the ESI^+^ mode results, showing that the metabolic populations of early time points differ from those of late time points. **b** ANOVA of the ESI^−^ mode results, in which no statistically significant differences are observed between the metabolic populations over time

The same statistical analyses were performed among the times established in the kinetic analysis. In ESI^+^ mode (Fig. 4A), it was found that the results for the early time points (T0 and T7) were similar to each other but differed from those obtained at the other sampling times (T14, T21, and T28), where the latest time showed the greatest difference because it presented the lowest metabolic diversity among the analyzed times; thus, T28 was the most affected point in this category (*p* value ≤ 0.05, one-way ANOVA; *F* = 9.576). In ESI^−^ (Fig. 4B), no statistically significant differences in diversity were found (*p* value = 0.25, one-way ANOVA; *F* = 1.406). These findings reinforce the findings obtained in the J′ index analysis (Fig. 2F), supporting the concept that a more uniform community will vary less in terms of the proportion of m/z_rt pairs.

According to the results described thus far, it can be stated that the metabolic species (m/z_rt) detected in ESI^+^ mode have a greater tendency to promote the biogenesis of rare species (m/z_rt) that are benefited by nitrogen stress over time, thus revealing a less diverse and uniform metabolic community than that found in ESI^−^ mode. This was revealed by the comparisons between the two ionization modes, which led to the identification of different patterns and trends between the two modes. However, it is undeniable that the implementation of both positive and negative modes offers a more comprehensive view of the metabolome than is possible when a single polarity is used, since the global metabolomic approach implemented in this work allowed us to achieve a quantitative and unbiased analysis of the metabolome. Using an extended mass spectrometry ionization approach (ESI^+/^^−^) enhanced the ability to detect more metabolites, resulting in an increase in the number of ionic species detected, which may translate into greater coverage of the metabolome of *C. obtusifolia*. These findings demonstrate the clear advantage of this strategy, since the study of the complementary information obtained in the two ionization modes was very useful for confirmation purposes; it both significantly increased selectivity and minimized the probability of obtaining false positives and negatives. In addition, it prevented the formulation of erroneous conclusions that were not necessarily related to the studied phenomenon but to effects on the data resulting from technical alterations, such as changes in the ESI mode used.

### Identification of correlation patterns between nitrate concentrations over time

**Fig. 5:**
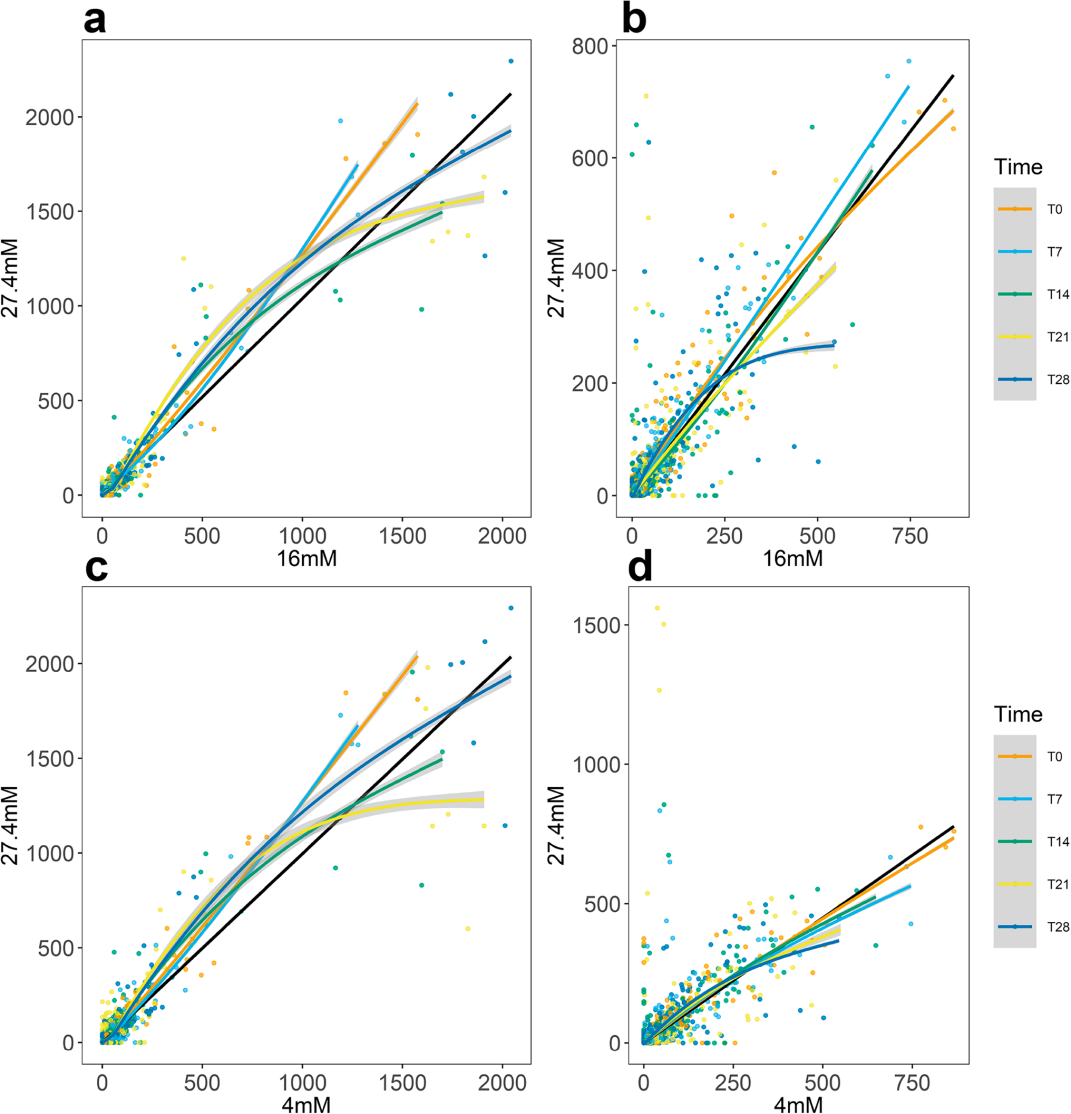
Correlation models and analysis of covariance. The trend models and adjusted linear model of the Pearson correlation coefficient generated for the concentrations of **a** 16 mM and **c** 4 mM in ESI^+^ mode are shown. Similar analyses are shown for the data obtained at **b** 16 mM and **d** 4 mM in ESI^−^ mode

Pearson correlation coefficient (r) and covariance (R^2^) analyses were carried out sequentially to determine the extent of the relationships and variation of the detectable m/z_rt pairs present in the control samples (C27) compared with those under nitrate deprivation (C16) over time. In ESI^+^ mode (Fig. 5A), positive *r* and *R*^2^ values were found at both concentrations at all times (*T0* = *r* = 0.95; *R*^2^ = 90.9%; *T7* = *r* = 0.96; *R*^2^ = 93.9%, *T14* = *r* = 0.94; *R*^2^ = 90%; *T21* = *r* = 0.94; *R*^2^ = 89.7%, and *T28* = *r* = 0.96; *R*^2^ = 93.1%), where T28 showed the highest correlation and variance values, displaying a trend line that was better adjusted to a linear model, where all values were statistically significant (*p* value ≤ 0.05, one-way ANOVA). When the same comparison was performed for the dataset obtained in ESI^−^ mode (Fig. 5B), it was observed that early times, such as T0, T7, and T14, presented the highest correlation values (*T0* = *r* = 0.95; *R*^2^ = 91%; *T7* = *r* = 0.96; *R*^2^ = 92.9%; *T14* = *r* = 0.79; *R*^2^ = 63.9%) and were better adjusted to the linear model. Later times, such as T21 and T28, yielded values just above 0.5 relative to early times (*T21* = *r* = 0.77; *R*^2^ = 60.1%; *T28* = *r* = 0.76; *R*^2^ = 58.5%), since the linear trend was far from the adjusted model. Nevertheless, all trend lines importantly showed a *p* value ≤ 0.05 (one-way ANOVA).

At the C4 concentration, the ESI^+^ mode analysis (Fig. 5C) showed behavior almost identical to the homologous condition C16 was observed (*T0* = *r* = 0.97; *R*^2^ = 95.4%; *T7* = *r* = 0.96; *R*^2^ = 93.8%; *T14* = *r* = 0.93; *R*^2^ = 87.9%; *T21* = *r* = 0.88; *R*^2^ = 78.7%; *T28* = *r* = 0.95; *R*^2^ = 92%), with a *p* value ≤ 0.05 (one-way ANOVA) for all models. Finally, the ESI^−^ mode dataset for the same concentration (C4) were also compared against C27 (Fig. 5D), and a clear decrease in the size of the trend lines was observed; the model best fit time T0 (*r* = 0.91; *R*^2^ = 84.2%), as the other models corresponding to times T7, T14, T21, and T28 showed a clear decline in their trend lines, moving away from the adjusted linear model and impacting the obtained correlation and covariance values (*T7* = *r* = 0.81; *R*^2^ = 66.8%; *T14* = *r* = 0.80; *R*^2^ = 64.2%; *T21* = *r* = 0.52; *R*^2^ = 27.7%; *T28* = *r* = 0.83; *R*^2^ = 70.3%). It was shown that the direction of the relationship between the variables decreased over time, showing a tendency toward negativity, at least in the later times at the highest stress concentration (C4); thus, the correlation between the variable nitrates and time will cease to make biological sense after a long time of exposure of *C. obtusifolia* cell suspension cultures to a limiting nutrient concentration.

Previous models have explained the way in which plants respond to various types of stress according to four phases described by Lichtenthaler (Lichtenthaler 1998): *(i)* a response phase or alarm phase (reduced normal function, decreased vitality); *(ii)* a restitution phase or resistance phase (adaptation and repair processes are activated) (these two phases were observed in the early stages of the kinetic time course at T0 and T7 under moderate loads of biological stress, such as that imposed by C16; Fig. 5A, B); *(iii)* a final phase or stage of exhaustion (high intensity of stress, overloaded adaptive capacity, chronic illness or death) (phases *ii* and *iii* were both observed at all late time points (T14, T21 and T28) under the highest stress condition (C4); Fig. 5C, D); and *(iv)* a regeneration phase (total partial regeneration of physiological function when the stress factor is removed and the damage is not severe) (this phase was not observable in the present work due to the times set in the kinetic analysis. However, the stress response model proposed by Lichtenthaler was applicable because of the diversity analyses (Fig. 2) and the correlation models adopted in this study (Fig. 5). Therefore, the correlation analyses showed decreasing tendencies in relation to time, and the diversity analyses interpreted these tendencies as decreases in the diversity, richness, and evenness of species, which translates into the induction and/or biosynthesis of metabolic species (m/z_rt) closely related to the maintenance of cellular homeostasis, allowing increased biosynthesis of metabolites that contribute to coping with stress (rare species) and then allocating nutritional resources to the functioning of secondary metabolism, thus compromising growth to ensure survival. This induces phenotypic-metabolic plasticity so that the plant can perceive endogenous stimuli and undergo adaptations during its development that reflect changes in its physiology and metabolic activity.

### Chemometric analysis for the identification of discriminant groups in the metabolome of *C. obtusifolia*

**Fig. 6:**
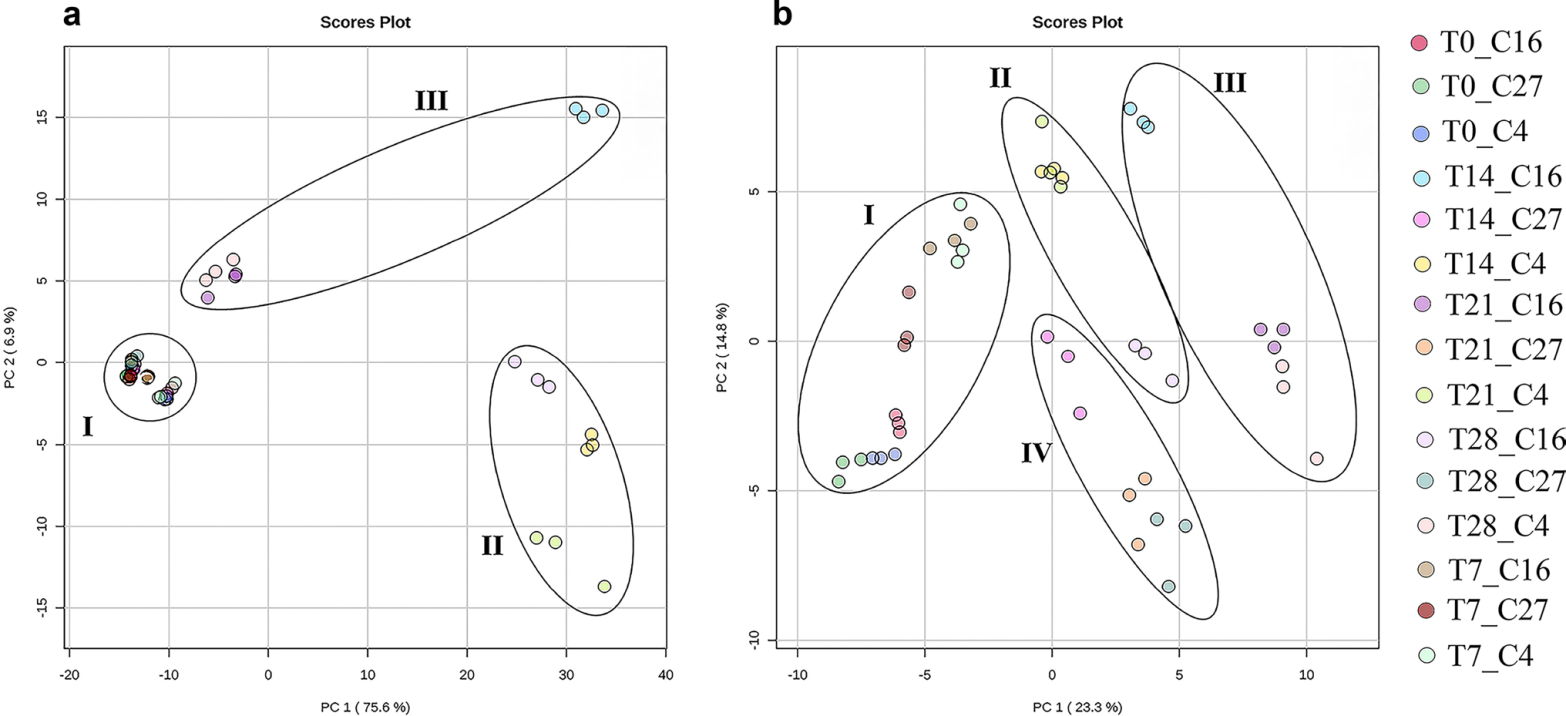
2D PCA for the identification of discriminant groups. **a** Three uncorrelated groups were identified in ESI^+^ mode, where group I corresponded to the control condition (at all times) and groups II and III to the stress conditions (C16 and C4) at late times (T14, T21, and T28). **b** Four uncorrelated groups were identified in ESI^−^ mode, where group I corresponded to the early times of T0 and T7 (in all concentrations), groups II and II had the same composition indicated by the ESI^+^ results, and group IV was the only group confirmed at late time points (T14, T21, and T28) at the control concentration (C27)

To identify the groups of discriminant samples that explained the highest percentage of variance in this study, unsupervised statistical analyses were performed. For this purpose, the raw data matrix previously obtained by accurate mass spectrometry was subjected to statistical normalization, and the analysis was carried out for the data from both ESI modes until a bell-shaped plot was obtained and adjusted to a normal distribution (Supplementary Fig. S1). Once this process was completed, a two-dimensional exploratory analysis of principal components (PCA 2D) was carried out; 75.1 and 7% of the observed variance were explained by the first two components in ESI^+^ mode (Fig. 6A and Supplementary Fig. S3a), whereas 23.3 and 14.8% of the variance were explained by these components in ESI^−^ mode (Fig. 6B and Supplementary Fig. S3b). Subsequently, 2D PCAs were carried out to graphically visualize the samples that explained the highest percentage of variance, obtaining three groups for the ESI^+^ mode data. Group I corresponded to T0 and T7 at concentrations C27, 16, and 4, while group II corresponded to T14_C4, T14_C16, T21_C4, and T28_C4 and group III to T21_C16 and T28_C16. (Fig. 6A). In the ESI^−^ mode data, four groups were identified (Fig. 6B): group I corresponded to the early times of T0 and T7 (in all concentrations), group II to T21_C4, T14_C4, and T28_C16 and group III to T14_C16, T21_C16, and T28_C4, as observed in ESI^+^ mode. However, in the ESI^−^ data, an additional group IV was identified, which consisted of the samples from the later times (T14, T21, and T28) at the control concentration (C27). In addition, a PCA biplot was obtained for both ionization modes to evaluate the statistical importance of each variable (vector) in every component and its influence on the component from the evaluated time onward (Supplementary Table S5, S6, and Supplementary Fig. S4); the results corroborated the designation of clusters by 2D PCA. These results allowed us to conclude that with few exceptions, the groups identified corresponded to the different concentrations of nitrates at later times and could be considered uncorrelated groups in both ESI^+/^^−^ modes (Fig. 6). In both ionization modes, T0 and T7 (ESI^+^ mode at all nitrate concentrations) showed no difference as a function of the nitrate concentration (Fig. 6). However, in ESI^−^ mode, the identification of a fourth group corresponding to the control condition at later times indicated that although the *C. obtusifolia* cell suspension cultures were in an “optimal nutritional condition,” prolonged exposure (> 7 days) could alter metabolic levels, as shown by 2D PCA (Fig. 6B). This permitted a reduction in the possible samples available for the future identification of discriminant chemical markers through the pairwise comparison of the control *versus* stress conditions (I *versus* II; I *vs.* III; I *vs.* IV).

Once the discriminant groups were obtained by 2D PCA, heatmap analyses were carried out; the first of these analyses compared m/z_rt *vs.* m/z_rt (metabolite *vs.* metabolite) correlation patterns, after which a presence-abundance analysis was performed to identify if the highly correlated m/z_rt pairs corresponding to the groups identified as discriminant by 2D PCA. A very clear, exclusive pattern of m/z_rt pairs with correlation values close to 1 was indicated by the ESI^+^ mode data (Supplementary Table S7). The ESI^−^ mode data revealed even more patterns with correlation levels close to 1 (Supplementary Table S8) and showed less exclusive correlative patterns than the ESI^+^ mode data, confirming the findings of the diversity analyses (J′).

**Fig. 7:**
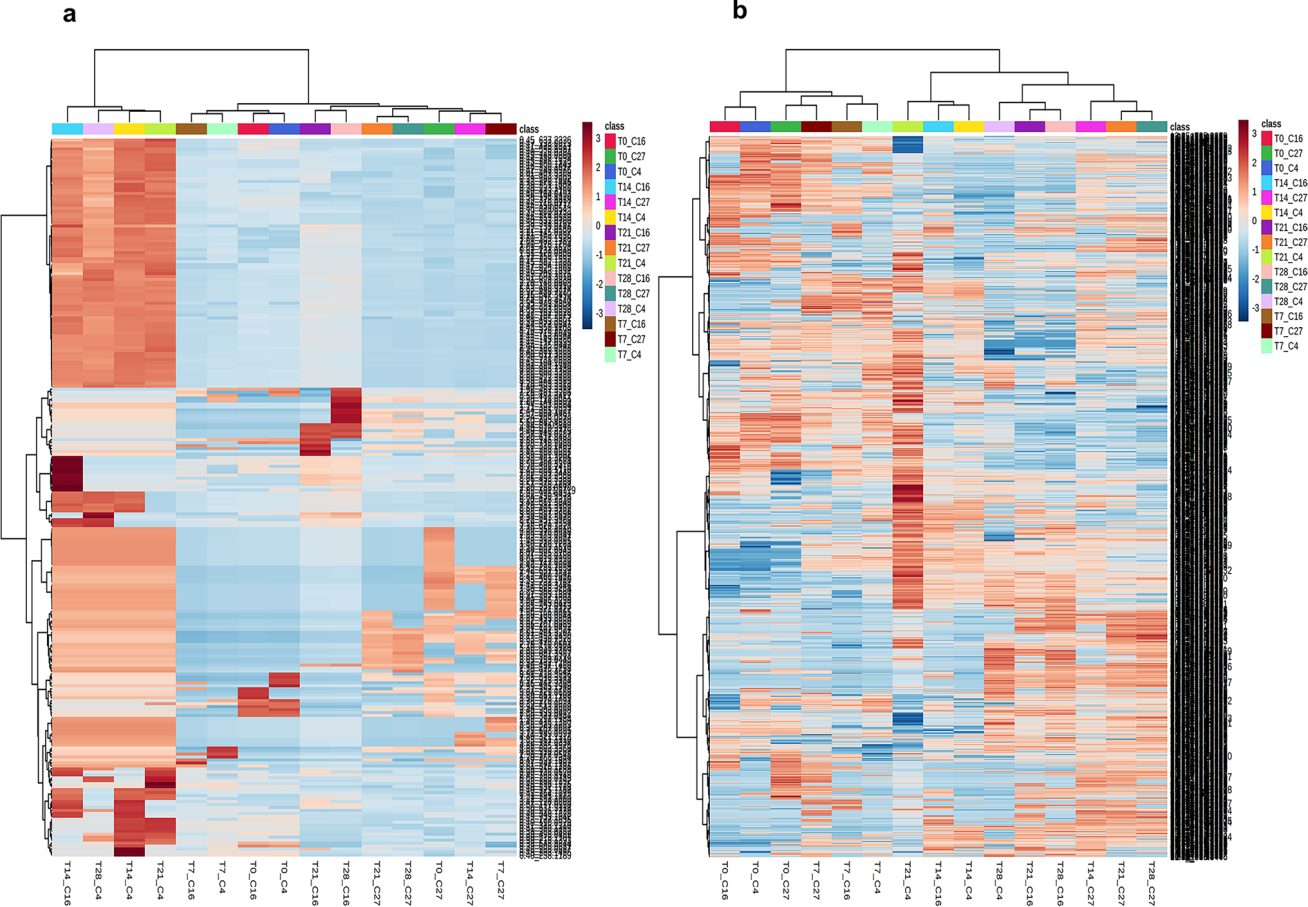
Hierarchical clustering heatmap of the relative m/z_rt abundances identified in the comparisons of all treatments. **a** Heatmap of ESI^+^ mode data in which clear patterns of m/z_rt pairs with high relative abundance values (values greater than or equal to 2) and the corresponding sampling groups are observed, highlighting all of the groups identified as discriminant by 2D PCA. **b** Heatmap of ESI^−^ mode data in which the relative abundance values (≥ 2) again show all groups identified as discriminant by 2D PCA

The previously obtained correlation patterns were confirmed by again using heatmaps to obtain an analytical representation of the relative abundances of each m/z_rt pair against each treatment analyzed under distinctive nitrate concentrations (C27, C16, and C4) and times (T0, T7, T14, T21, and T28). Considering that the relative abundance values corresponded to the total counts of individual ions in the mass spectra; once these values were normalized (ranging from − 2 to 6 in ESI^+^ mode and − 0.6 to 0.6 in ESI^−^ mode), relative abundance values ≥  − 2 were used to distinguish “background noise” from the easily detectable metabolic species. In ESI^+^ mode, all groups identified as a discriminant in the 2D PCA (Fig. 6A) were the same as those that were clearly distinguishable in the heatmap generated using only m/z_rt pairs with relative abundance values ≥ 2 (Fig. 7A), where group II corresponded to the most distant cluster, composed of samples from the most severe stress concentrations. In the heatmap generated for the ESI^−^ mode data (Fig. 7B), all of the groups were also identified as discriminant by 2D PCA (Fig. 6B), although the identified patterns were not as clear (exclusive) as in ESI^+^ mode. These findings allowed us to determine that the m/z_rt pairs with high Pearson correlation coefficient values identified in the heatmap analysis were distributed throughout the groups identified as a discriminant in the previous 2D PCA.

The results described above reinforce the findings made in the diversity analysis in terms of evenness and diversity of species (J′, H′), since the heatmaps show clear patterns of these findings in both ionization modes, with ESI^+^ mode being the least uniform or excluding and ESI^−^ mode being more equitable. The diversity analyses show for ESI^+^ mode, mainly in late times, clear patterns of the dominance of some species (m/z_rt), where the incidence of species (m/z_rt) associated with prolonged times of stress benefited by making the metabolic community less uniform and therefore less diverse (Fig. 2A, C). The opposite occurs for ESI^−^ mode, where the evenness and prevalence of dominant species (m/z_rt) are lower, given that the values of J′ and H′ show a much more diverse community than in ESI^+^ and therefore, as there is no dominance of species (m/z_rt), a much more uniform community (Fig. 2D, F). This shows that metabolomes are the final product of phenotypic expression that corresponds to dynamic metabolic-adaptive patterns of “behavior,” which translates into phenotypic plasticity derived from the environment (biotic or abiotic factors).

### Identification of differential marker metabolites (m/z_rt) using FC analyses

**Fig. 8:**
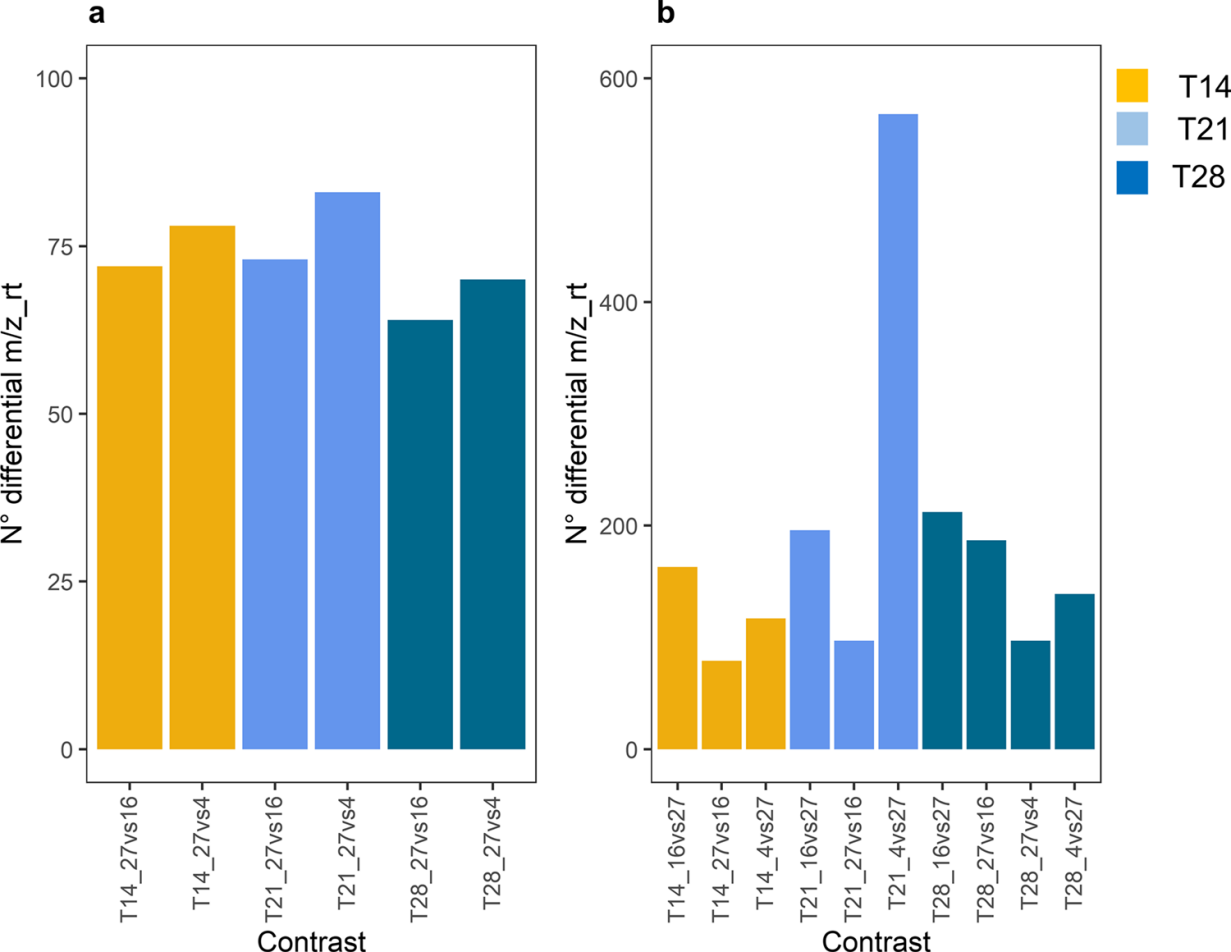
Bar graph representing the number of differential m/z_rt pairs identified by FC in both ESI modes. **a** Differential m/z_rt pairs in ESI^+^ mode, in which the times T14 and T21 at C16 and C4 provide the highest number of differential m/z_rt pairs. **b** Differential m/z_rt pairs identified in datasets from ESI^−^ mode. T21 was the best represented as far as the number of differential m/z_rt pairs was concerned

Marker metabolites (m/z_rt), which are unique for some samples or highly abundant in some time/treatments, were identified by comparing pairs of groups recognized as discriminant (e.g., T14_C27 *vs.* T14_C16; T21_C27 *vs.* T21_C16; T28_C27 *vs.* T28_C16; T28_27 *vs.* T28_C4; see previous results of the 2D PCA). The results obtained showed that after eliminating the redundancy produced by the comparisons performed on the *C. obtusifolia* metabolome dataset generated from ESI^+^ mode, a total of 440 m/z_rt pairs were identified as “differentially synthesized” (Supplementary Tables S9, S10, and Supplementary Fig. S5a–f). The FC method was used for this purpose using a value of 2 (or higher) as the cutoff. Interestingly, the comparisons performed for T14 and T21 followed by the comparisons corresponding to T28 at both concentrations of nitrate deprivation (C16 and C4) were those comparisons in which the highest number of differential m/z_rt pairs was identified (Fig. 8A). Regarding ESI^−^ mode, a total of 875 differential m/z_rt pairs were identified (Supplementary Tables S9, S10, and Supplementary Fig. S6a–j). For this ESI mode (ESI^−^), the T21_C4 *vs.* T21_C27 comparison was that in which the vast majority of m/z_rt pairs differentially synthesized were identified, followed by times T21 and T28 at C16 (Fig. 8B).

**Fig. 9:**
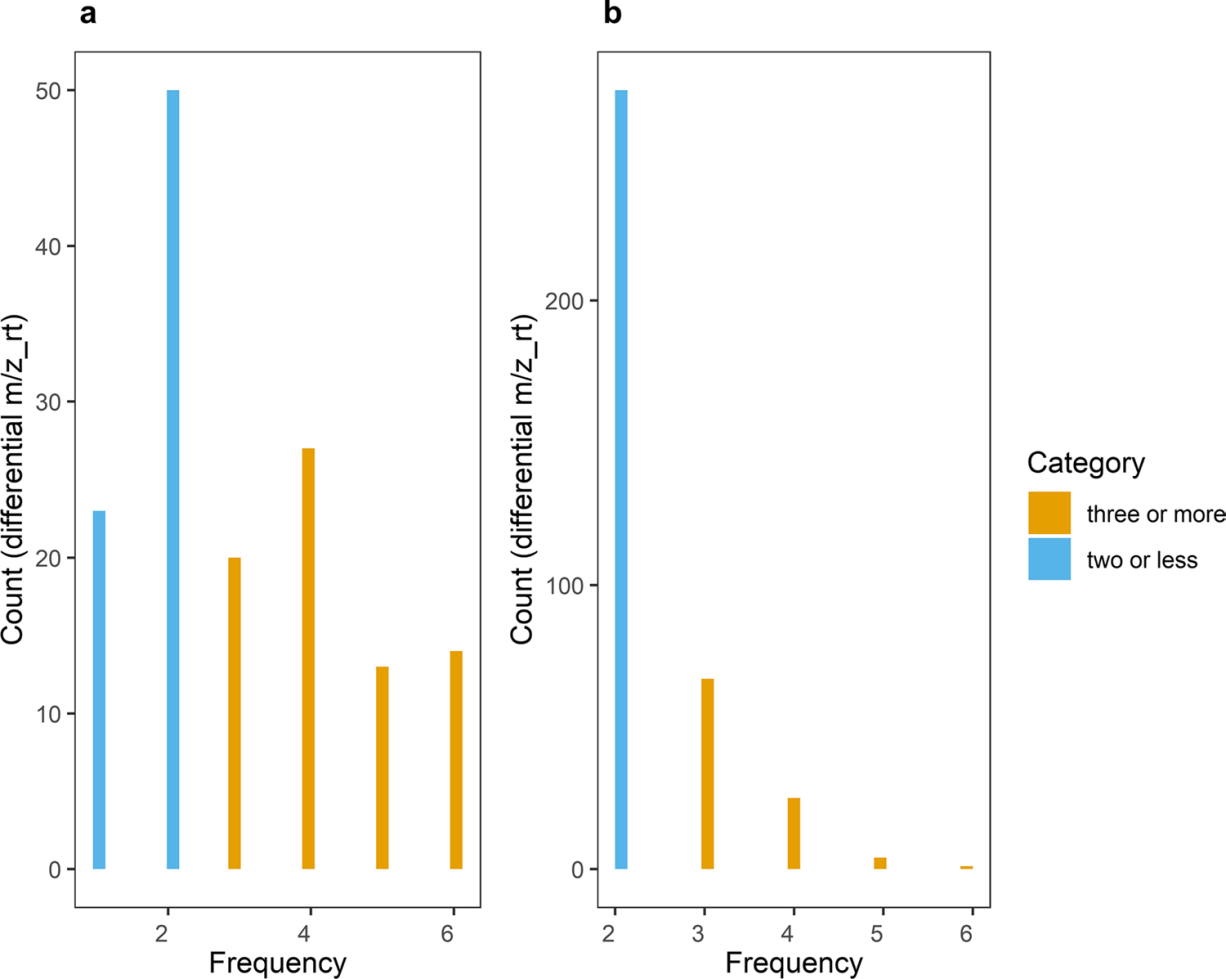
Differential m/z_rt frequencies. Histograms representing the frequencies of each m/z_rt pair after being identified as differential in at least one of the comparisons performed by FC analysis on each of ESI modes. **a** Histogram for differential m/z_rt pairs identified in ESI^+^ mode, where it is shown that most of the differential m/z_rt pairs were identified as such in one, two, or four comparisons (frequencies 1, 2, and 4). **b** Histogram for differential m/z_rt pairs identified in ESI^−^ mode, where it is demonstrated that the majority of differential m/z_rt pairs were identified as such in at least two comparisons

Subsequently, the frequency of those m/z_rt pairs identified as “differentially synthesized/accumulated” was estimated. For that purpose, we quantified the number of times each m/z_rt pairs was identified as differential throughout the comparisons performed. Considering that most comparisons performed were between the nitrate starvation treatments (C16 and C4) and the control group (C27) at the distinct sampling points analyzed (T0, T7, T14, T21, and T28), we defined as the threshold those m/z_rt pairs identified in two comparisons or more, whose changes could be of major relevance in the response to the analyzed stress condition (nitrate starvation). This exercise also helped to considerably reduce the number of metabolites identified as differential and which must be annotated to assign a possible identity to them. In the frequency histogram of ESI^+^ mode (Fig. 9A), the vast majority of differential m/z_rt pairs are in fact present in at least 2 comparisons, i.e., once their abundance increases, they maintain high levels through time, but these times are mainly later times (T14–T28). Meanwhile, differential m/z_rt pairs identified as such in three or more comparisons (from 3 to 6) are less than those identified in two comparisons, and they mainly represent m/z_rt pairs, which are quickly accumulated in response to the stress condition, and their abundance is probably required over time while stress lingers. These m/z_rt pairs (identified in three or more comparisons) also correspond to the m/z_rt group, which not only quickly accumulates but also accumulates regardless of whether the stress is mild or severe (C16 or C4, respectively). In ESI^−^ mode (Fig. 9B), 90% of the differential m/z_rt pairs are present in at least two of the comparisons performed, with 10% being those shared between three or more. As a result, a total of 147 and 371 m/z_rt pairs were obtained in the ESI^+^ and ESI^−^ modes after the reduction performed above. This represents the total number of unique m/z_rt pairs in the metabolome. Of the previously identified m/z_rt pairs, the total (147 and 371) were annotated and used in future analyses (Supplementary Table S10) to avoid distorting the presumptive identification of the compound. This is because although it is known that the same rt could apparently be the same compound, this premise is not necessarily always fulfilled, and the purpose of this method considers a holistic approach in every aspect of it.

### Presumptive annotation of *C. obtusifolia* differential m/z_rt pairs and enrichment analysis of the corresponding metabolic pathways

**Fig. 10:**
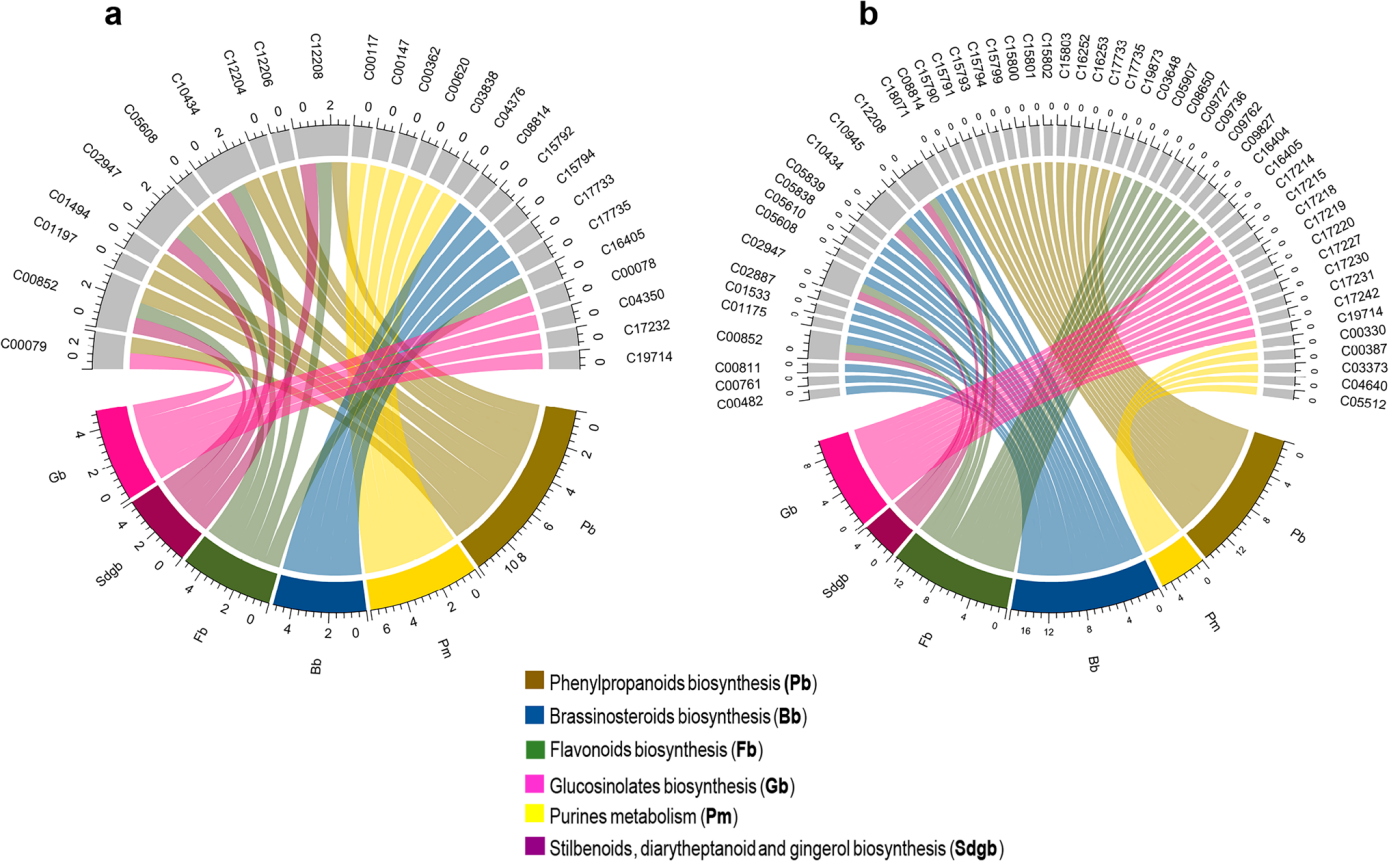
Enrichment analysis of active metabolic pathways in the *C. obtusifolia* metabolome. Six shared active metabolic pathways identified in (**a**) ESI^+^ and (**b**) ESI^−^ modes are shown, and the most enriched pathways are phenylpropanoids, brassinosteroids, flavonoid biosynthesis, purine metabolism, glucosinolate biosynthesis, and stilbenoid, diarylheptanoid, and gingerol biosynthesis

Once the differentially overaccumulated *C. obtusifolia* m/z_rt pairs were identified in the discriminant groups described above (see “Results” for more details), an enrichment analysis of metabolic routes based on the data from both ESI modes was carried out, and their participation in each of the enriched pathways was analyzed. A Mummichog-based MS peak analysis of dMEs was applied for this purpose (see “Materials and methods” for more details). The potential participation of each m/z_rt pair and their annotations predicted from the spectral features were generated based on the available KEGG codes for *A. thaliana*. In the ESI^+^ mode dataset, 112 of the 147 tMEs were assigned a KEGG identifier, among which 86 were nonrepeated (76.70%) (Supplementary Table S11). From the initial 371 tMEs, a total of 215 were assigned a KEGG identifier, among which 130 tMEs were nonrepeated (60.46%) (Supplementary Table S11). Subsequently, enrichment analyses of metabolic pathways were carried out on the datasets from both ESI modes (see “Materials and methods” for more details). As expected, the enrichment analyses grouped many tMEs into more than one pathway. This was mainly due to two reasons: *(i)* the metabolome of a cell represents an elaborate network of interconnected pathways that under many conditions enables the synthesis of extremely complex metabolites, including those related to both primary and secondary metabolism, and *(ii)* similar to other enrichment analyses, after combining Mummichog-based MS peak analysis with the information provided by the KEGG database and its hierarchical classification system, KEGG terms were structured in directed acyclic graphs with a clearly defined hierarchical structure (i.e., a gene or metabolite annotated with any term was also annotated with every ascending term, or parent term, of the more specific term). Thus, each Gene Ontology (GO) category contained all the genes (or metabolites) from each of its descendant categories. Consequently, the major categories in which most genes/metabolites are often grouped were more generalist and, simultaneously, less informative. In our analysis, this was mainly notable in categories such as “metabolic pathways” and “biosynthesis of secondary metabolites,” which were dominant but not very specific (Supplementary Table S12). Therefore, based on this consideration, circular plot representations were generated to illustrate enriched and highly informative categories with respect to the tME biosynthetic pathways represented in both ESI modes (Fig. 10).

The metabolic pathways identified as enriched by differentially synthetized tMEs in ESI^+^ mode (Supplementary Fig. S7a–q) corresponded to phenylpropanoid biosynthesis (with a total of 11 tMEs out of the 51 metabolites involved in the pathway); purine metabolism (with 7 tMes out of 91 involved in the pathway); brassinosteroid biosynthesis (with 5 tMEs out of 27 involved in the pathway); flavonoid biosynthesis (with 5 tMEs out of 68 involved in the pathway); stilbenoid, diarylheptanoid and gingerol biosynthesis (with 4 tMEs out of 24 present in the pathway); and glucosinolate biosynthesis (with 3 tMEs out of 72 involved in the pathway), in relation to the number of active tMEs within each pathway (Fig. 10A and Supplementary Table S12). In ESI^−^ mode, tMEs belonging to at least 15 metabolic pathways were identified (Supplementary Fig. S8a–l), where the pathways of phenylpropanoid biosynthesis (with 17 tMEs out of the 51 involved in the pathway), brassinosteroid biosynthesis (with 14 tMEs out of 27 involved in the pathway), flavonoid biosynthesis (with 13 tMEs out of 68 involved in the pathway), glucosinolate biosynthesis (with 10 tMEs out of 72 involved in the pathway), glucosinolate biosynthesis (with 10 tMEs out of 72 involved in the pathway), and purine metabolism (with 7 tMEs out of 91 involved in the pathway) were the most enriched (Fig. 10B and Supplementary Table S12).

The four previously mentioned pathways enriched by differential tMEs identified in ESI^+^ mode (Table 1) are interesting because they correspond to the phenylpropanoid pathway, and CGA and its precursors (L-phenylalanine, caffeic acid, ferulic acid, *p*-coumaroyl-shikimate, caffeoyl shikimic acid, and *p*-coumaroyl quinic acid) (Cadena-Zamudio et al. 2020; Clifford et al. 2017; Escamilla-Trevino et al. 2014; Sonnante et al. 2010) are among the main key metabolites enriched in this pathway. This finding is consistent with several previous reports demonstrating that nitrate starvation induces CGA biosynthesis (Cadena-Zamudio et al. 2020; Nicasio-Torres et al. 2012). In the brassinosteroid pathway, the brassinolide metabolite end-product of the pathway generated by the conversion of castasterone (another differential tME) is highlighted, suggesting that a broad active hormonal network in *C. obtusifolia* cell suspension cultures is modulated by nitrate deprivation in the medium (Bajguz and Tretyn 2003; Castorina and Consonni 2020). In the flavonoid pathway, CGAs and CGA precursors (caffeate derivatives, caffeoylquinic acid (CGA), *p*-coumaroyl-shikimate, caffeoyl shikimic acid, and *p*-coumaroyl quinic acid) were identified again, as was the secondary metabolite homoeriodictyol chalcone, which exhibits strong antioxidant activities, similar to CGA (He et al. 2020; Panche et al. 2016; Tomac et al. 2020). Finally, in the stilbenoid, diarylheptanoid, and gingerol pathways, CGA (and some CGA precursors) were identified as the main precursor.

**Table 1:** Differential tMEs identified by accurate MS in ESI^+^ mode, and biosynthetic pathways

Metabolite	Formula	m/z	Retention time (RT)	Mass Error (ppm)	Hypothetical ion	Kegg ID	Levels of metabolite identification (LMI)
Phenylpropanoid biosynthesis							
L-Phenylalanine	C_9_H_11_NO_2_	166.0865	1.56	≤ ± 5	[M + H]^+^	C00079	3
Caffeic acid	C_9_H_8_O_4_	145.0286	2.88	≤ ± 5	[M + H]^+^	C01197	3
Sinapinic acid	C_11_H_12_O_5_	207.0655	3.06	≤ ± 5	[M + H]^+^	C00482	3
* p*-Coumaroyl-shikimate	C_16_H_16_O_7_	303.0868	2.49	≤ ± 5	[M + H]^+^	C02947	3
* p*-Coumaroyl quinic acid	C_16_H_18_O_8_					C12208	
Caffeoyl shikimic acid	C_16_H_16_O_8_	355.1027	2.44	≤ ± 5	[M + H]^+^	C10434	3
* p*-Coumaraldehyde	C_9_H_8_O_2_	131.0492	3.04	≤ ± 5	[M + H]^+^	C05608	3
Caffeyl alcohol	C_9_H_10_O_3_					C12206	
Caffeoylquinic acid (CGA)	C_16_H_18_O_9_	377.0844	2.9	≤ ± 5	[M + Na]^+^	C00852	3
Ferulic acid	C_10_H_10_O_4_	159.0443	3.05	≤ ± 5	[M + H]^+^	C01494	3
5-Hydroxyconiferaldehyde	C_10_H_10_O_4_					C12204	
Brassinosteroids biosynthesis							
Brassinolide	C_28_H_48_O_6_	503.3366	8.86	≤ ± 5	[M + Na]^+^	C08814	3
Castasterone	C_28_H_48_O_5_	469.331	8.59	≤ ± 5	[M + Na]^+^	C15794	3
7-Oxateasterone	C_28_H_48_O_5_	487.3417				C17733	
7-Oxatyphasterol	C_28_H_48_O_5_					C17735	
3-Dehydroteasterone	C_28_H_46_O_4_	519.3315	7.39	≤ ± 5	[M + Na]^+^	C15792	3
Flavonoids biosynthesis							
Caffeoylquinic acid (CGA)	C_16_H_18_O_9_	377.0844	2.9	≤ ± 5	[M + Na]^+^	C00852	3
* p*-Coumaroyl-shikimate	C_16_H_16_O_7_	303.0868	2.49	≤ ± 5	[M + H]^+^	C02947	3
* p*-Coumaroyl quinic acid	C_16_H_18_O_8_				[M + H]^+^	C12208	
Homoeriodictyol chalcone;	C_16_H_14_O_6_					C16405	
Caffeoyl shikimic acid	C_16_H_16_O_8_	355.1027	2.44	≤ ± 5	[M + H]^+^	C10434	3
Stilbenoids, diarylheptanoid and gingerol biosynthesis							
Caffeoylquinic acid (CGA)	C_16_H_18_O_9_	377.0844	2.9	≤ ± 5	[M + Na]^+^	C00852	3
* p*-Coumaroyl-shikimate	C_16_H_16_O_7_	303.0868	2.49	≤ ± 5	[M + H]^+^	C02947	3
* p*-Coumaroyl quinic acid	C_16_H_18_O_8_					C12208	
Caffeoyl shikimic acid	C_16_H_16_O_8_	355.1027	2.44	≤ ± 5	[M + H]^+^	C10434	3

On the other hand, in ESI^−^ mode, some CGA precursor metabolites (and CGA itself) and other tMEs belonging to the phenylpropanoid pathway were identified in larger amounts (Table 2). Coniferin and syringin were also identified as tMEs. Both metabolites are precursors of monolignol units usually found in lignified tissues, but in our cell suspension cultures, they could be secondary metabolites (tMEs) involved in detoxification and/or cell/tissue differentiation (Le Roy et al. 2016). The brassinosteroid pathway was another pathway enriched by differential tMEs identified in both ESI modes. However, the number of tMEs identified in ESI^−^ mode was considerably higher than that identified in ESI^+^ mode (approximately 50% of total metabolites belonging to this pathway were found). For example, the presence of (22R, 23R)-22,23 dihydroxy-campesterol and (22R, 23R)-22,23-dihydroxy-campest-4-en-3-one stands out, as they are direct precursors of both castasterone and brassinolide (Bajguz and Tretyn 2003; Castorina and Consonni 2020). In the flavonoid pathway, a single precursor of CGA (*p*-coumaroyl quinic acid) was identified, as was (-)-epicatechin, which is one of the major modulators of signaling in response to redox states and regulates enzymes that generate superoxide anions, hydrogen peroxide, and nitric oxide (Fraga et al. 2018). Finally, in the glucosinolate pathway, only methionine metabolism precursors were found; these metabolites are involved in growth regulator signaling-, circadian clock-, and biomass-related pathways, among other functions (Jeschke et al. 2019). Even though the two ESI modes jointly represented the *C. obtusifolia* metabolome in response to nitrate starvation, there were some notable differences related to the ESI mode selected to generate the spectral database. It is also possible that other technical aspects need to be optimized, such as the cone voltage, to generate spectra containing characteristic fragmentation patterns while maintaining the integrity of the molecular ions to ensure accuracy (as done in this study case; see details in Cadena-Zamudio et al. 2020). Despite the annotation being considered putative, the pathway enrichment analysis performed once differential tMEs were identified showed that the two ESI^+/−^ modes shared only six enriched pathways (brassinosteroids, flavonoids, glucosinolates, phenylpropanoids, purine metabolism and stilbenoids, diarylheptanoid, and gingerol biosynthesis), and these pathways shared some but not all the tMEs that could be further annotated. While these pathways might be the most affected by nitrate starvation, it is also possible that some of these tMEs could be necessary to respond to nitrate deprivation and adapt to the lack of this essential macronutrient to maintain cellular viability. A broad marked dominance pattern of tMEs related to CGA biosynthesis was identified in ESI^+^ mode, while the diversity of the identified tMEs was broader in ESI^−^ mode. These findings fit very well with the findings previously obtained in the diversity and chemometric analyses, where both the H′ and the evenness index (J′) complemented the results obtained from the annotation of the data from both ESI modes. It was found at the later points in the kinetic time course that the levels of various tMEs decreased, which indicated that rare species (m/z_rt) were identified under high stress levels and that their presence was therefore related to the maintenance of *C. obtusifolia* cellular homeostasis to a greater extent than that of other species. Such conditions would produce less diverse and therefore less uniform metabolic communities.

**Table 2:** Differential tMEs identified by accurate MS in ESI^−^ mode, and biosynthetic pathways

Metabolite	Formula	m/z	Retention time (RT)	Mass Error (ppm)	Hypothetical ion	Kegg ID	Levels of Metabolite identification (LMI)
Phenylpropanoids biosynthesis							
Sinapoyl malate	C_15_H_16_O_9_	339.0708	1.69	≤ ± 5	[M-H]^−^	C02887	3
* p*-Coumaroyl quinic acid	C_16_H_18_O_8_	353.0866	1.93	≤ ± 5	[M-H]^−^	C12208	3
Caffeoyl quinic acid (CGA)	C_16_H_18_O_9_					C00852	
Coniferin	C_16_H_22_O_8_	387.1286	2.69	≤ ± 5	[M-H]^−^	C00761	3
Syringin	C_17_H_24_O_9_					C01533	
Sinapic acid	C_11_H_12_O_5_	264.0869	2.9	≤ ± 5	[M-H]^−^	C00482	3
Sinapaldehyde	C_11_H_12_O_4_	223.0602	3.02	≤ ± 5	[M-H]^−^	C05610	3
2-coumarinate	C_9_H_8_O_3_					C05838	
Caffeic aldehyde	C_9_H_8_O_3_					C10945	
Coniferyl aldehyde	C_10_H_10_O_3_					C02666	
* p*-Coumaric acid	C_9_H_8_O_3_					C00811	
β-D-Glucosyl-2-coumarate; *cis*-β-D-Glucosyl-2-hydroxycinnamate	C_15_H_18_O_8_	371.0973	3.31	≤ ± 5	M + FA-H	C05839	3
* p*-coumaroyl shikimic acid	C_16_H_16_O_7_	335.076	3.46	≤ ± 5	[M-H]^−^	C02947	3
caffeoyl shikimic acid	C_16_H_16_O_8_					C10434	
1-O-Sinapoyl-β-D-glucose	C_17_H_22_O_10_	367.1024	3.56	≤ ± 5	M-H20-H	C01175	3
* p*-Coumaraldehyde	C_9_H_8_O_2_	188.0708	4.67	≤ ± 5	[M-H]^−^	C05608	3
N1,N5,N10-Triferuloyl spermidine	C_37_H_43_N_3_O_9_	754.2189	5.72	≤ ± 5	[M-H]^−^	C18071	3
Brassinosteroids biosynthesis							
Brassinolide	C_28_H_48_O_6_	501.321	8.82	≤ ± 5	[M + Cl]^−^	C08814	3
(22R,23R)-22,23-Dihydroxy-campest-4-en-3-one	C_28_H_46_O_3_	489.3572	8.02	≤ ± 5	[M + Cl]^−^	C16253	3
Castasterone	C_28_H_48_O_5_	485.3256	9.06	≤ ± 5	[M + Cl]^−^	C15794	3
7-Oxateasterone	C_28_H_48_O_5_					C17733	
7-Oxatyphasterol	C_28_H_48_O_5_					C17735	
6-Deoxoteasterone	C_28_H_50_O_3_	455.3517	10.55	≤ ± 5	[M + Cl]^−^	C15799	3
6-Deoxotyphasterol	C_28_H_50_O_3_					C15801	
Teasterone	C_28_H_48_O_4_	469.3313	11.46	≤ ± 5	[M + Cl]^−^	C15791	3
Typhasterol	C_28_H_48_O_4_					C15793	
3-Dehydro-6-deoxoteasterone	C_28_H_48_O_3_	453.3363	12.21	≤ ± 5	[M + Cl]^−^	C15800	3
6-Deoxocastasterone	C_28_H_50_O_4_	471.3467	10.45	≤ ± 5	[M + Cl]^−^	C15802	3
6α-Hydroxy-castasterone	C_28_H_50_O_5_	487.3417	7.47	≤ ± 5	[M + Cl]^−^	C15803	3
Cathasterone	C_28_H_48_O_3_	453.3363	12.21	≤ ± 5	[M + Cl]^−^	C15790	3
(22R,23R)-22,23-Dihydroxycampesterol	C_28_H_48_O_3_	453.3364	14.11	≤ ± 5	[M + Cl]^−^	C16252	3
Flavonoids biosynthesis							
Caffeoyl quinic acid (CGA)	C_16_H_18_O_9_	353.0866	1.93	≤ ± 5	[M + Cl]^−^	C00852	3
* p*-Coumaroyl quinic acid	C_16_H_18_O_8_					C12208	
4-Coumaroylshikimate	C_16_H_16_O_7_	301.0706	2.47	≤ ± 5	[M + Cl]^−^	C02947	3
(-)-Epicatechin	C_15_H_14_O_6_	375.076	3.66	≤ ± 5	[M + Cl]^−^	C09727	3
5-O-Caffeoylshikimic acid	C_16_H_16_O_8_					C10434	
Isoliquiritigenin	C_15_H_12_O_4_	301.0702	1.98	≤ ± 5	[M + Cl]^−^	C08650	3
Liquiritigenin	C_15_H_12_O_4_					C09762	
Pinocembrin	C_15_H_12_O_4_	315.086	3.72	≤ ± 5	[M + Cl]^−^	C09827	3
Glucosinolates biosynthesis							
2-(3’-Methylthio)propylmalic acid	C_8_H_14_O_5_S	281.0685	1.41	≤ ± 5	[M + Cl]^−^	C17214	3
3-(3’-Methylthio)propylmalic acid	C_8_H_14_O_5_S					C17215	
2-(4’-Methylthio)butylmalic acid	C_9_H_16_O_5_S					C17218	
3-(4’-Methylthio)butylmalic acid;	C_9_H_16_O_5_S					C17219	
2-Oxo-7-methylthioheptanoic acid	C_8_H_14_O_3_S	191.0551	1.39	≤ ± 5	[M + Cl]^−^	C17220	3
3-(6’-Methylthio)hexylmalic acid	C_11_H_20_O_5_S	301.0703	3.73	≤ ± 5	[M + Cl]^−^	C17227	3
2-(7’-Methylthio)heptylmalic acid	C_12_H_22_O_5_S	317.086	3.15	≤ ± 5	[M + Cl]^−^	C17230	3
3-(7’-Methylthio)heptylmalic acid	C_12_H_22_O_5_S	279.1074	1.71	≤ ± 5	[M + Cl]^−^	C17231	3
S-(4-Methylthiobutylthiohydroximoyl)-L-cysteine	C_8_H_16_N_2_O_3_S_2_	125.0232	1.7	≤ ± 5	[M + Cl]^−^	C17242	3
(*E*)-Phenylacetaldoxime	C_8_H_9_NO	180.0657	0.85	≤ ± 5	[M + Cl]^−^	C19714	3

## Discussion

Here, we proved that spectral databases obtained by accurate MS analysis in both positive and negative ESI modes showed important differences in terms of the number of distinctive m/z_rt data pairs that could be obtained in an untargeted metabolomic study. This and likely other technical aspects, such as the types of adducts, number of replicates, cone voltage, and the mass spectrometer configuration mode (high-resolution mode (less sensitivity) or sensitivity mode (less resolution)) involved, could influence the results, hypotheses, and conclusions that can be obtained from the corresponding mass spectra. The applied analyses showed a distinctive pattern of the spectrometric features generated in ESI^+^ mode when they are compared against those obtained in ESI^−^ mode. Based on a single dataset (i.e., ESI^+^), it could be concluded that the control condition (C27) was characterized by a complex metabolome, whereas diversity tended to decrease when *C. obtusifolia* cell suspension cultures were grown under nitrate starvation conditions (Béguinot 2016; Thompson and Withers 2003; Ugland et al. 2003). After analyzing the results obtained from the SAC and estimated Shannon, Simpson, and Pielou diversity indices (Fig. 1A; 3A and Supplementary Table S4), it could be concluded that when nutritional resources (N) are compromised, fewer m/z_rt pairs (metabolic species) are synthetized (Colwell et al. 2004; Mao et al. 2005). The availability of resources also seems to increase the presence (synthesis) of uncommon m/z_rt pairs; i.e., it may favor the de novo synthesis of metabolites involved in the response to stress conditions and subsequent maintenance of cell viability (Avolio et al. 2015, 2019). If only the conclusions arising from the spectral database generated in ESI^−^ mode are considered, it can be hypothesized that a diverse metabolome with a small number of uncommon m/z_rt pairs is required to respond to the tested stress conditions, which will facilitate the synthesis of the identified components but will require very high concentrations to respond to the external stimulus (Fig. 1B; 3D and Supplementary Table S4). This conclusion is incorrect and highlights the importance of working with data obtained in both ESI modes because, together, they represent the whole metabolome of *C. obtusifolia* cell suspension cultures when grown under different nitrate concentrations. Even though it is possible to discuss other aspects that could influence the results (for example, the fact that we only analyzed ionizable molecules), it is clear that the spectral features generated in ESI^+^ and ESI^−^ modes are complementary and that the results obtained in both modes need to be considered together to obtain a detailed view of cellular responses and how the cell modulates its metabolome in response to specific stresses.

Heatmap analyses confirmed the findings described above; thus, it was possible to identify a series of constant, recognizable variables in both ionization modes with correlation values close to 1 (Supplementary Tables S7, S8) under Pearson correlation coefficient analysis contrasting m/z_rt *vs.* m/z_rt. The comparison of m/z_rt *vs.* the sample was studied (Fig. 7), and encouraging results were obtained, confirming that the groups identified as differential by 2D PCA in both ionization modes consisted of the m/z_rt pairs with high correlation values and high relative abundance (Fig. 6). The previous results allowed the identification of marker metabolites within the discriminant groups via FC analyses, in addition to metabolic pathway enrichment analysis and the presumptive annotation of m/z_rt pairs (tMEs) in public spectral libraries (Billet et al. 2020; Kasote et al. 2020; Rossouw et al. 2019). The results made it possible to identify a total of 141 and 371 differential m/z_rt pairs in ESI^+^ and ESI^−^ modes, respectively, from the discriminant groups previously identified by 2D PCA (see “Results” for more details); only 86 and 130 of these pairs were annotated with KEGG codes (Supplementary Tables S10, S11). Subsequently, the enrichment analysis yielded a total of 20 and 15 enriched metabolic pathways (Supplementary Table S11), including the pathways involved in brassinosteroid, flavonoid, glucosinolate, phenylpropanoid, stilbenoid, diarylheptanoid, and gingerol biosynthesis as well as purine metabolism. These pathways were common to the datasets obtained in both ESI modes. Based on the presumptive annotation of differentially synthesized m/z_rt pairs (dMEs), not only was the reconstruction of metabolic pathways performed for the remaining pathways (Supplementary Fig. S6a-q; Fig. S7a-l), but their interactions were observed in a Circos plot (Fig. 10). The importance of including the spectral databases generated in both ESI modes was demonstrated again in this case, especially considering that the number of tMEs within in some enriched metabolic pathways was highly variable depending on the ESI mode (see flavonoid biosynthesis as an example; Fig. 10).

Interestingly, some of the enriched pathways were directly involved in the response to abiotic stress, as the tested type of stress (nitrate starvation) can increase the generation of reactive oxygen species (ROS), such as superoxide radicals (O_2_^−^), hydroxyl radicals (OH^−^), perhydroxyl radicals (HO_2_^*−*^), alkoxyl radicals, (RO^−^), hydrogen peroxide (H_2_O_2_), and singlet oxygen ^1^O_2_ (Anjum et al. 2015; Vardhini and Anjum 2015). Because the number of tMEs involved in the brassinosteroid (BR) pathway was quite high (representing 51.8% of the total), a deeper literature review was performed to explain the biological importance of these types of metabolites and their participation in the nitrate starvation response, since these compounds play an important role in relieving various types of stress, such as drought, cold, heat, salinity, and nutritional stress (Houimli et al. 2010). Such stress conditions are characterized by increases in the activity of antioxidant enzymes such as superoxide dismutase (SOD), catalase (CAT), ascorbate peroxidase (APX), and peroxidase (POX), all of which are key enzymes involved in the release and dismutation of H_2_O_2_ in H_2_O and O_2_ (Anwar et al. 2018; Basit et al. 2021; Guedes et al. 2021; Hu et al. 2021) by regulating various metabolic pathways, which regulate signal transduction pathways in conjunction with many other growth regulators, such as indole-3 acetic acid, abscisic acid, jasmonic acid, zeatin riboside, isopentenyl adenosine, and gibberellin (GA), to stimulate tolerance to nutritional stress, thus maintaining cellular homeostasis in the plant (Anwar et al. 2018; Hafeez et al. 2021; Manghwar et al. 2022). In this context, the presence of 22R and 23R compounds, also known as 24-epibrassinolide (EBR), is highlighted; this is one of the most active forms of BR (Manghwar et al. 2022) and has multiple functions in metabolism contributing to fundamental processes such as vascular differentiation, germinative processes, root and stem growth, fruit development, abscission and maturation, gene expression modulation by inducing CHLASE, CHS, PAL, POD, CAT, GR, and GST1 genes, and the enhancement of tolerance mechanisms (Houimli et al. 2010; Janeczko et al. 2010; Li et al. 2020; Guedes et al. 2021; Manghwar et al. 2022), resulting in substantial changes in biomass (Guedes et al. 2021; Manghwar et al. 2022). These characteristics have caused EBR to be considered a key active molecule in the mitigation of biotic and abiotic stress (Guedes et al. 2021). In addition, brassinolide, which was also identified as an active metabolite in this pathway, is the final product of the pathway; it is generated by the conversion of castasterone and is involved in abiotic stress responses, including the response to nitrate starvation, among other functions (Bajguz and Tretyn 2003; Castorina and Consonni 2020). The presence of the Bb pathway was expected, since it plays a fundamental role in regulating plant growth and development, specifically by modulating cell expansion and division processes (Ahammed et al. 2020; Nolan et al. 2020; Sharma et al. 2017; Vardhini and Anjum 2015). It has recently been shown that these steroid hormones are involved in the modulation of autophagy in response to nitrogen starvation (Ahammed et al. 2020; Wang et al. 2019) through the expression of BZR1 genes, which improve autophagosome formation and tolerance to nitrogen starvation (Ahammed et al. 2020; Wang et al. 2019), in addition to regulating the entry of ions; these changes translate into higher efficiencies of light energy transformation, CO_2_ conductivity, and the photosynthetic rate, in which BRs have been shown to have a crucial role. These effects are capable of increasing nitrogen input levels, reducing toxic ions (ROS), and promoting ion homeostasis, thus enhancing the activity of enzymes such as glutamine synthetase (GS), glutamate synthase (GOGAT), glutamate dehydrogenase (GDH), nitrate reductase (NR), and nitrite reductase (NiR). The last two enzymes are closely associated with nitric oxide (NO), a small gas molecule related to redox that has an essential effect on seed germination, senescence, adventitious root development, and stomatal closure, making NO an essential molecule in plant responses to abiotic stress (Anwar et al. 2018; Jia et al. 2020; Jia et al. 2021; Kong et al. 2021; Kothari and Lachowiec 2021).

The above results strongly support the identification of BR biosynthesis as one of the most enriched pathways, since these steroid hormones play a fundamental role in the positive regulation of stress resistance genes; in addition, these steroid hormones interact with other metabolic pathways; synergistically interact with other phytohormones to regulate a variety of physiological and developmental processes in plants; regulate the activity of antioxidant enzymes, chlorophyll concentrations, and the photosynthetic capacity; reduce the transpiration rate; modulate metabolic changes involved in nutrient sensing and stomatal oscillation; and enhance the effect of Rubisco activity in the context of abiotic stress (Anwar et al. 2018; Nolan et al. 2020; Guedes et al. 2021). These findings allowed us to obtain deeper insight into the signal transduction of BRs and their integration at the physiological and genetic levels as reflected in the metabolome of *C. obtusifolia*, thus providing a more comprehensive overview of abiotic stress tolerance mechanisms due to nutritional deficits.

In the biosynthetic pathways of phenylpropanoids, flavonoids, stilbenoids, diarylheptanoid, and gingerol, CGA and most of the precursors involved in its biosynthesis were identified in positive, negative, or both ESI modes (see, for example, *p*-coumaroyl shikimic acid, caffeoyl shikimic acid, *p*-coumaroyl quinic acid, coniferin, and syringin, which are precursors of monolignol units mainly found in lignifying tissues). Considering that the examined spectral libraries were obtained from cell suspension cultures, it can be hypothesized that some of these tMEs may have simple storage or detoxification functions in certain stages of cell or tissue differentiation when growth is compromised (Le Roy et al. 2016). (-)–Epicatechin, which was solely identified as a differentially synthesized tME in the spectrometric database generated in ESI^−^ mode, is one of the major modulators of signaling in response to redox states and regulates enzymes responsible for generated superoxide, hydrogen peroxide, and nitric oxide (Fraga et al. 2018). The abovementioned results are very reasonable considering that the examined *C. obtusifolia* cell suspension cultures were under nutritional stress imposed by nitrogen deficiency treatments. It has been demonstrated that the amount of nitrate available to a plant has an important impact on the presence of phenolic compounds (Cadena-Zamudio et al. 2020; Kováčik and Klejdus 2014; Nicasio-Torres et al. 2012). This supports the hypothesis of carbon and nutrient balance (CNB) (Bryant et al. 1983) and the protein competition model (PCM) (Jones and Hartley 1999), indicating that when the biosynthesis of phenolic compounds, as metabolites specialized in the management of ROS, is increased, it compromises plant growth under stress until the stress condition can be overcome. Finally, in the glucosinolate pathway, only methionine metabolism precursors were found. These precursors participate in plant growth regulator signaling-, circadian clock-, and biomass-related functions, among others (Jeschke et al. 2019).

Finally, the metabolomic findings obtained after implementing the ecological-metagenomic analyses stand out, since it was possible to quantify the abundance of metabolic species (m/z_rt) depending on abiotic stress induced by nitrate deficiency over time, where the late time points under stress conditions (C16 and C4) were characterized by the fewest metabolic species (m/z_rt). In addition, it was possible to decipher the population structure of the *C. obtusifolia* metabolome. Here, the analysis of SACs led to the conclusion that the incidence of “rare” species was strongly associated with abiotic stress due to the nutritional deficit over time, which was more obvious in ESI^+^ mode. These findings were reinforced by the diversity analyses (SACs, RACs, H′, DSi, and J′) indicating that diversity decreases as stress increases. This leads to the induction and biogenesis of “rare species” that then become dominant, resulting in a less uniform population structure (predominance of one or a few species). The induction of these dominant species seems to be involved in the maintenance of cellular homeostasis, in which viability is compromised to ultimately overcome stress conditions and successfully achieve a new standard physiological state. In conclusion, the analytical tools of the ecological-metagenomic type used in this study revealed a more comprehensive and global vision of what metabolic “behavior” is. In biological terms, this manifests as an effect of phenotypic plasticity, and the driving or restrictive variables of the *C. obtusifolia* metabolome were identified in this context. These inferences could be extrapolated to much larger studies performed under uncontrolled conditions (field) in which niche communities of botanical taxa that are of commercial or biomedical interest are analyzed. This approach, in conjunction with a global metabolomic approach, allows a comprehensive, quantitative, and above all impartial vision of the metabolome to be obtained, as reported in this work.

## Conclusions

In recent years, the application of metabolomics in plant science has increased exponentially, although its application is still relatively limited relative to other omics approaches, such as genomics, metagenomics, transcriptomics, and proteomics. Therefore, in the present study, an unorthodox but reliable approach was presented to analyze large metabolomic datasets, implementing tools that are generally used in population ecology and helping to detect patterns of metabolic species diversity, richness, and distribution associated with abiotic factors on a time-course scale. In addition, a high-performance annotation method using an integrative workflow based on a cooccurrence network algorithm was proposed, in which metabolites were annotated and classified in an efficient and reliable way.

Although most of the obtained annotations still need to be inspected and validated manually (using chemical standards and nuclear magnetic resonance), consensus and greater confidence in the interpretation of high-throughput mass data can be achieved by using different but complementary computational approaches, such as those presented in this work. It is concluded that by applying the proposed workflow based on ecological-type tools, the present study was able to categorize and identify metabolic species and discern population patterns through the use of databases generated in both ESI^+/−^ ionization modes. These analyses were based on accurate mass spectrometry performed in tandem to avoid biases favoring erroneous conclusions and achieve greater coverage of the metabolome, allowing us to understand and analyze the phenotype and its plasticity in greater detail according to the conditions (biotic/abiotic) experienced by the species. Therefore, it is expected that this workflow will allow the precision of in silico metabolite annotation to be improved and will increase the speed of metabolomic pattern analysis in plant biology.

## Supplementary Information

Below is the link to the electronic supplementary material.

Supplementary file1 (XLSX 11731 KB)

Supplementary file2 (PDF 9898 KB)

## Data Availability

All data generated or analyzed during this study are included in this published article [and its supplementary information files].
