# Supplementary material for: The use of ecological analytical tools as an unconventional approach for untargeted metabolomics data analysis: the case of Cecropia obtusifolia and its adaptive responses to nitrate starvation
Source: Funct Integr Genomics. 2022 Oct 6;22(6):1467–93. doi: 10.1007/s10142-022-00904-1 (PMC9701659; doi:10.1007/s10142-022-00904-1)
Supplement: Supplementary file 2 — Supplementary file2 (PDF 9898 KB) [file 10142_2022_904_MOESM2_ESM.pdf]

The use of ecological analytical tools as an unconventional approach for untargeted metabolomics data analysis: The case of *Cecropia obtusifolia* and its adaptive responses to nitrate starvation.

Jorge David Cadena-Zamudio[1], Juan Luis Monribot-Villanueva[1], Claudia-Anahí Pérez-Torres[2], Fulgencio Alatorre-Cobos[3], Beatriz Jiménez-Moraila[4], José A. Guerrero-Analco[1], and Enrique Ibarra-Laclette[1]*

[1] Red de Estudios Moleculares Avanzados (REMAV), Instituto de Ecología, A.C. (INECOL), Xalapa, Veracruz, México
[2] Investigador por México - CONACYT en la Red de Estudios Moleculares Avanzados (REMAV), Instituto de Ecología, A.C. (INECOL), Xalapa, Veracruz, México
[3] Investigador por México - CONACYT en el Campus Campeche, Colegio de Postgraduados (COLPOS), Campeche, México
[4] Laboratorio Nacional de Genómica y Biodiversidad (LANGEBIO), Centro de Investigación y de Estudios Avanzados, IPN, Irapuato, Guanajuato, México

Corresponding Author:
Enrique Ibarra-Laclette[1]
Carretera antigua a Coatepec 351, Col. El Haya, Xalapa, Veracruz, 91073, México
Email address: enrique.ibarra@inecol.mx

Supplementary Figures

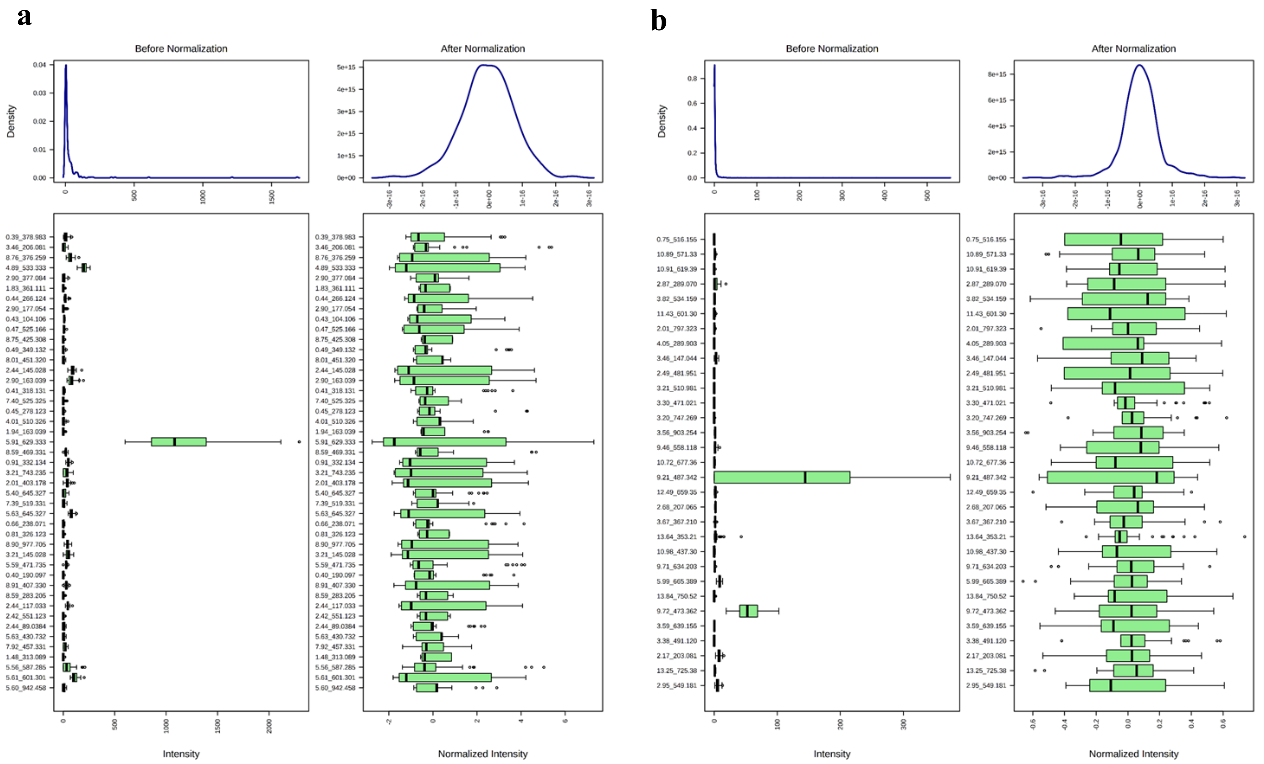

Fig. S1. Raw data normalization for both ESI modes. **(a)** Normalization of raw data corresponding to ESI$^+$ mode, using the non-transformed mean values and autoscaling method. **(b)** Normalization of raw data corresponding to ESI$^-$ mode, using the cube root transformation and the range scaling method (centered on the mean and divided by the range of each variable)

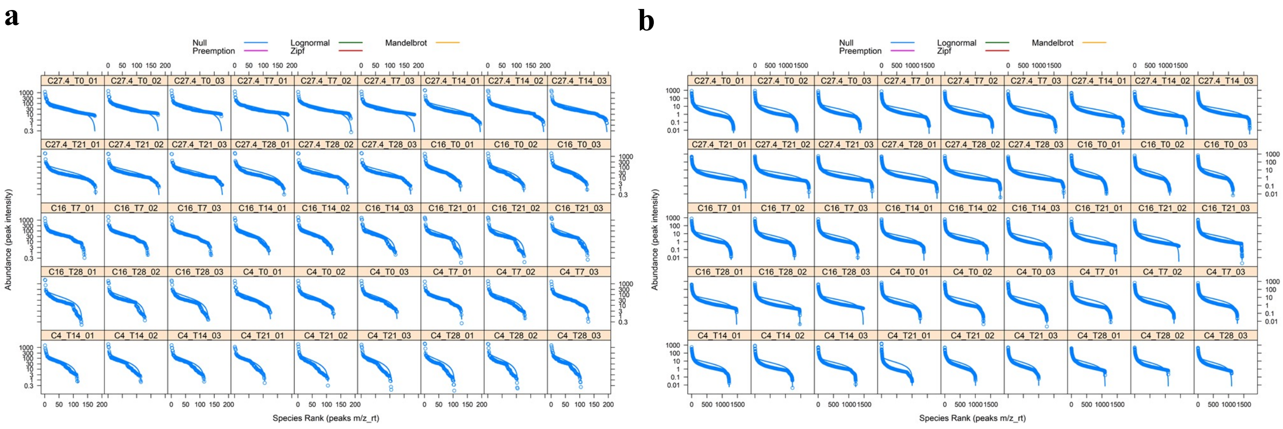

Fig. S2 Rank abundance curves of *C. obtusifolia* metabolome species in both ionization modes. **(a)** Rank abundance curves of positive electrospray ionization mode. **(b)** Rank abundance curves of negative electrospray ionization mode. Both RAC curves show the relevance of richness and accumulation in the Null model, indicating that biological distribution of species is regulated by an important factor (abiotic stress) and shared uniformly by the species

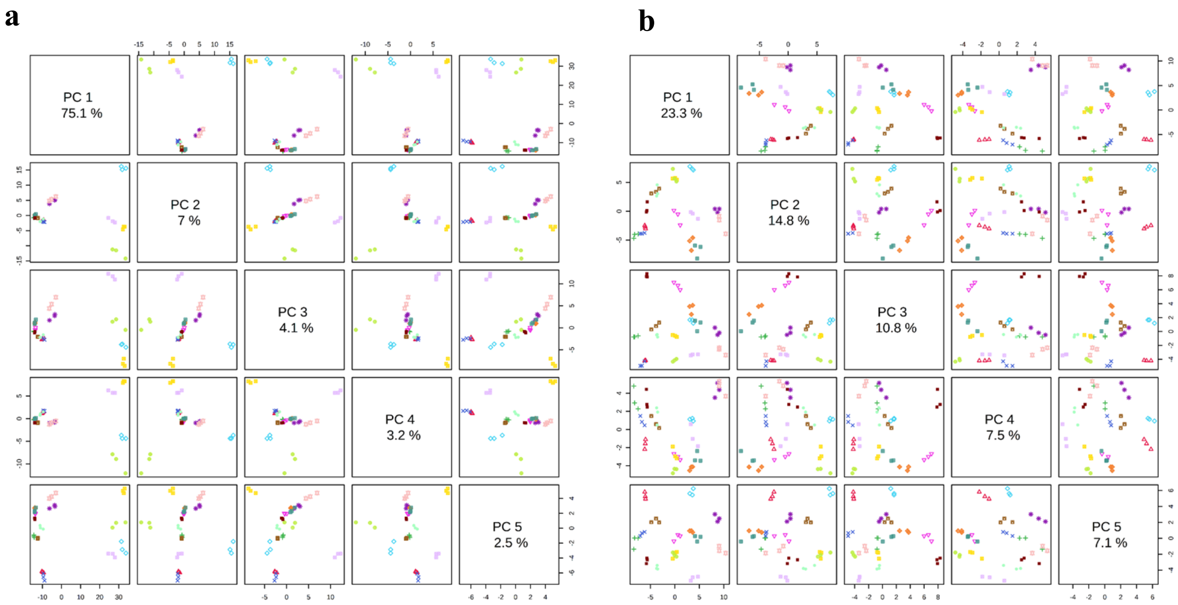

Fig. S3 Principal component analysis. **(a)** The explanatory percentage of variance values are shown for the ESI^+ mode, where the first two components are those that contribute the most to the variance. **(b)** The explanatory percentage of variance values are shown for the ESI^- mode, where again, the first two components are those that contribute the most to the variance

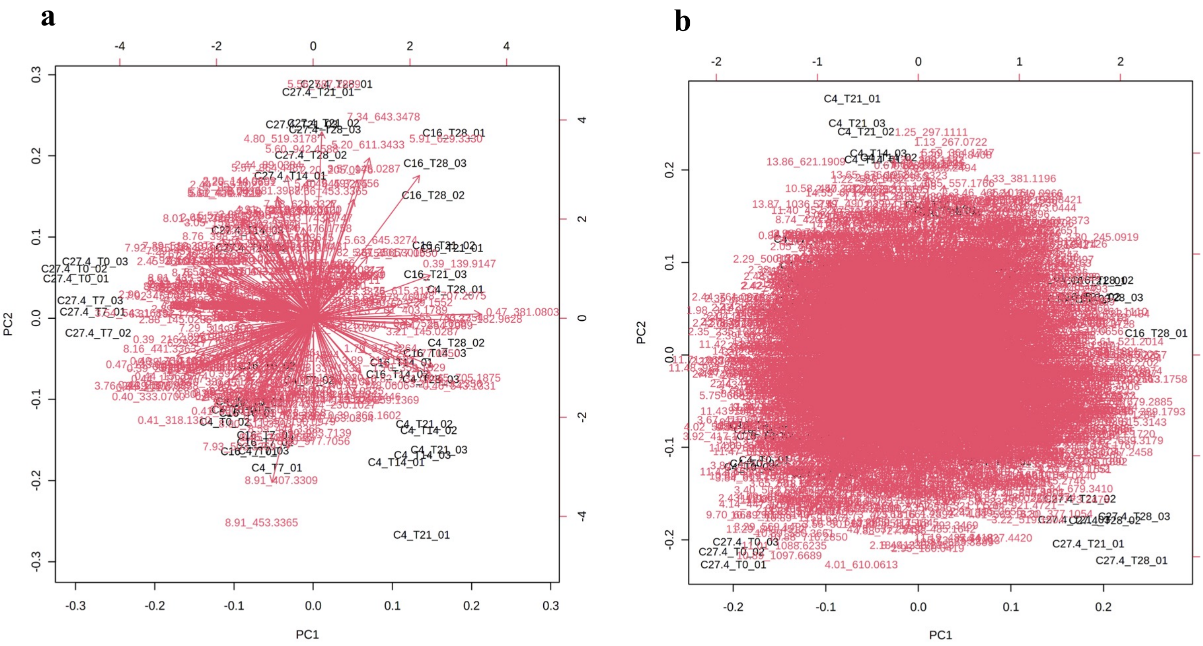

Fig. S4 PCA biplot from 2D PCA analysis. **(a)** The among distributions of each variable of each component are shown in the ESI^{+} mode. **(b)** The loadings distributions to each variable of each component for the ESI^{-} mode are shown

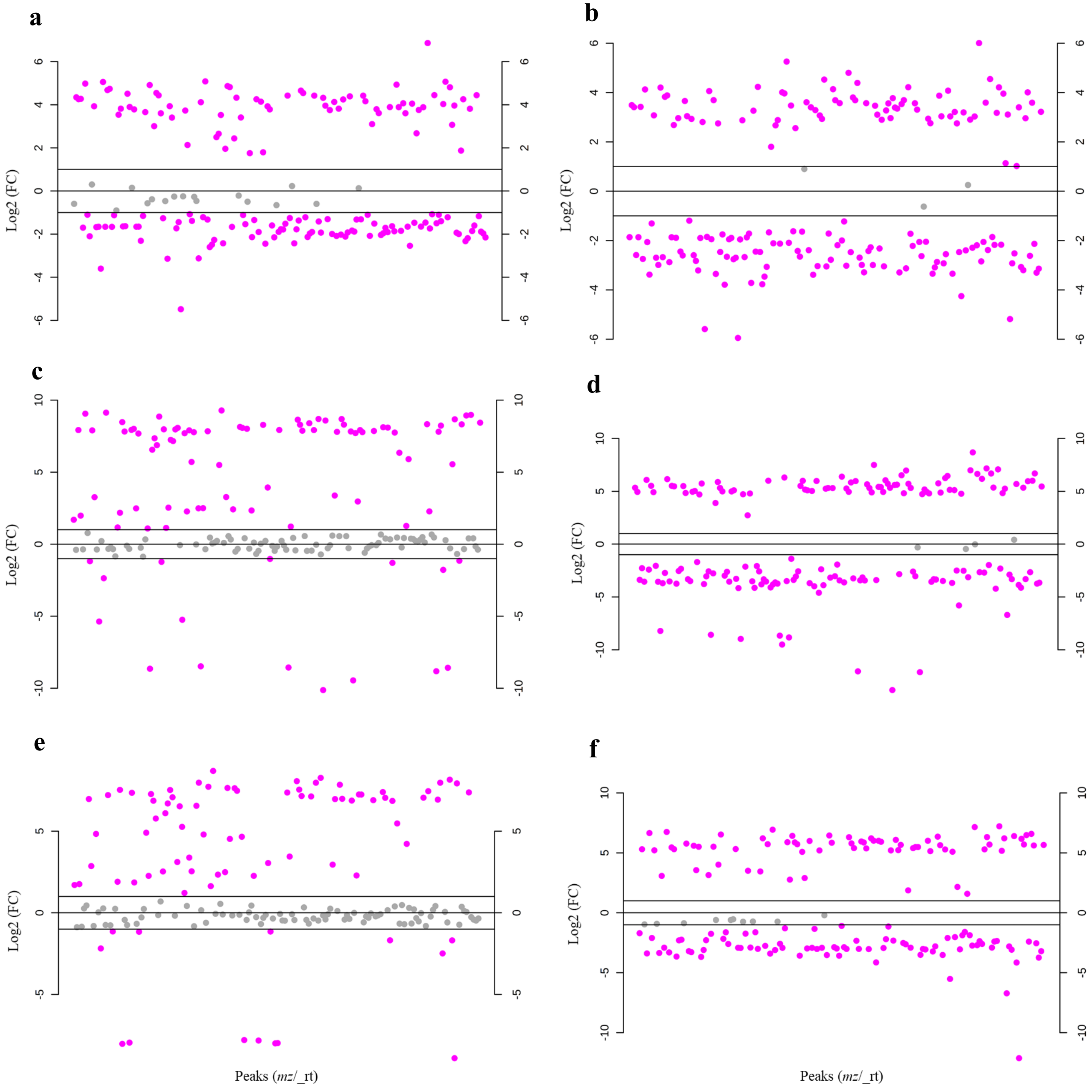

Fig. S5 Identification of differential m/z_rt with FC ≥ 2 in the contrasts performed for ESI^+ ionization mode. (**a-f**) The m/z_rt identified as differential with a cut-off threshold FC ≥ 2 are shown in ESI^+ mode in the following order: T14_27.4 *vs* C16 (**a**); T14_27.4 *vs* C4 (**b**); T21_27.4 *vs* C16 (**c**); T21_27.4 *vs* C4 (**d**); T28_27.4 *vs* C16 (**e**) and T28_27.4 *vs* C4 (**f**)

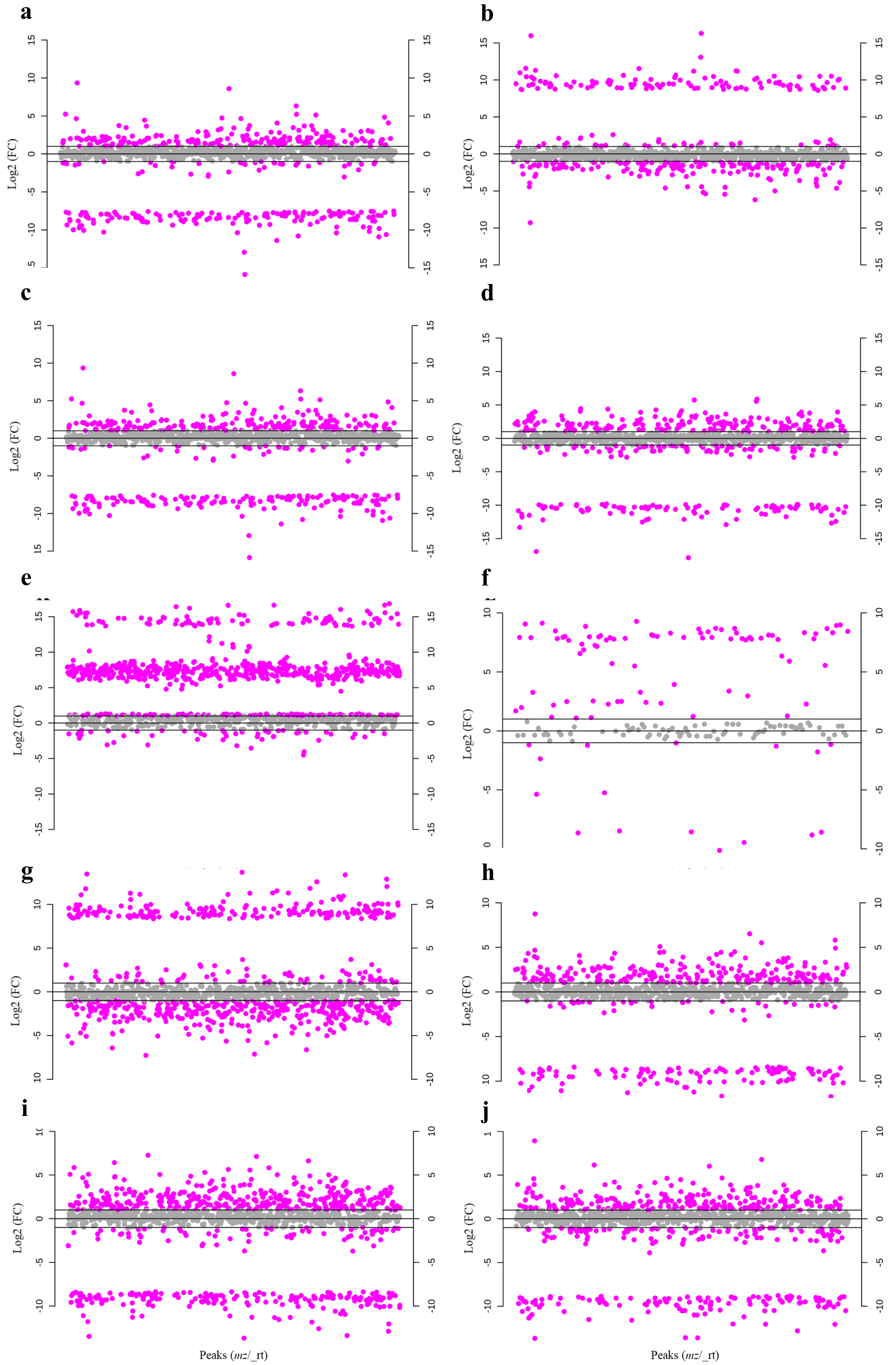

Fig. S6 Identification of differential m/z_rt with FC ≥ 2 in the contrasts performed for ESI⁻ ionization mode. The m/z_rt identified as differential with a cut-off threshold FC ≥ 2 are shown in ESI⁻ mode in the following order: T14_C16 *vs* 27.4 **(a);** T14_C4 *vs* 27.4 **(b);** T14_C27.4 *vs* 16 **(c);** T21_C16 *vs* 27.4 **(d);** T21_C4 *vs* 27.4 **(e);** T21_27.4 *vs* 16 **(f);** T28_16 *vs* 27.4 **(g);** T28_4 *vs* 27.4 **(h);** T28_27.4 *vs* 16 **(i)** and T28_27.4 *vs* 4 **(j)**

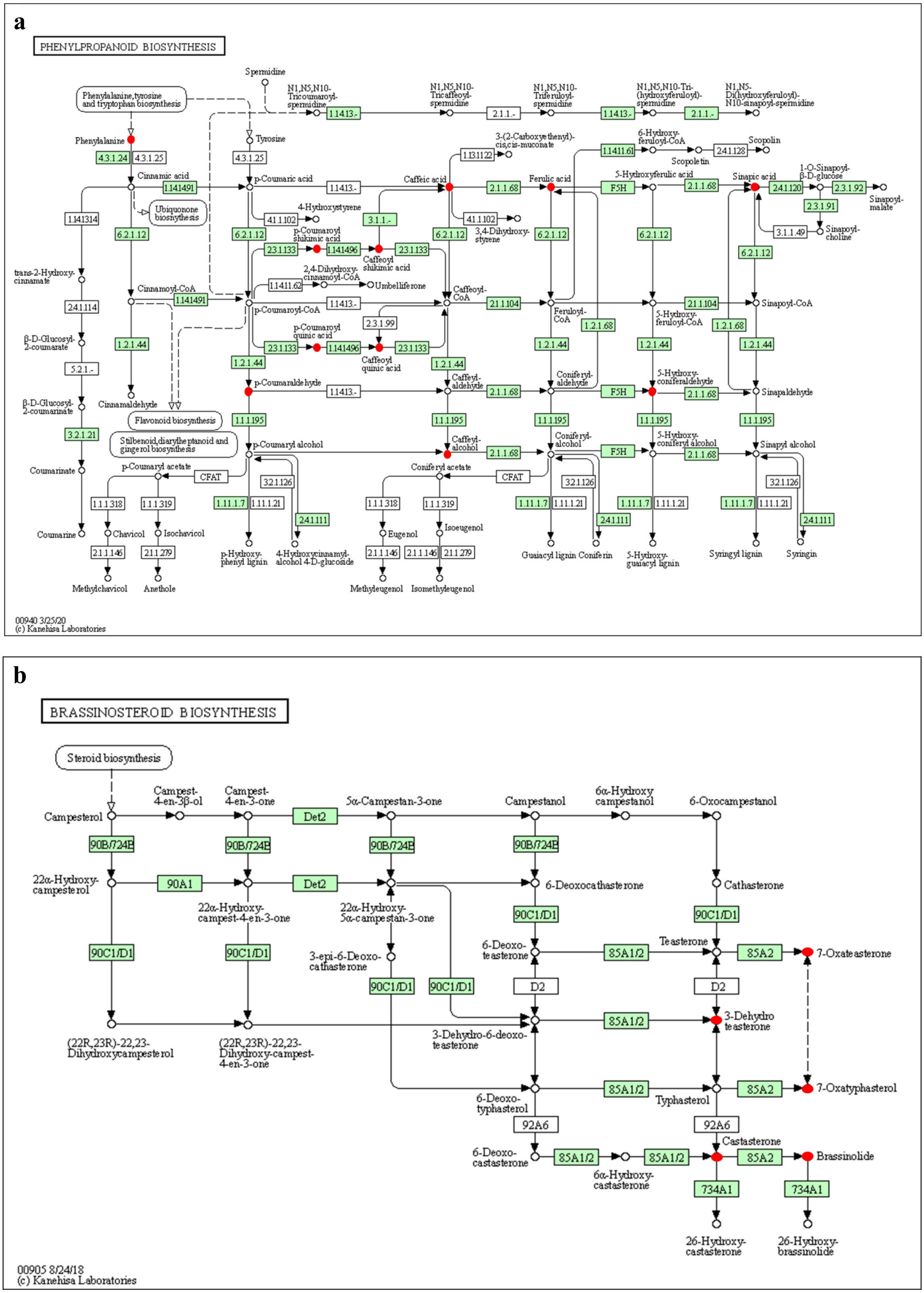

Panels a and b, Fig. S7 Identification of metabolic pathways and their active tentative metabolites (tME) within them in ESI^{+} mode

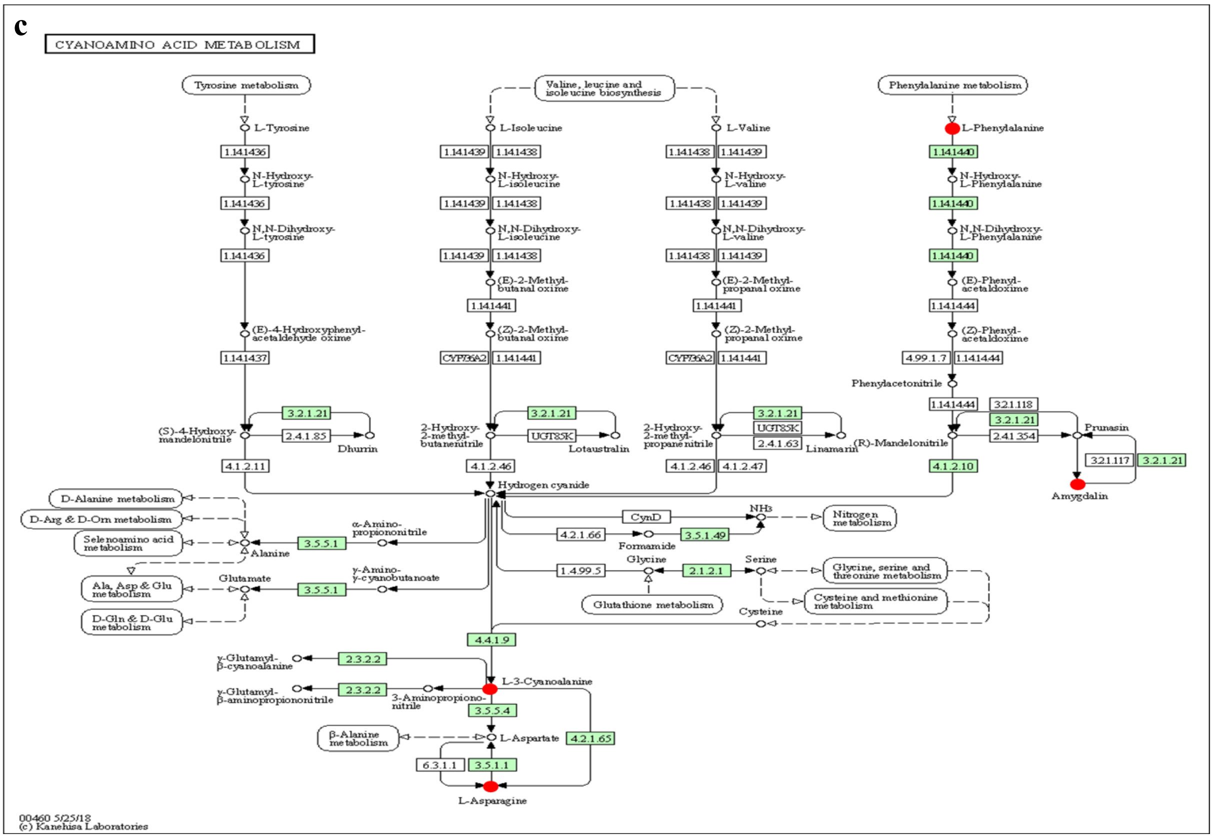

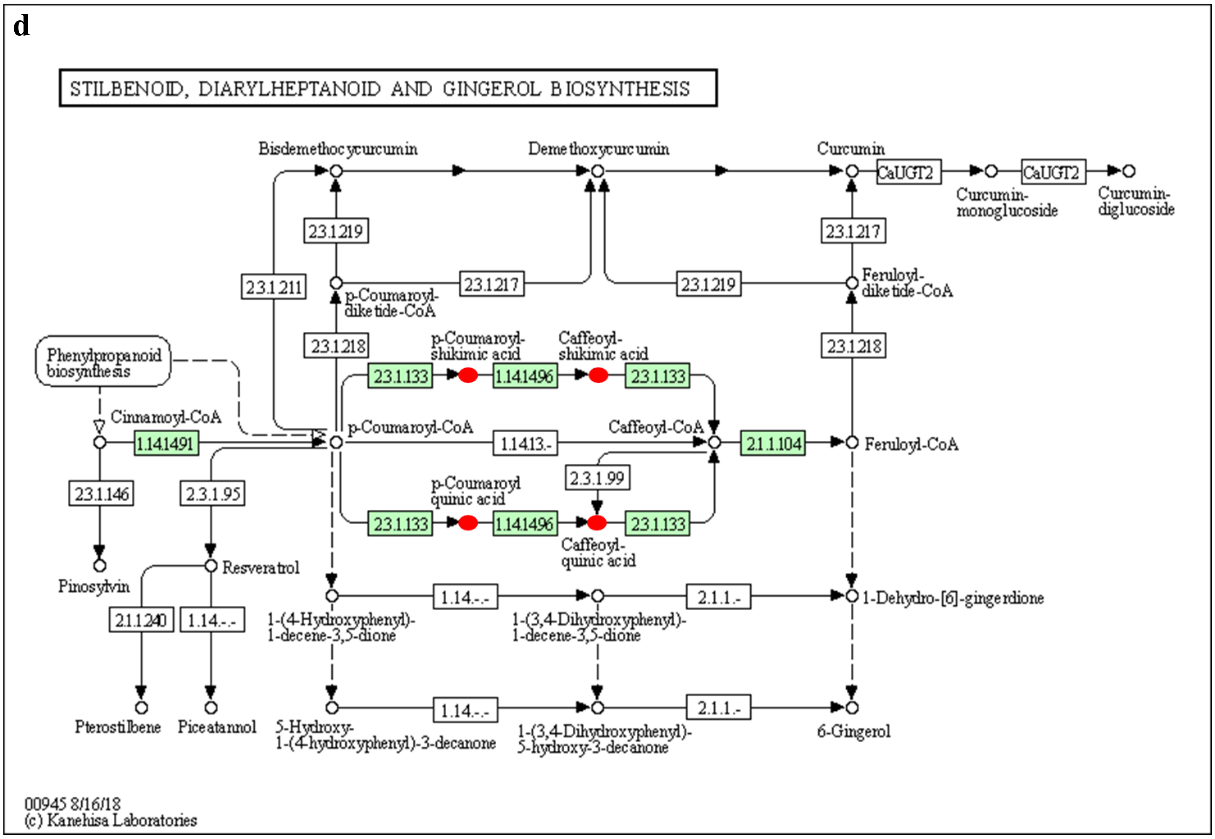

Panels c and d, Fig S7 Identification of metabolic pathways and their active tentative metabolites (tME) within them in ESI^{+} mode

e

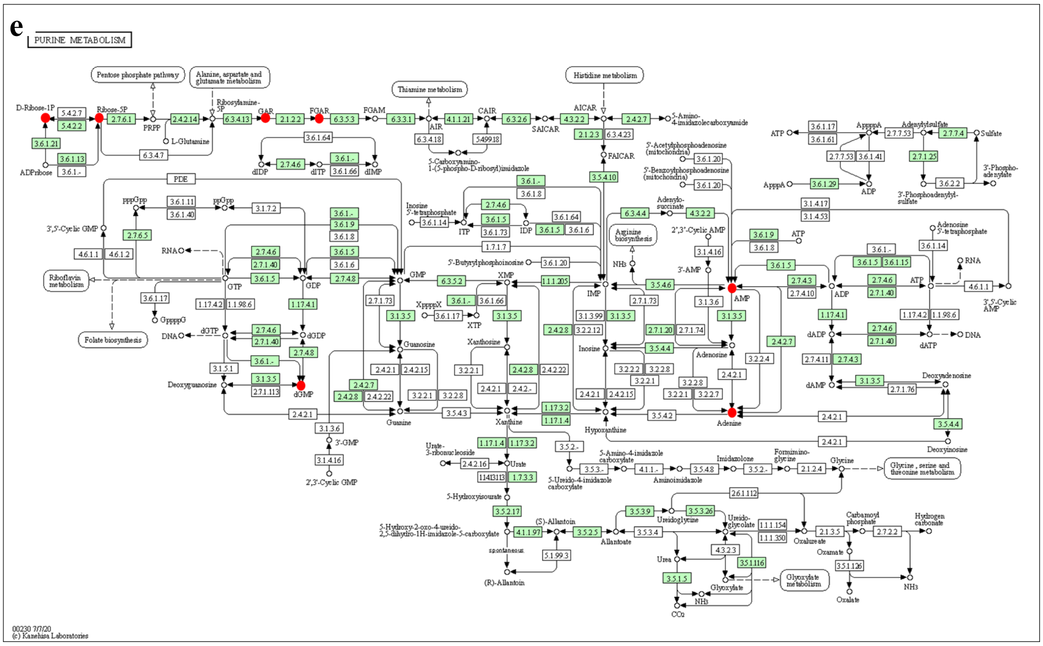

f

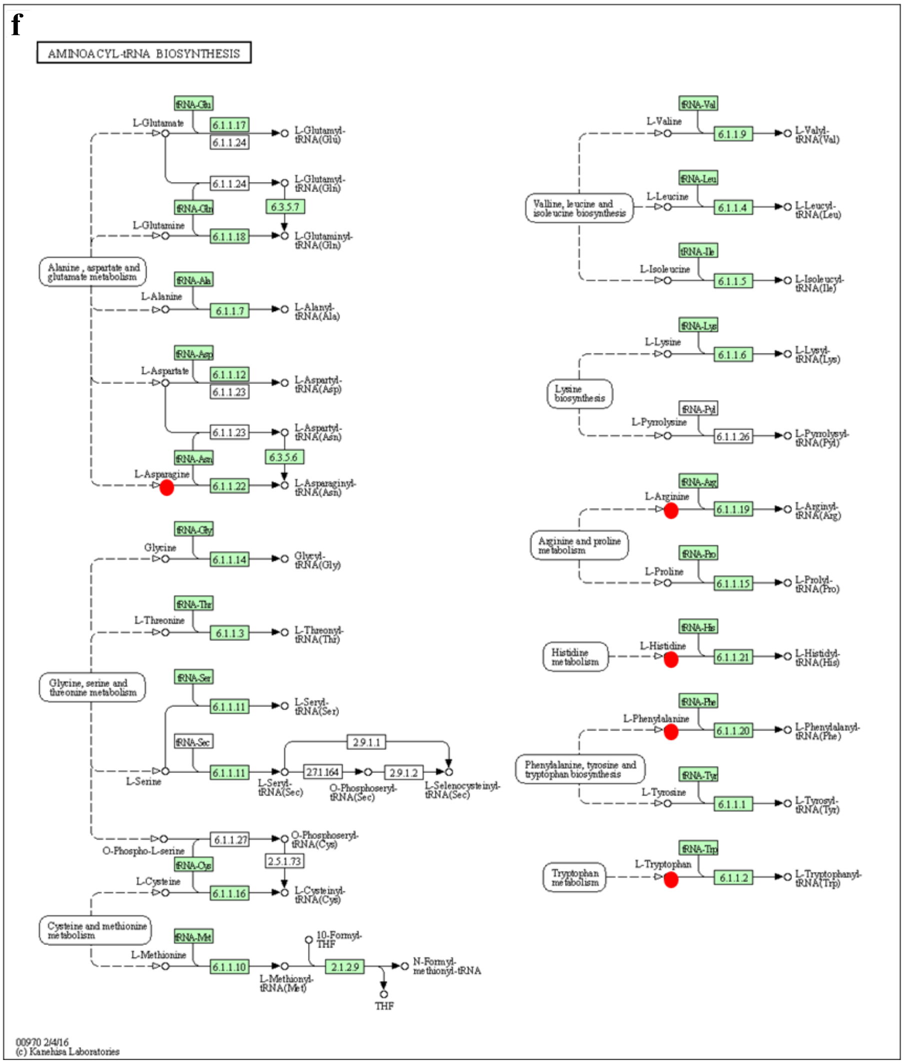

Panels e and f, Fig. S7 Identification of metabolic pathways and their active tentative metabolites (tME) within them in ESI$^+$ mode.

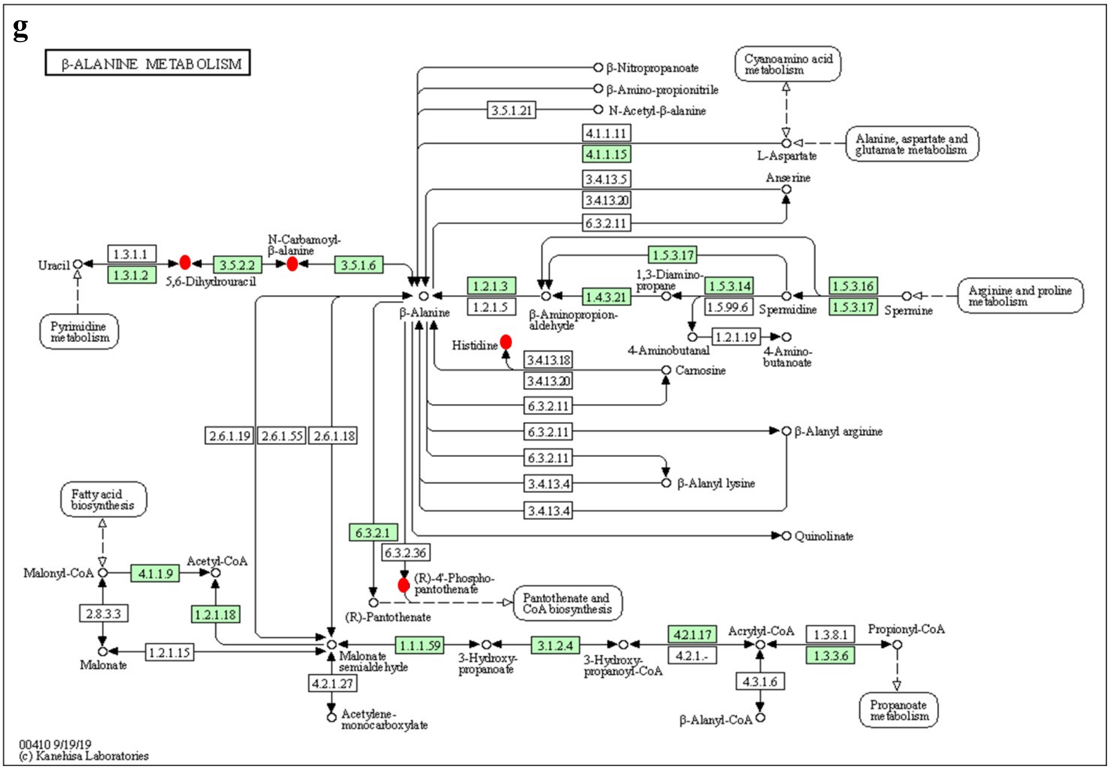

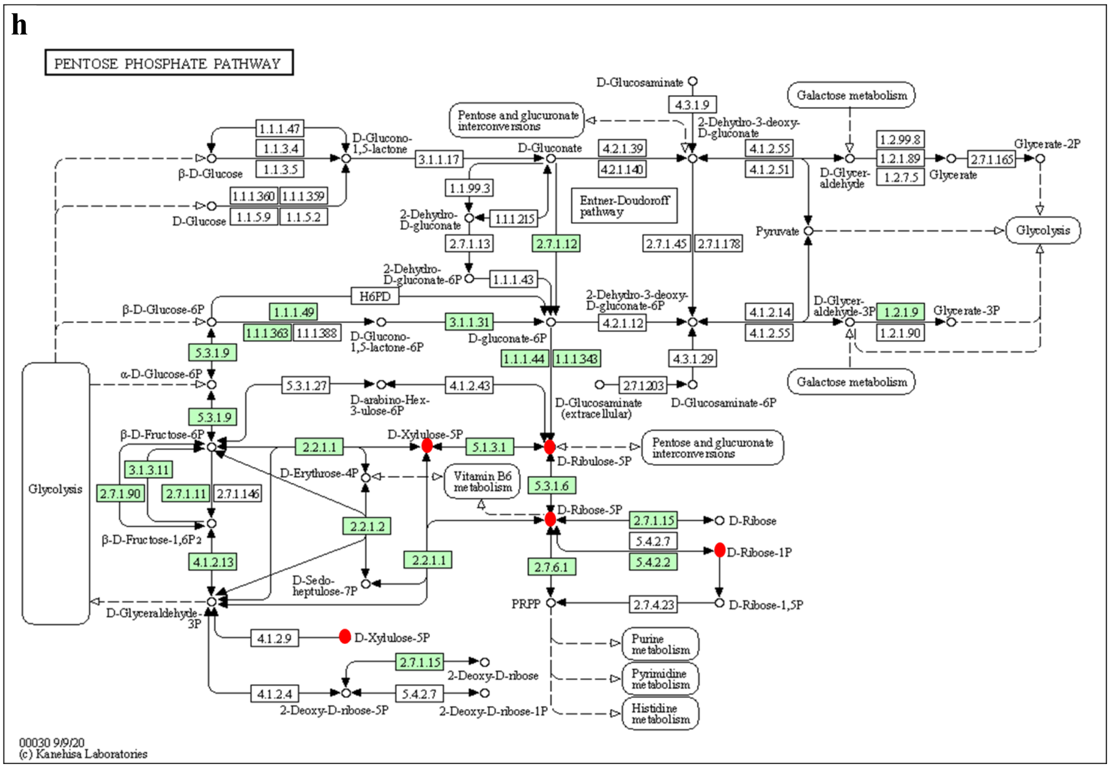

Panels g and h, Fig. S7 Identification of metabolic pathways and their active tentative metabolites (tME) within them in ESI$^+$ mode

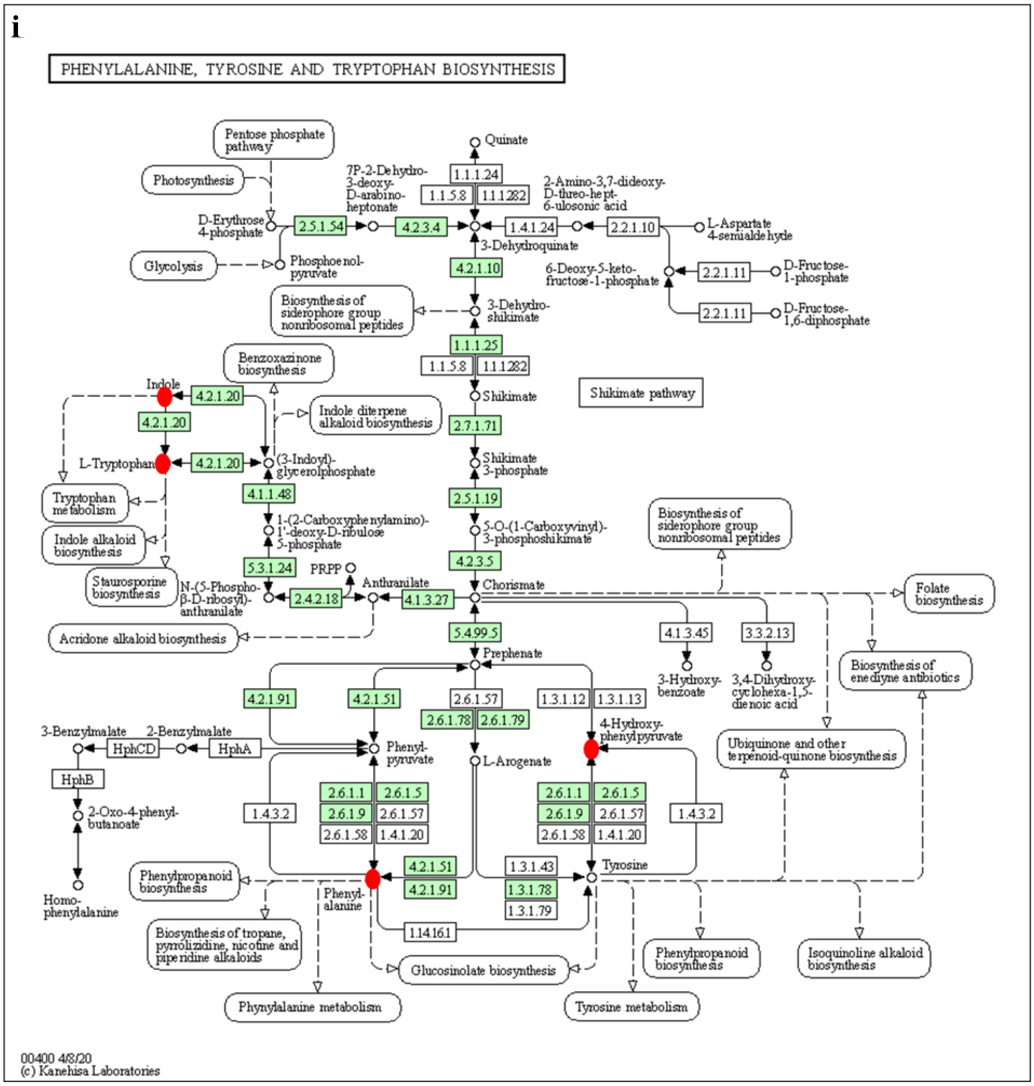

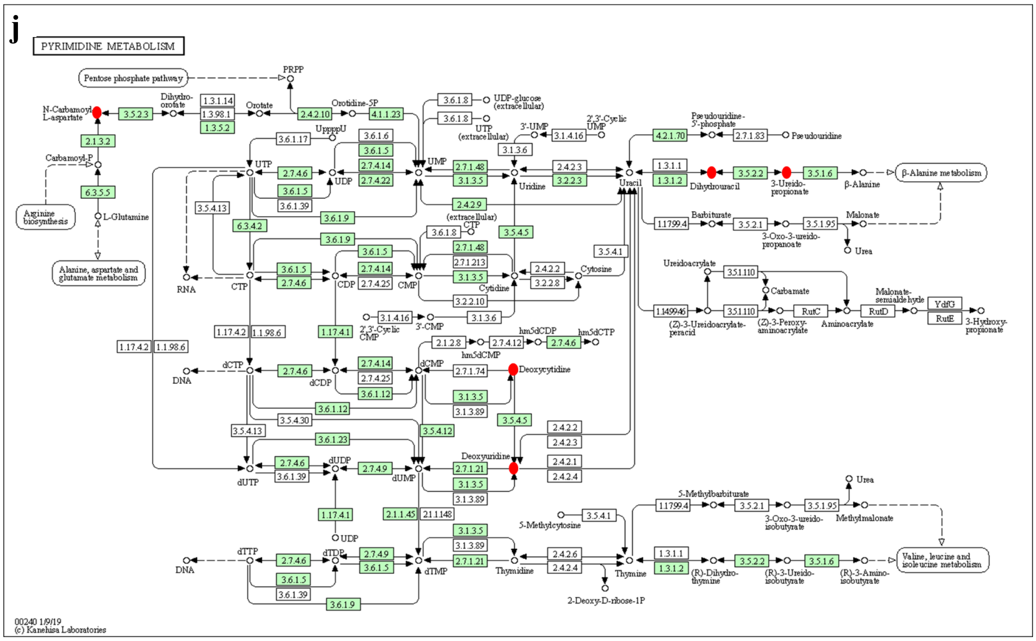

Panels i and j, Fig. S7 Identification of metabolic pathways and their active tentative metabolites (tME) within them in ESI^{+} mode

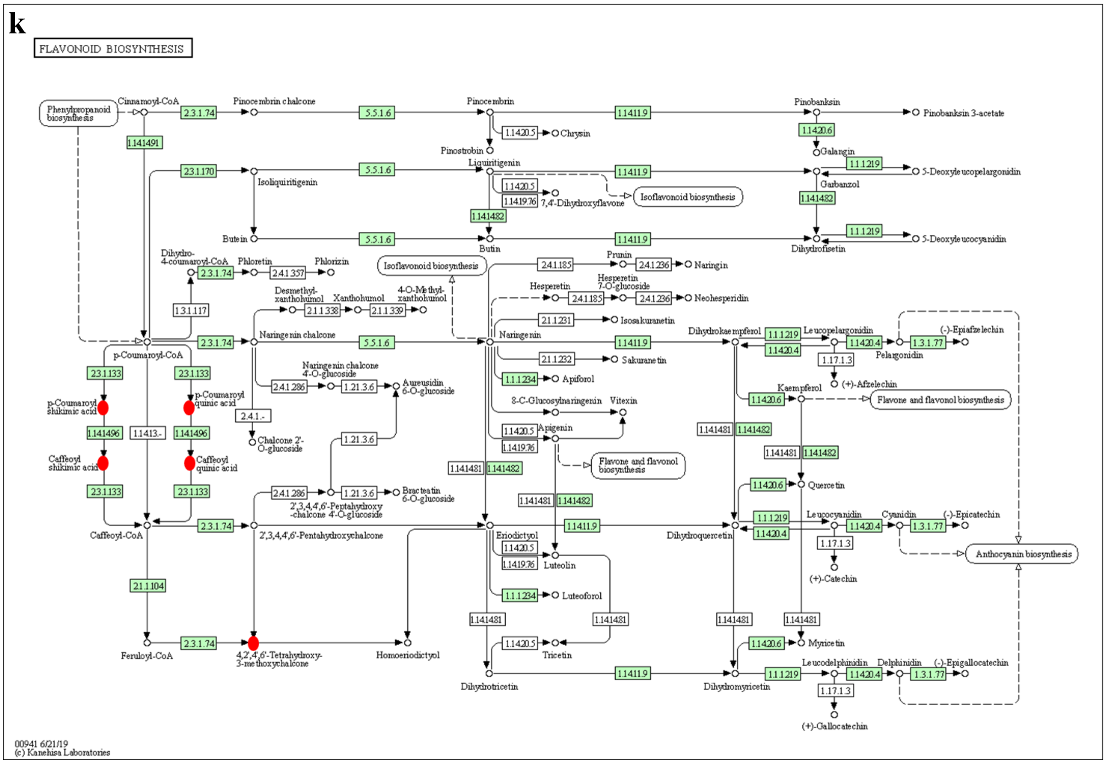

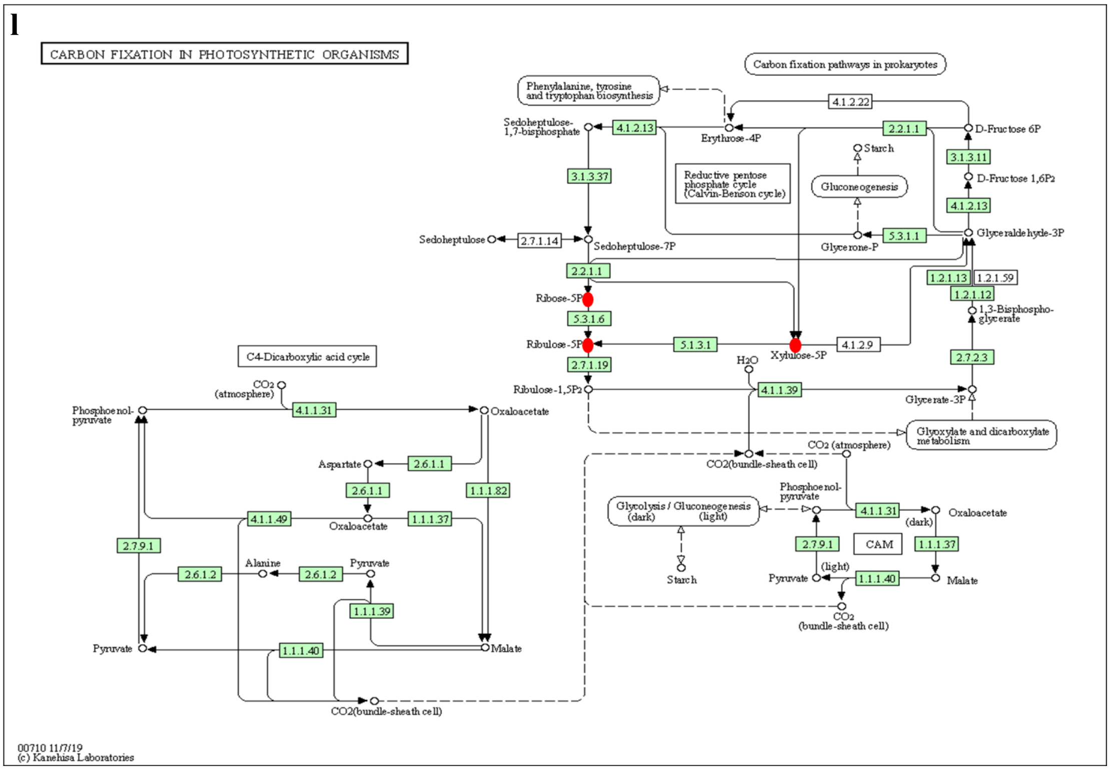

Panels k and l, Fig. S7 Identification of metabolic pathways and their active tentative metabolites (tME) within them in ESI$^+$ mode

m

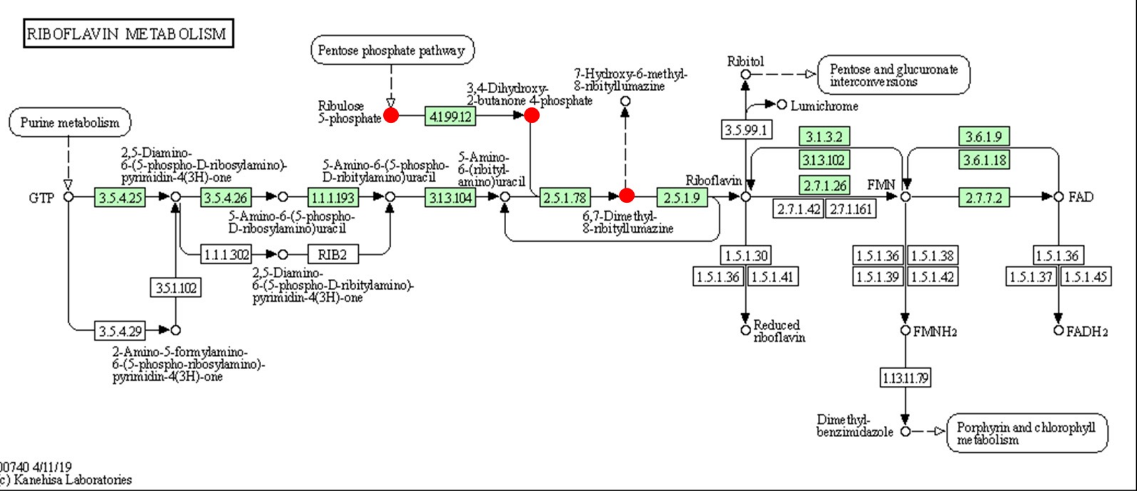

n

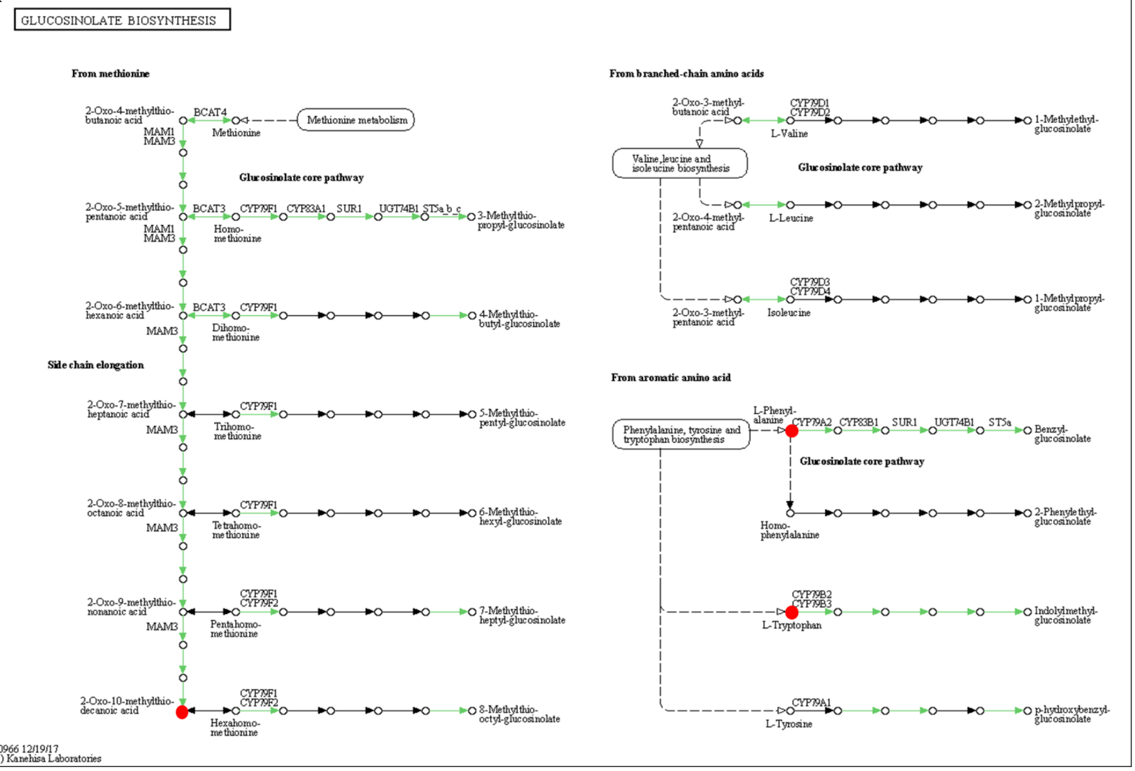

Panels m and n, Fig. S7 Identification of metabolic pathways and their active tentative metabolites (tME) within them in ESI$^+$ mode

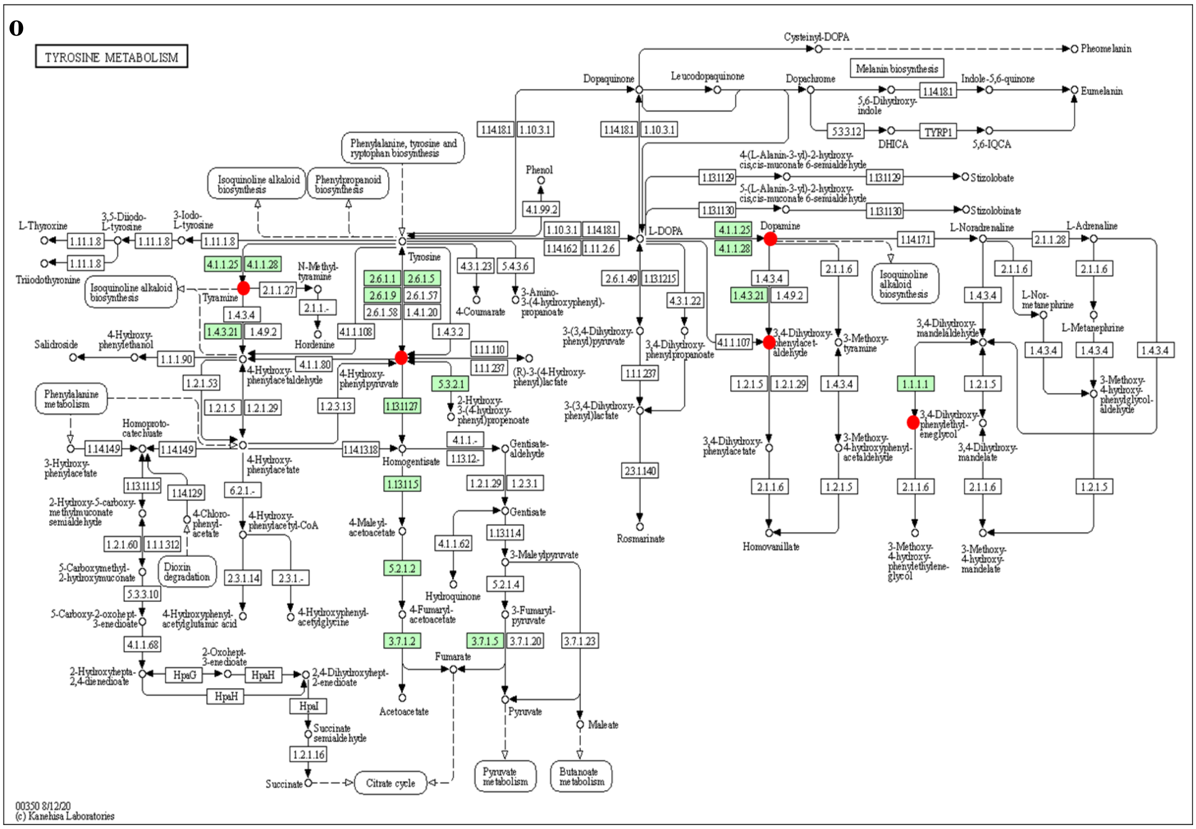

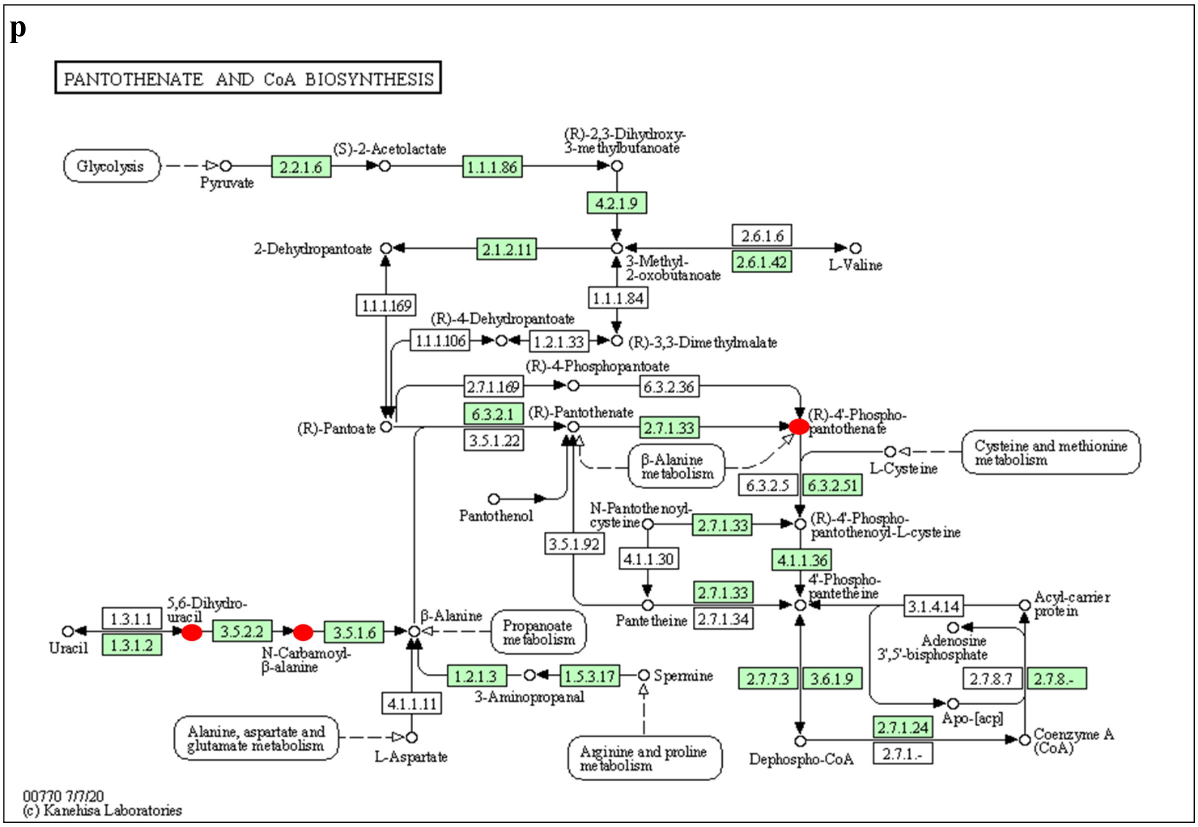

Panels o and p, Fig S7 Identification of metabolic pathways and their active tentative metabolites (tME) within them in ESI$^+$ mode

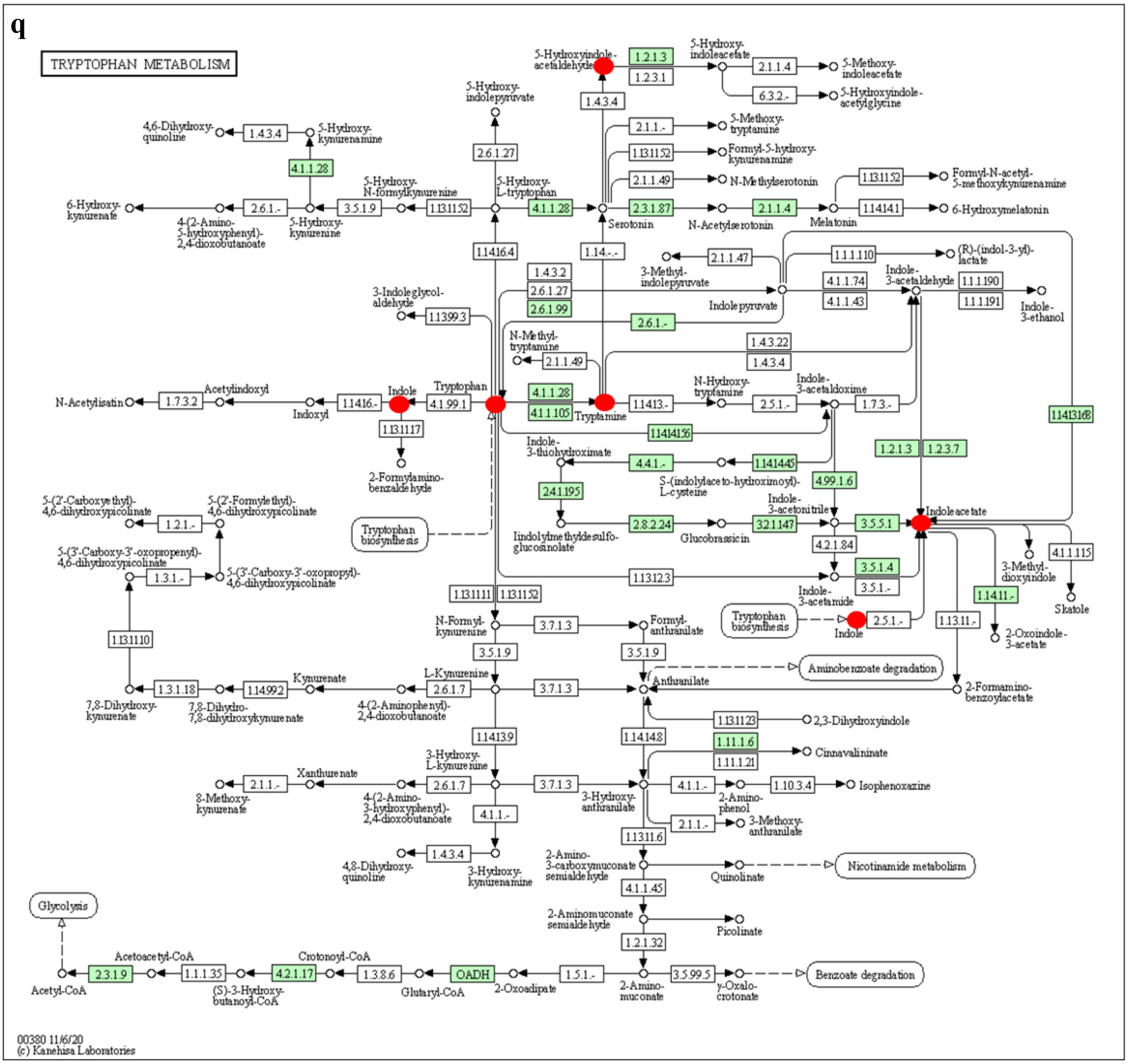

Fig. S7 Identification of metabolic pathways and their active tentative metabolites (tME) within them in ESI$^+$ mode. All the active metabolites identified in this analysis are colored in red (**a**) Phenylpropanoid biosynthesis (11 active SMe) (**b**) Brassinosteroid biosynthesis (5 active SMe) (**c**) Cyanoamino acid metabolism (4 active SMe) (**d**) Stilbenoid, diarylheptanoid and gingerol biosynthesis (4 active SMe) (**e**) Purine metabolism (7 active SMe) (**f**) Aminoacyl-tRNA biosynthesis (5 active SMe) (**g**) beta-Alanine metabolism (4 active SMe) (**h**) Pentose phosphate pathway (4 active SMe) (**i**) Phenylalanine, tyrosine and tryptophan biosynthesis (4 active SMe) (**j**) Pyrimidine metabolism (5 active SMe) (**k**) Flavonoid biosynthesis (5 active SMe) (**l**) Carbon fixation in photosynthetic organisms (3 active SMe) (**m**) Riboflavin metabolism (3 active SMe) (**n**) Glucosinolate biosynthesis (3 active SMe) (**o**) Tyrosine metabolism (5 active SMe) (**p**) Pantothenate and CoA biosynthesis (3 active SMe) (**q**) Tryptophan metabolism (5 active SMe)

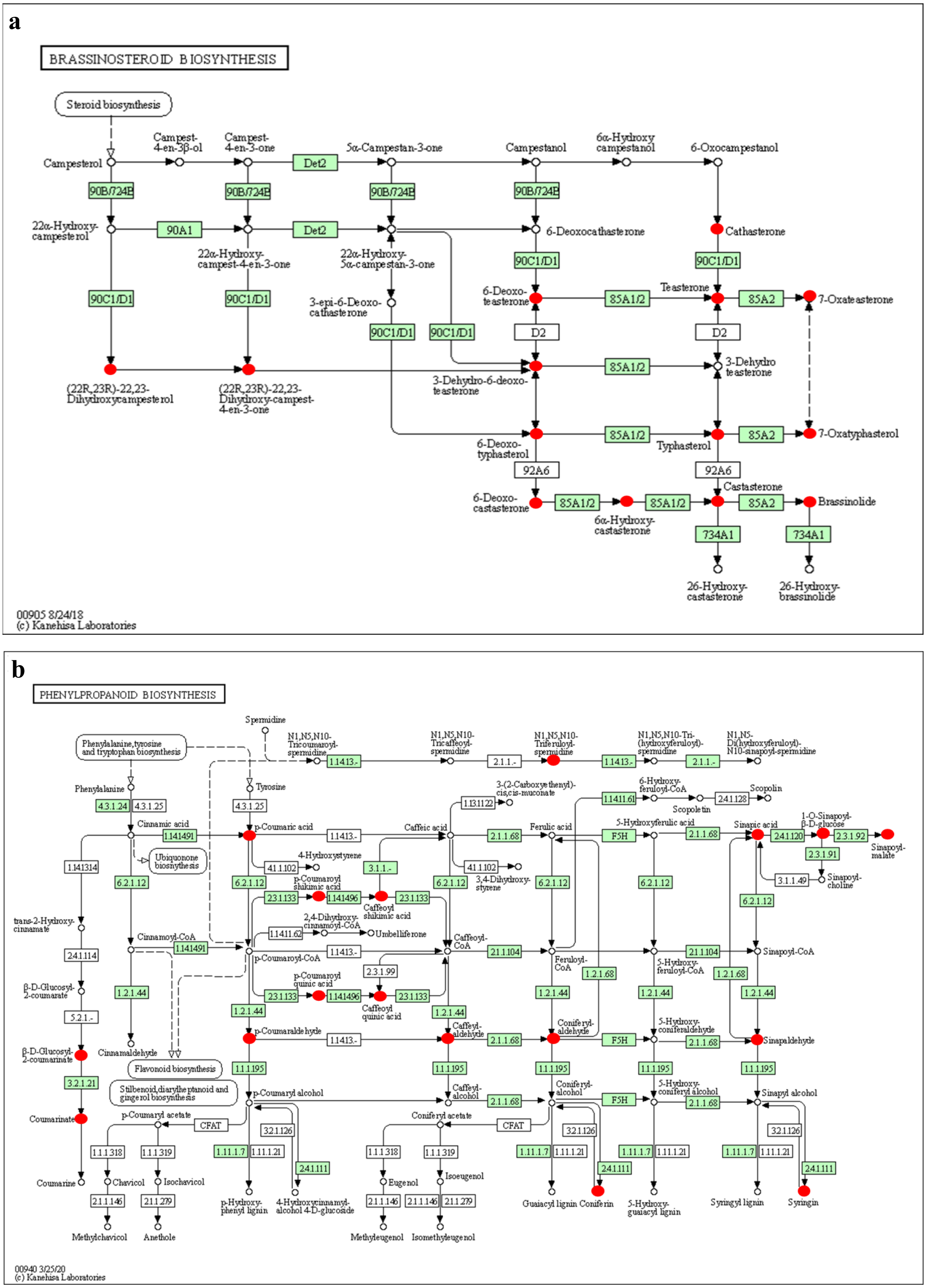

Panels a and b, Figure S8 Identification of metabolic pathways and their tentative metabolites (tME) within them in ESI$^-$ mode

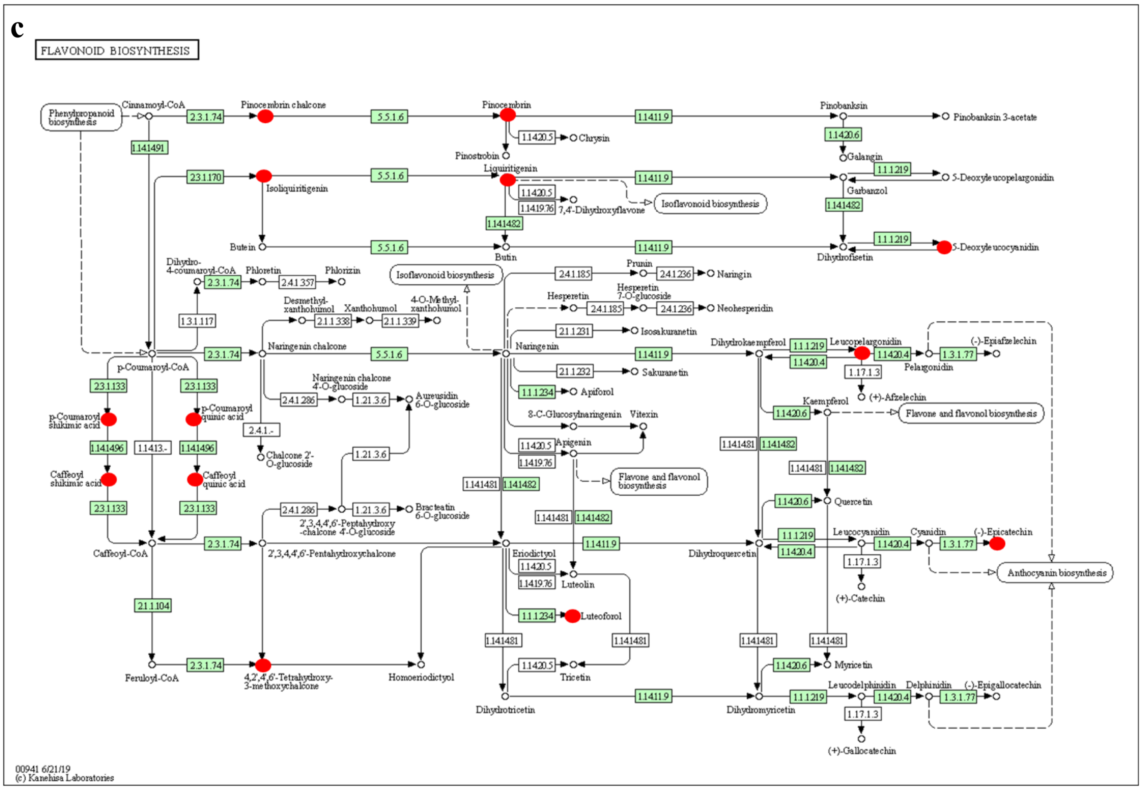

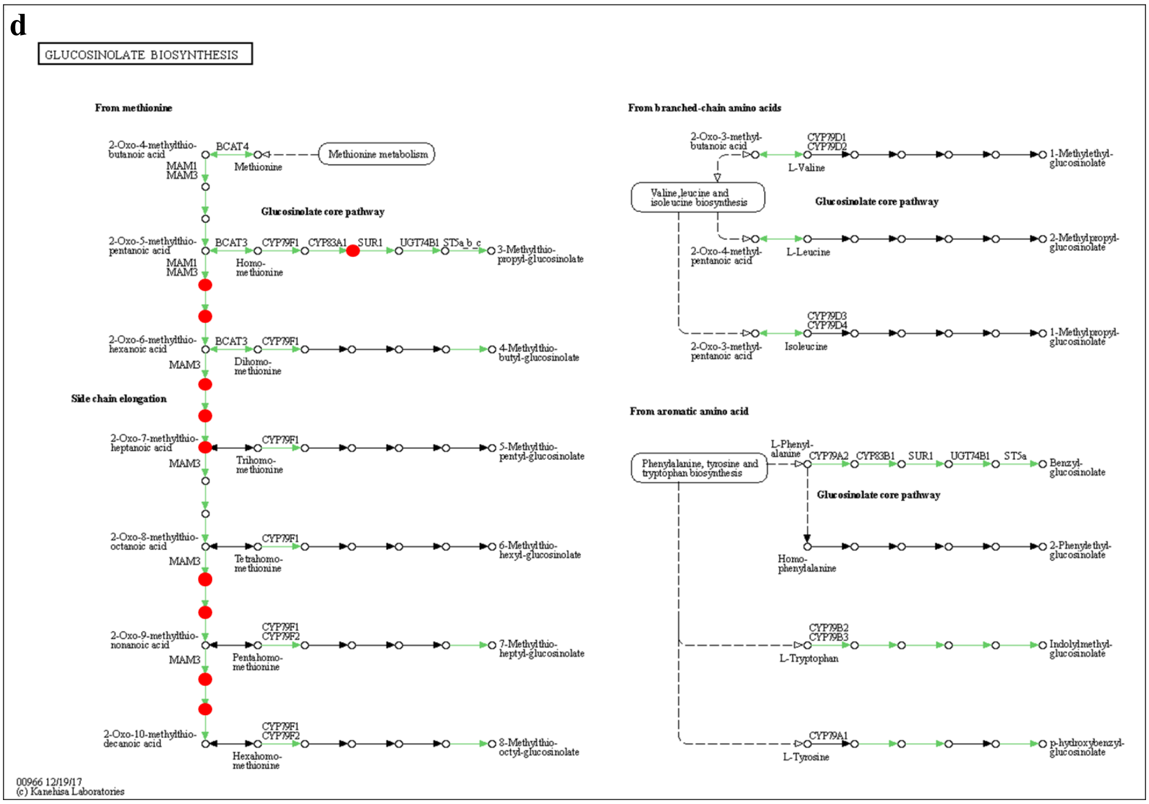

Panels c and d, Fig. S8 Identification of metabolic pathways and their tentative metabolites (tME) within them in ESI$^-$ mode

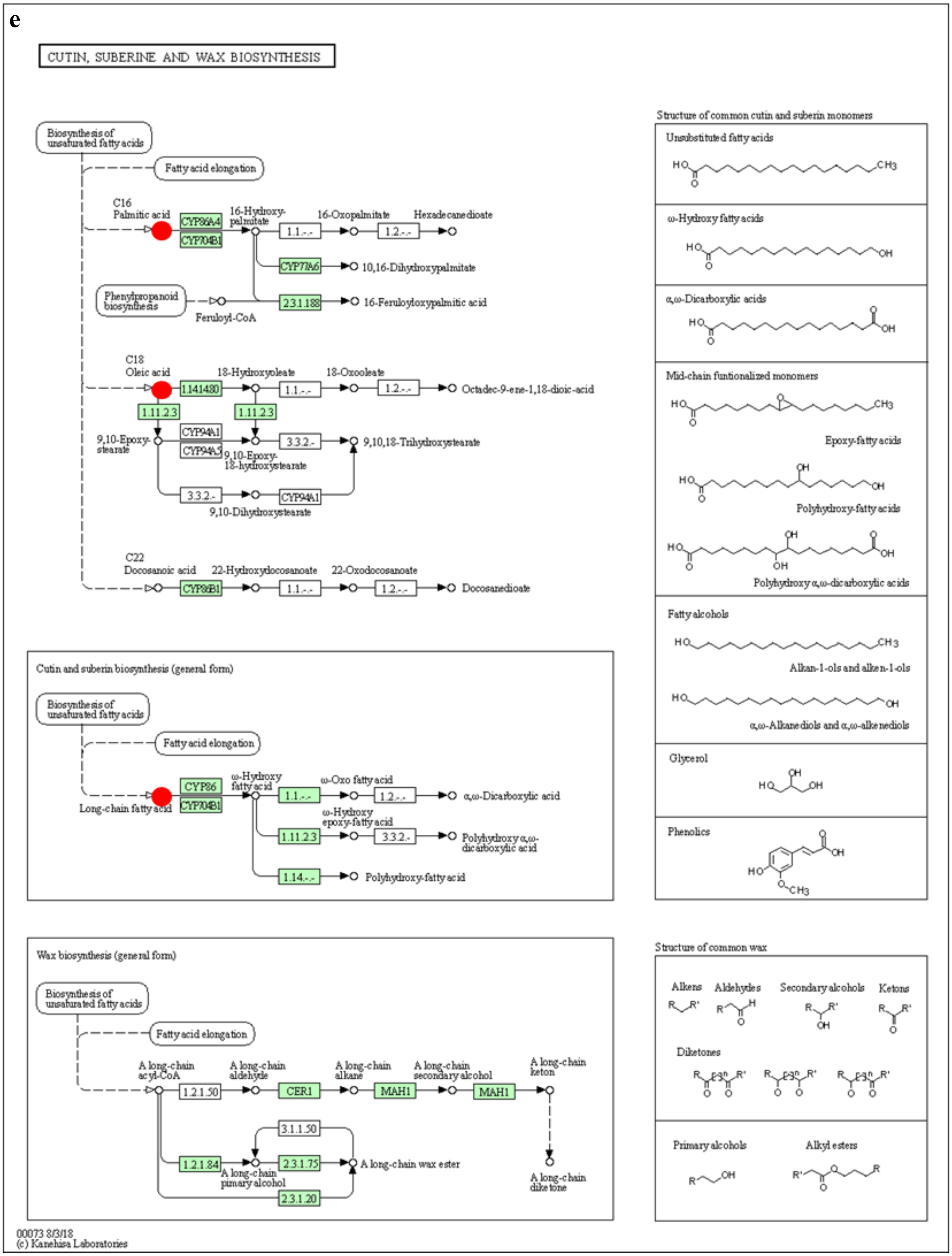

Panel e, Fig. S8 Identification of metabolic pathways and their tentative metabolites (tME) within them in ESI$^-$ mode

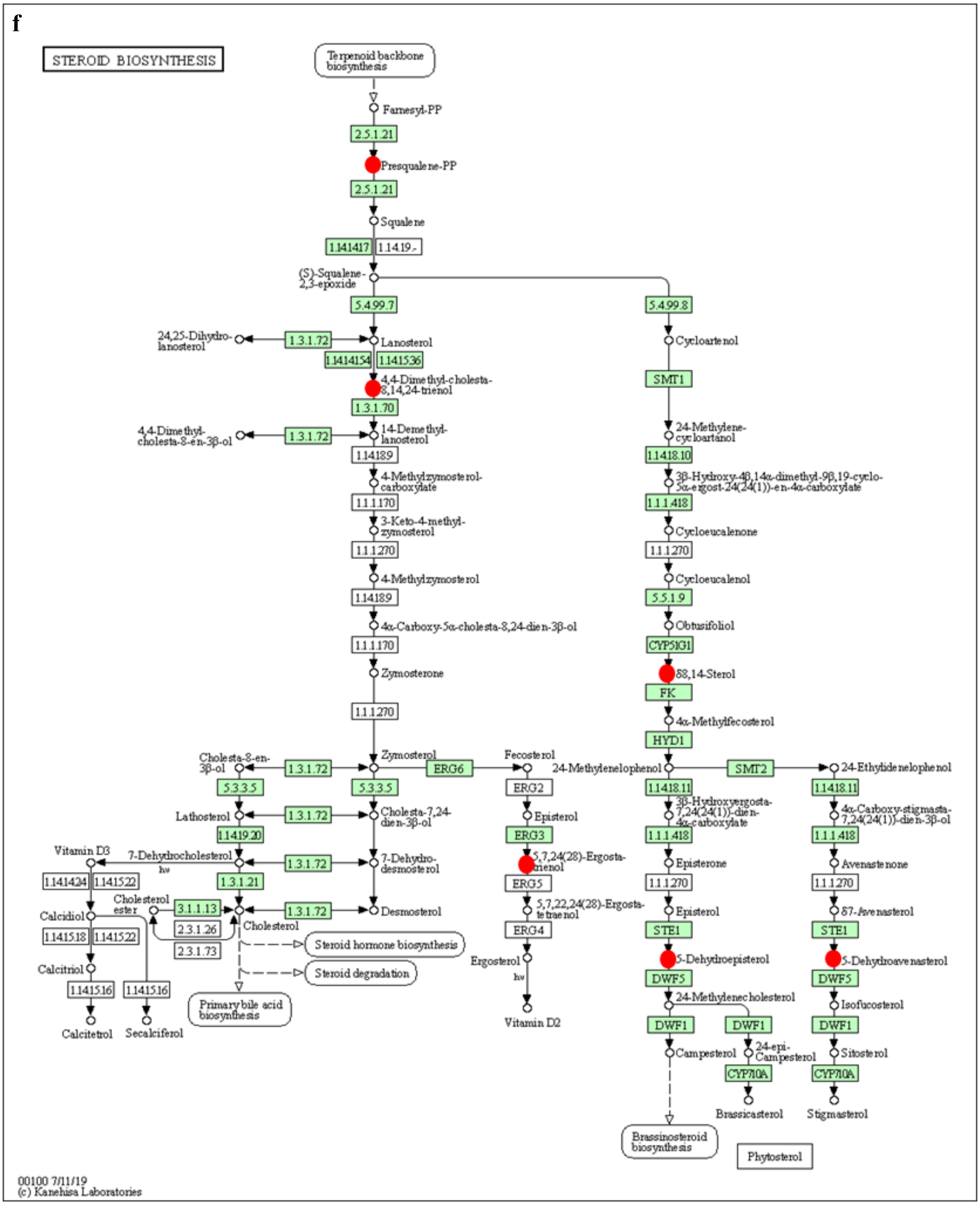

Panel f, Fig. S8 Identification of metabolic pathways and their tentative metabolites (tME) within them in ESI⁻ mode

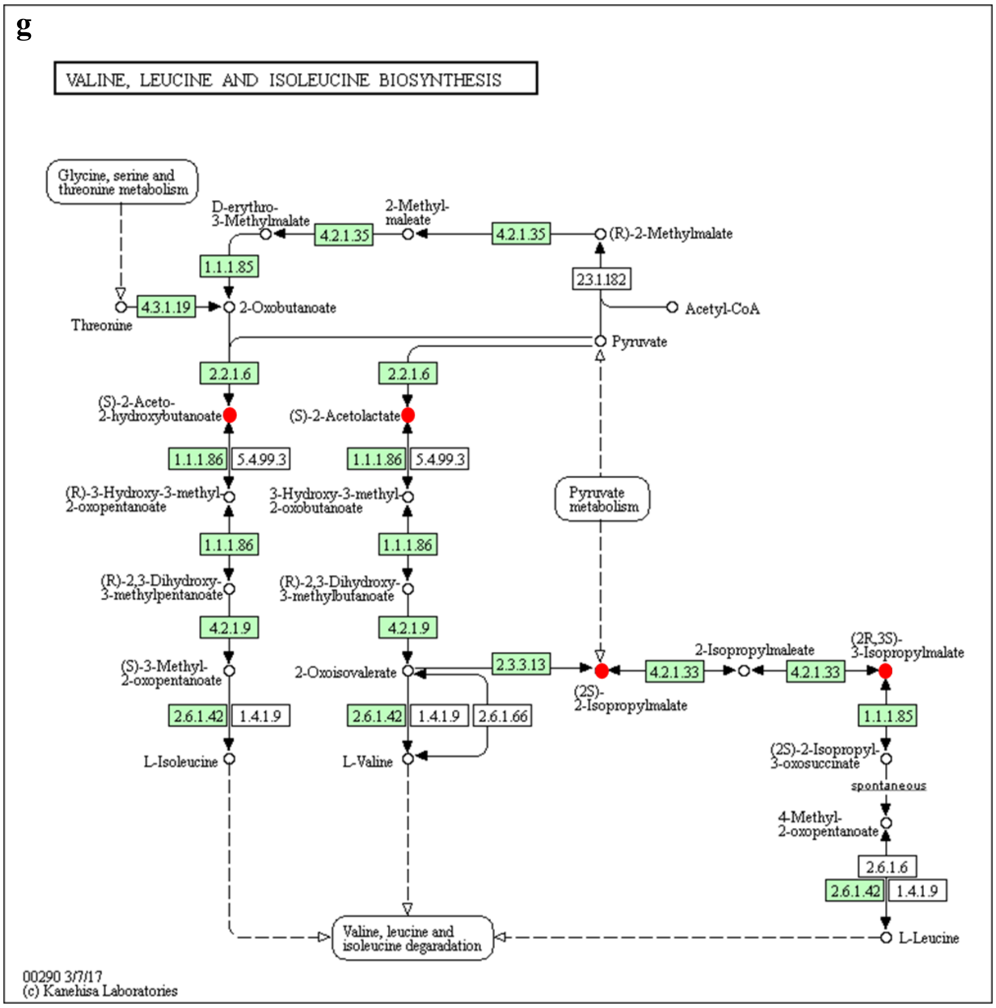

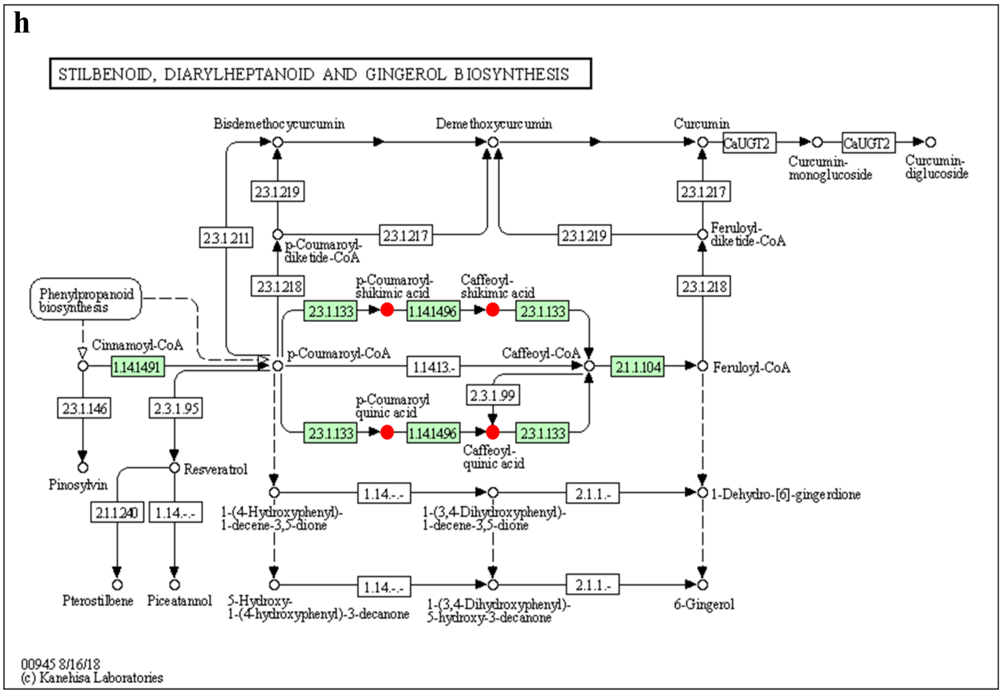

Panels g and h, Fig. S8 Identification of metabolic pathways and their tentative metabolites (tME) within them in ESI$^-$ mode

i

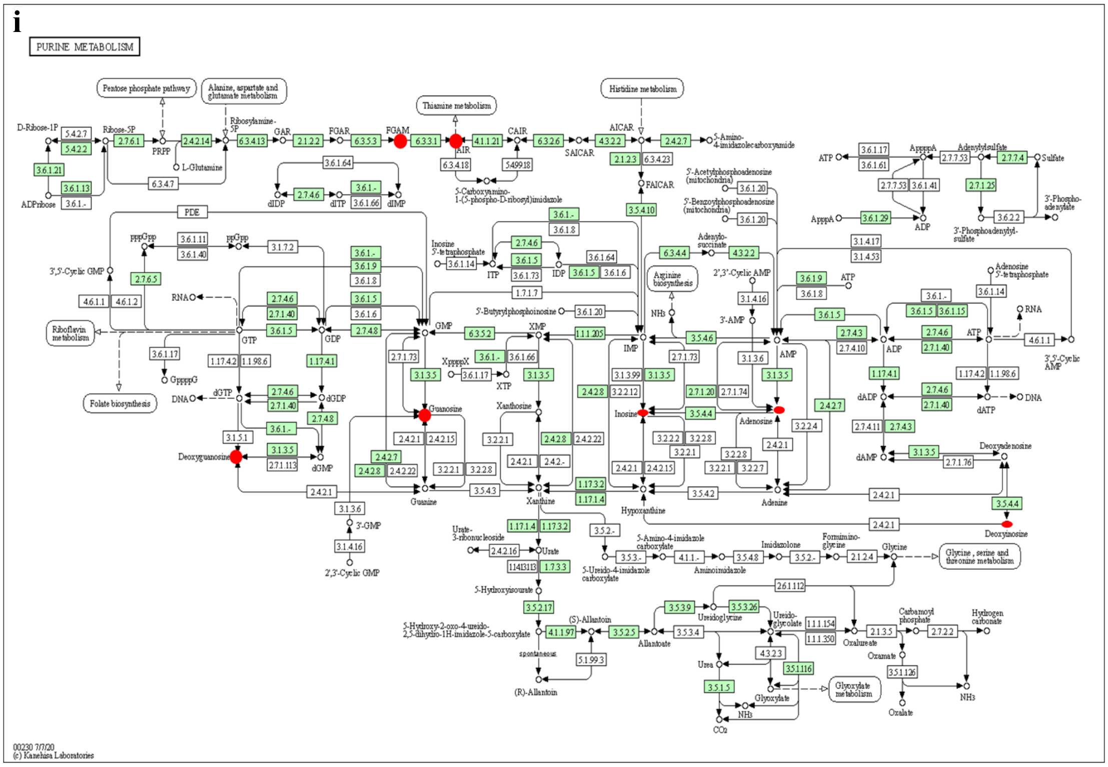

k

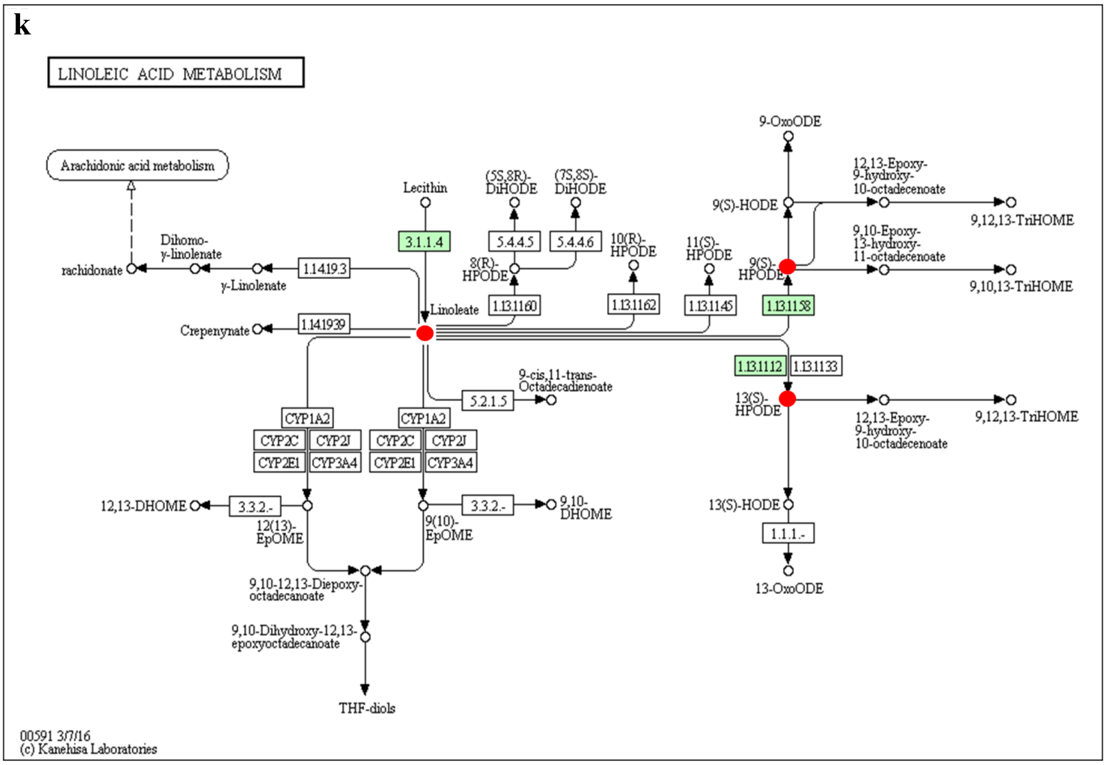

Panels i and k, Fig. S8 Identification of metabolic pathways and their tentative metabolites (tME) within them in ESI^{-} mode

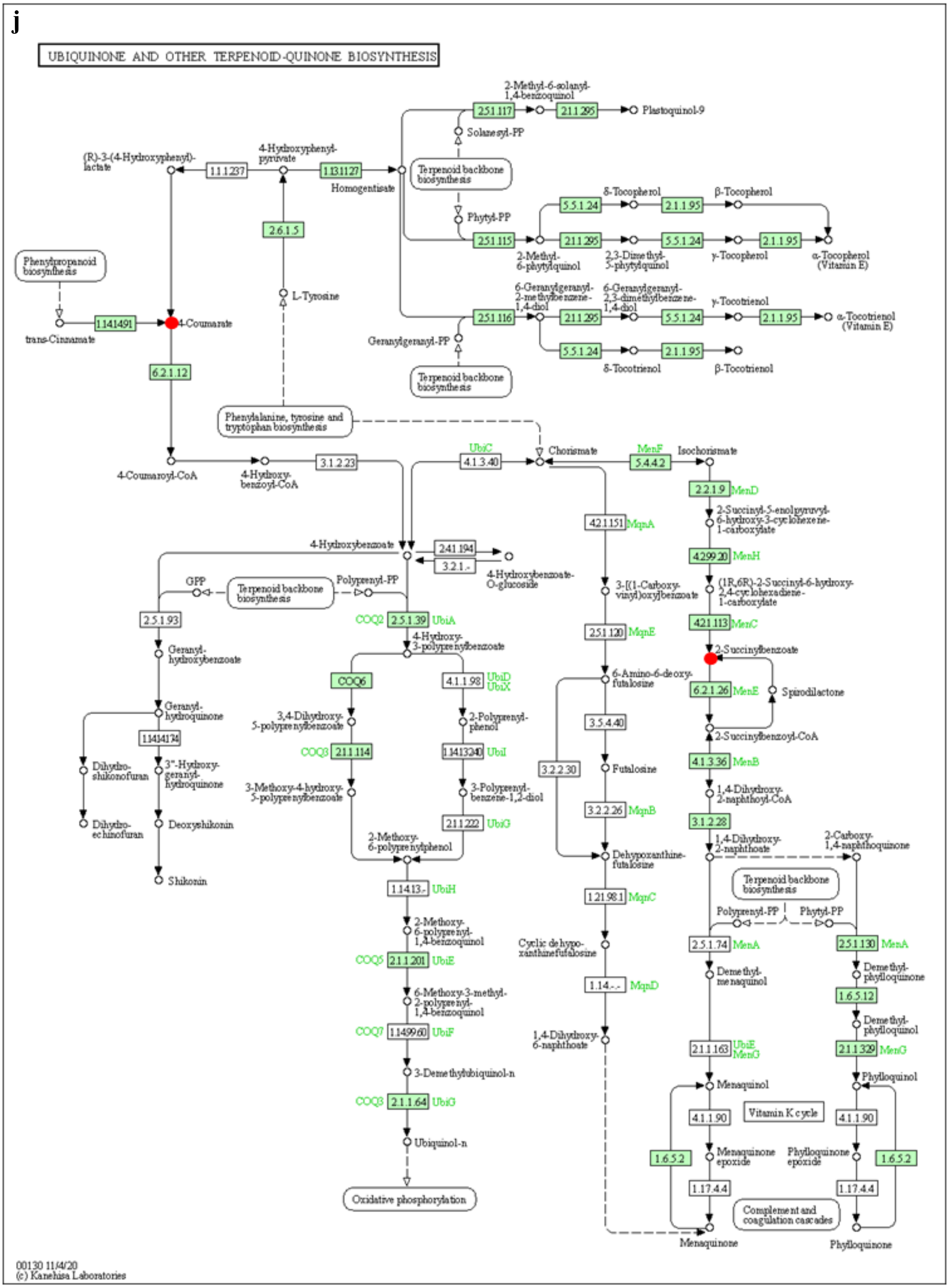

Panel j, Fig. S8 Identification of metabolic pathways and their tentative metabolites (tME) within them in ESI$^-$ mode

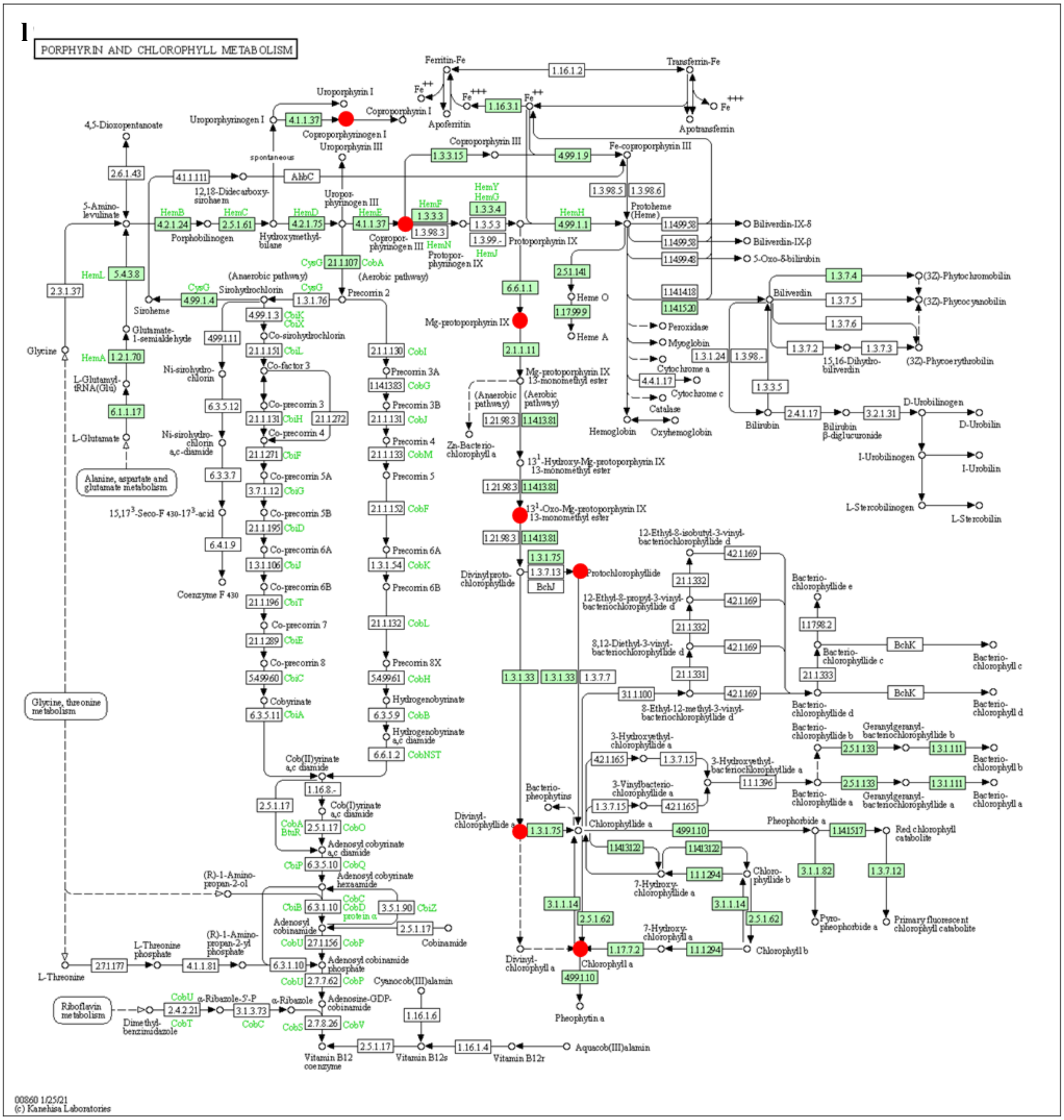

Fig. S8 Identification of metabolic pathways and their tentative metabolites (tME) within them in ESI$^-$ mode. All the active metabolites identified in this analysis are colored in red. (**a**) Brassinosteroid biosynthesis (14 active SMe) (**b**) Phenylpropanoid biosynthesis (17 active SMe) (**c**) Flavonoid biosynthesis (13 active SMe) (**d**) Glucosinolate biosynthesis (10 active SMe) (**e**) Cutin, suberine and wax biosynthesis (2 active SMe) (**f**) Steroid biosynthesis (6 active SMe) (**g**) Valine, leucine and isoleucine biosynthesis (4 active SMe) (**h**) Stilbenoid, diarylheptanoid and gingerol biosynthesis (4 active SMe) (**i**) Purine metabolism (7 active SMe) (**j**) Ubiquinone and other terpenoid-quinone biosynthesis (2 active SMe) (**k**) Linoleic acid metabolism (3 active SMe) (**l**) Porphyrin and chlorophyll metabolism (7 active SMe)
